# Anticonvulsant effects, safety, and sex-dependent molecular signatures of cannabidiol treatment in a preclinical model of audiogenic epilepsy

**DOI:** 10.3389/fphar.2026.1745696

**Published:** 2026-08-07

**Authors:** Giselda Cabral-Pereira, Dolores E. López, Ricardo Gómez-Nieto

**Affiliations:** 1 Instituto de Neurociencias de Castilla y León (INCYL), Universidad de Salamanca, Salamanca, Spain; 2 Instituto de Investigación Biomédica de Salamanca (IBSAL), Universidad de Salamanca, Salamanca, Spain; 3 Instituto de Neurociencias UMH-CSIC, Universidad Miguel Hernández and Consejo Superior de Investigaciones Científicas, Sant Joan d’Alacant, Spain; 4 Departamento de Biología Celular y Patología, Facultad de Medicina, Universidad de Salamanca, Salamanca, Spain

**Keywords:** animal models, anticonvulsant therapy, cannabis, epilepsy, gene expression, neuroethology, pharmacokinetics, sex differences

## Abstract

Roughly one-third of patients with epilepsy remain drug resistant, underscoring the need for novel therapeutic strategies. Although cannabidiol (CBD) has recently been approved for specific epileptic encephalopathies, its anticonvulsant mechanisms and the influence of biological sex on treatment response remain incompletely understood. In this preclinical study, we evaluated the efficacy, tolerability, pharmacokinetics, and molecular effects of CBD in the GASH/Sal hamster, a genetic model of audiogenic generalized tonic–clonic seizures. Animals received intraperitoneal CBD (200 mg/kg) either acutely or chronically for 14 days. CBD concentrations were measured in serum and brain, while seizure severity, latency, and neuroethological parameters were assessed following acoustic seizure induction. Safety was evaluated through body weight, hematological, and biochemical analyses, and gene expression profiling was performed in the inferior colliculus, the primary epileptogenic focus. CBD achieved measurable systemic and brain exposure after both acute and chronic administration, despite substantial inter-individual variability and no significant sex differences in drug concentrations. CBD reduced audiogenic seizure severity in a time-dependent manner, with greater protection after chronic treatment. CBD also prolonged latency to seizure onset in both sexes (earlier in females). Although complete seizure suppression was more frequent in females than males (37.5% vs. 12.5% after chronic treatment), direct sex comparisons did not reach statistical significance. Notably, higher serum and brain CBD concentrations were associated with lower seizure severity. Chronic CBD administration was well tolerated in both sexes, affecting selected hematological parameters without altering body weight or liver function. Gene expression profiling revealed that transcriptional organization of the inferior colliculus was driven predominantly by biological sex rather than by seizure induction or CBD treatment. Marked sex-dependent differences were observed in serotonergic, endocannabinoid, purinergic, and Sigmar1-related neuroprotective pathways. Within this molecular context, seizure stimulation modulated *Trpv1* and *Slc29a1*, whereas chronic CBD induced pathway-specific, sex-dependent changes involving *5-Htr1a*, *Adora1*, and *Cnr1*, without eliciting widespread transcriptional remodeling. Collectively, these findings identify CBD as a well-tolerated anticonvulsant in the GASH/Sal model and suggest that pharmacokinetic exposure and the sex-specific molecular organization of the epileptogenic focus contribute to variability in treatment response, highlighting the importance of considering biological sex in cannabinoid-based epilepsy research.

## 1 Introduction

Epilepsy is a common and chronic neurological disorder that affects over 50 million people globally, leading to a significant public health burden (World Health Organization, 2022). It is defined by a persistent predisposition to generate seizures, which arise from abnormal, excessive, or synchronous neuronal activity within specific brain networks, which can remain focal or spread to involve the entire brain (Faingold, 2004; Fisher et al., 2014). Seizures are the hallmark and most debilitating symptoms of epilepsy, often serving as the primary clinical manifestation, while the condition is also frequently accompanied by a wide range of cognitive, behavioral, and psychiatric comorbidities that further compromise patients’ quality of life (Kanner et al., 2012; Scheffer et al., 2017). Although more than 30 antiseizure medications with diverse mechanisms of action and tolerability profiles are currently available, around 30%–40% of individuals with epilepsy remain pharmacoresistant. This means seizures persist despite adequate trials of at least two appropriate antiseizure medications, either as monotherapy or in combination (Kwan et al., 2009; Sharma et al., 2015). Pharmacoresistant epilepsy is associated with greater morbidity, a markedly increased risk of mortality—including sudden unexpected death in epilepsy—and substantial psychosocial consequences (Fattorusso et al., 2021). These challenges highlight the urgent need for novel, more effective therapeutic strategies that can address drug-resistant seizures.

Preclinical models have played a vital role in shaping our understanding of epilepsy and guiding the development of new antiseizure therapies. Among these, genetically susceptible animal models are especially valuable because they naturally exhibit seizures, providing a reliable and consistent platform for studying disease mechanisms and testing drug efficacy. Reflex epilepsy models—such as those involving audiogenic seizure (AGS)—stand out for their ability to consistently provoke seizures in response to sound, without the need for invasive chemical or electrical induction. This not only helps preserve the integrity of brain tissue but also reduces variability in experimental outcomes, making them a powerful tool in epilepsy research (Kandratavicius et al., 2014; Garbuz et al., 2022). The Genetic Audiogenic Seizure Hamster from Salamanca (GASH/Sal) is a well-established AGS model that exhibits generalized tonic-clonic seizures in response to loud sound stimulation. These seizures originate primarily in the inferior colliculus, a midbrain auditory structure that acts as the epileptogenic focus in this model (Muñoz et al., 2017). The neurobehavioral and neurophysiological features observed in GASH/Sal seizures closely parallel those seen in generalized seizure types in other rodent models and humans (Carballosa-Gonzalez et al., 2013). GASH/Sal animals have been extensively characterized in terms of their behavioral, molecular, electroencephalographic and anatomical seizure phenotypes (e.g., in Barrera-Bailón et al., 2017; Díaz-Casado et al., 2020; Carballosa-Gonzalez et al., 2013; Sánchez-Benito et al., 2017; 2020). Previous studies have shown that both classical antiseizure medications such as valproic acid and phenobarbital, as well as newer agents like lamotrigine and eslicarbazepine acetate, effectively suppress AGS in this model, validating its utility for preclinical drug screening and mechanistic studies (Barrera-Bailón et al., 2013; 2017; Werner and Coveñas, 2017; Gonçalves-Sánchez et al., 2024).

Cannabidiol (CBD), the principal non-psychoactive phytocannabinoid of *Cannabis sativa*, has gained significant attention in recent years for its broad-spectrum therapeutic potential, particularly in epilepsy. A growing body of evidence from preclinical studies and randomized clinical trials—especially in patients with Dravet syndrome and Lennox-Gastaut syndrome—demonstrates that CBD can significantly reduce seizure frequency and severity while maintaining a generally favorable safety profile (Devinsky et al., 2017; Thiele et al., 2018). These findings led to the regulatory approval of Epidiolex®, a purified CBD oral solution, by the U.S. Food and Drug Administration (FDA) in 2018 and the European Medicines Agency (EMA) in 2019. However, despite these advances, CBD’s mechanisms of action remain incompletely understood, and its effects in preclinical epilepsy models—particularly those based on AGS—are still underexplored, with existing studies reporting inconsistent efficacy (Lazarini-Lopes et al., 2023b).

In our previous study, we evaluated the acute anticonvulsant effects of a 100 mg/kg intraperitoneal dose of CBD in male GASH/Sal hamsters and observed modest reductions in seizure severity (Cabral-Pereira et al., 2021). However, this benefit was not sustained with chronic administration, as the animals continued to develop severe tonic-clonic seizures. Despite these limitations, no significant changes were observed in biochemical markers of liver function or hematological parameters, indicating good tolerability at this dose (Cabral-Pereira et al., 2021). While these findings provided preliminary insight into CBD’s therapeutic potential, important questions remained regarding potential sex-related differences in efficacy and safety, as well as the impact of higher dosing on seizure control. These gaps are particularly relevant given increasing recognition of sex as a critical variable in pharmacological research (Clayton and Collins, 2014; Shansky and Woolley, 2016; Madla et al., 2021), and the dose-dependent nature of cannabinoid pharmacodynamics. Accordingly, the present study extends our previous work by testing a higher CBD dose (200 mg/kg) and, for the first time, evaluating its effects in both male and female GASH/Sal animals under acute and chronic treatment conditions. Our investigation included a comprehensive assessment of seizure severity, pharmacokinetic parameters, hematological and hepatic safety profiles, as well as body weight monitoring. To explore potential molecular mechanisms, we also analyzed gene expression in the inferior colliculus. Specifically, we focused on six targets implicated in excitability and neuroprotection: transient receptor potential vanilloid 1 (*Trpv1*), 5-hydroxytryptamine receptor 1A (*5-Htr1a*), sigma non-opioid intracellular receptor 1 (*Sigmar1*), adenosine A1 receptor (*Adora1*), equilibrative nucleoside transporter 1 (*Slc29a1*), and cannabinoid receptor 1 (*Cnr1*). These genes, which regulate synaptic transmission and neuronal activity, were chosen because of their established relevance to CBD’s pharmacological profile and their potential role in seizure modulation (Franco and Perucca, 2019; Yang et al., 2020). This integrative, sex-aware approach seeks to clarify the therapeutic profile of CBD in AGS models and contributes to broader efforts to refine precision-based strategies in epilepsy research.

## 2 Materials and methods

### 2.1 Experimental animals and ethical statement

A total of 72 GASH/Sal hamsters (24 males and 48 females) from the inbred colony maintained at the vivarium of the University of Salamanca (USAL, Spain) were used in this study. Animals were 4 months old, an age at which the GASH/Sal strain exhibits peak susceptibility to AGS, with near-complete penetrance of seizure expression, as previously established (Muñoz et al., 2017). All experimental procedures were conducted in accordance with the European Directive 2010/63/EU on the protection of animals used for scientific purposes and were approved by the Bioethics Committee of the University of Salamanca (project number 380). Animals were housed under standard laboratory conditions in Eurostandard Type III cages (Tecniplast, Italy) with Lignocel bedding (Rettenmaier Ibérica), maintained on a 14:10 h light/dark cycle at 22 °C–24 °C, with *ad libitum* access to food (Teklad Global 2,918 irradiated diet) and water. Animals were initially maintained in groups of 3–4 per cage and individually housed 24 h before drug administration to ensure consistent treatment conditions. All handling and injections were performed by the same researcher to minimize stress and reduce experimental variability. In addition, all experiments were conducted at the same time of day to minimize the influence of circadian rhythms. This study adhered to the 3Rs principles—Replacement, Reduction, and Refinement—by using the fewest animals necessary to achieve statistically meaningful results, maximizing data obtained per animal, and applying refined methods to minimize pain, distress, and suffering in accordance with current welfare standards and *in vivo* research practices.

### 2.2 Experimental design and drug administration

**FIGURE 1:**
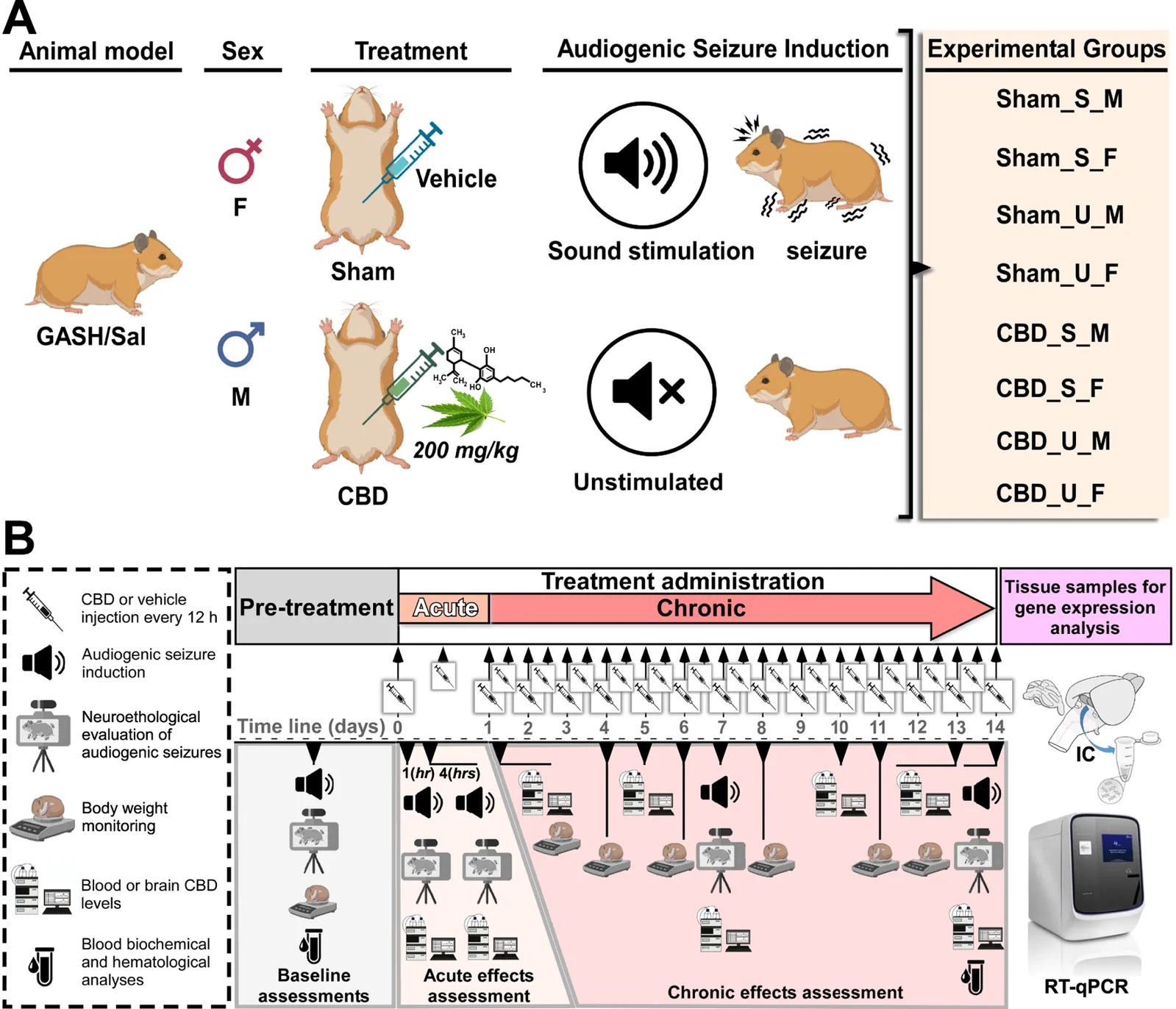
Experimental design and timeline. **(A)** Schematic of the 2 × 2 × 2 factorial design used to evaluate the effects of treatment (CBD or vehicle), acoustic stimulation (stimulated or unstimulated), and sex (male or female), resulting in eight experimental groups: Sham_S_M (vehicle, stimulated, male), Sham_S_F (vehicle, stimulated, female), Sham_U_M (vehicle, unstimulated, male), Sham_U_F (vehicle, unstimulated, female), CBD_S_M (CBD, stimulated, male), CBD_S_F (CBD, stimulated, female), CBD_U_M (CBD, unstimulated, male), and CBD_U_F (CBD, unstimulated, female). **(B)** Timeline of the 14-day experimental protocol. Baseline assessments included body weight, hematological and biochemical profiles, and seizure screening. Animals were treated with CBD (200 mg/kg) or vehicle every 12 h for 14 days. Seizure severity, convulsive phase duration, and neuroethological parameters were assessed 1 and 4 h after the first dose (acute phase), and on days 7 and 14 (chronic phase). Body weight was monitored throughout. On day 14, blood was collected for hepatic and hematological analysis. Animals were euthanized 1 h after the final seizure induction; the inferior colliculus (IC) was dissected for gene expression analysis, and one cerebral hemisphere was collected for CBD quantification. Additional pharmacokinetic studies (not shown) established the 1 h post-dose peak for brain and serum CBD levels.

The experimental design, adapted from Cabral-Pereira et al. (2021), is illustrated in Figure 1. Fifty animals (24 males and 26 females) were assigned to eight experimental groups (n = 4–8 per group) following a 2 × 2 × 2 factorial design to evaluate the effects of treatment (vehicle or CBD), seizure induction by acoustic stimulation (stimulated or non-stimulated), and sex (male or female). All treatments—vehicle (Cremophor® RH-based) and CBD—were administered via intraperitoneal injections under brief, light isoflurane anesthesia (4% isoflurane with 1 L/min O_2_). This approach, in accordance with refinement principles, was used to minimize handling stress and potential discomfort associated with repeated dosing. Sham animals, defined as those receiving only the vehicle, served as controls for distinguishing drug-specific effects. This factorial structure enabled a comprehensive analysis of CBD’s anticonvulsant and molecular effects, while accounting for both sex- and seizure-related variability. The eight experimental groups were defined by the combination of variables (see Figure 1A): vehicle-treated, stimulated animals were assigned to Sham_S_M (males; n = 6) and Sham_S_F (females; n = 6); unstimulated vehicle-treated animals to Sham_U_M (males; n = 4) and Sham_U_F (females; n = 6); CBD-treated, stimulated animals to CBD_S_M (males; n = 8) and CBD_S_F (females; n = 8); and non-stimulated CBD-treated animals to CBD_U_M (males; n = 6) and CBD_U_F (females; n = 6). Before initiating treatments, all animals underwent baseline assessments, including body weight monitoring, hematological and biochemical profiling, and a seizure induction protocol to confirm the presence of a complete seizure with all characteristic phases (Figure 1B).

Purified cannabidiol (CBD, >99% purity) extracted from *Cannabis sativa* was generously provided for research purposes by MJardin Group, Inc. (Toronto, Ontario, Canada) and RiverForce Partners, Inc. (Boston, MA, USA). CBD was suspended in a vehicle containing Cremophor® RH 40/ethanol/saline (1:2:17) and freshly prepared immediately before each injection. The selected dose of 200 mg/kg was guided by prior pharmacokinetic findings from our previous study, in which male GASH/Sal animals received 100 mg/kg and demonstrated measurable CBD concentrations in serum (Cabral-Pereira et al., 2021). To achieve higher and more sustained systemic exposure—consistent with anticonvulsant doses reported in preclinical models (Devinsky et al., 2014; Ibeas Bih et al., 2015)—an additional pharmacokinetic evaluation was performed in a separate cohort of 22 animals not included in the behavioral experiments and not exposed to acoustic stimulation. This follow-up investigation employed a multi-tiered approach, comprising three separate experimental sets to comprehensively evaluate both peripheral (serum) and central (brain) exposure to CBD. First, acute systemic exposure was assessed by measuring serum CBD concentrations in eight non-stimulated female GASH/Sal animals following a single intraperitoneal injection of 200 mg/kg, with blood samples collected at 15-, 30-, 45-, 60-, 120-, 240-, 480-, and 720-min post-injection. Second, to evaluate drug accumulation during chronic treatment, 6 additional non-stimulated females received CBD twice daily (every 12 h), with blood samples collected on treatment days 1, 5, 10, and 13 at three time points: pre-dose, and 1-, 4-, and 8-h post-injection. Males were not included in this pharmacokinetic evaluation in accordance with the principle of reduction, as the previously established male pharmacokinetic profile provided a reference framework (Cabral-Pereira et al., 2021) and did not justify duplicating a full pharmacokinetic assessment at the higher dose used here. Critically, measuring drug levels in brain tissue is essential to verify that the compound crosses the blood–brain barrier and reaches its intended targets in the central nervous system (de Lange, 2013); therefore, brain CBD concentrations were measured in a third experimental set. This involved a separate cohort of female animals sacrificed at 1 h (n = 4) and 4 h (n = 4) following CBD administration to evaluate direct brain exposure. These animals were euthanized following the procedures previously described for the GASH/Sal model (Barrera-Bailón et al., 2017). Brains were rapidly removed, dissected, snap-frozen in liquid nitrogen, and stored at −80 °C until analysis. CBD quantification was performed using high-performance liquid chromatography coupled to tandem mass spectrometry (HPLC–MS/MS), as detailed below.

Based on the integrated serum and brain concentration data (see below in the results section, Figure 3), the 4-h post-injection time point was identified as optimal for evaluating both acute and chronic anticonvulsant effects, as it aligned with peak CBD levels in both systemic circulation and brain tissue. Accordingly, animals received CBD (200 mg/kg) via intraperitoneal injection every 12 h for 14 days. Anticonvulsant efficacy was assessed by measuring seizure severity, convulsive phase durations, and conducting neuroethological analyses across both treatment phases. Seizure assessments were conducted at 1 and 4 h after the initial dose to evaluate acute effects, and again on treatment days 7 and 14—4 h after the first intraperitoneal injection of the day (administered at 7:30 a.m.)—to assess chronic effects (Figure 1B). Throughout the treatment period, body weight was monitored regularly to evaluate general health and identify potential adverse effects of repeated CBD administration (Figure 1B). On day 14, blood samples were collected for hematological and hepatic analyses, and results were compared with baseline pre-treatment values (Figure 1B). Following the final seizure induction, animals were euthanized 1 hour later, after which the brain was rapidly removed as described by Barrera-Bailón et al., 2017. One of the two cerebral hemispheres from each animal was harvested to quantify CBD concentrations in brain tissue following chronic treatment. In addition, the inferior colliculus—the primary epileptogenic nucleus in this model—was dissected for gene expression analyses of molecular targets associated with CBD pharmacodynamics and seizure-related pathways (Figure 1B). For both procedures, brain tissue samples were snap-frozen in liquid nitrogen immediately after collection and stored at −80 °C until analysis.

### 2.3 Sample collection, pharmacokinetics, and physiological monitoring

Blood sample collection and preparation were conducted according to previously described protocols (Barrera-Bailón et al., 2017; Cabral-Pereira et al., 2021), with modifications appropriate to the present study design. As detailed in the experimental design, samples were collected at protocol-defined time points depending on the intended analysis (Figure 1B). All blood was obtained from the cranial vena cava under inhalation anesthesia (induction: 4% isoflurane with 1 L/min O_2_; maintenance: 3% isoflurane with 0.4 L/min O_2_) using sterile, heparin-free syringes. Immediately after collection, animals received subcutaneous isotonic saline (0.9% NaCl) in a volume equivalent to the blood extracted to maintain fluid balance and prevent hypovolemia.

For CBD serum concentration analysis, 200 μL of whole blood was collected and allowed to clot at room temperature for 1 h. Samples were then centrifuged at 1,500 × g for 20 min at 4 °C using a Sorvall Legend Micro 21R centrifuge (Thermo Scientific). The resulting serum was aliquoted (100 μL) and stored at −80 °C until analysis. Serum CBD levels were quantified via HPLC-MS/MS at the Mass Spectrometry Service of the University of Salamanca. The analytical system consisted of a Waters Acquity UPLC HT® (Waters Corp., Milford, MA, USA) equipped with a binary pump and thermostatted column compartment, connected to a Waters Xevo TQ-S micro triple quadrupole MS. Chromatographic separation was achieved using an Acquity UPLC BEH C18 column (2.1 × 50 mm, 1.7 μm particle size) with a mobile phase composed of 0.1% formic acid in water (A) and acetonitrile (B) at a flow rate of 0.7 mL/min. A gradient elution program was applied as follows: 95% A at 0.00 min, 90% A at 0.35 min, 75% A at 0.99 min, 0% A from 1.00 to 1.90 min, and re-equilibration at 95% A by 1.91 min. To assess CBD levels in brain tissue samples, we adapted the extraction protocol for antiseizure medications described by Barrera-Bailón et al. (2017). Prior to extraction, snap-frozen brain tissue samples were retrieved from −80 °C storage. Briefly, the samples were homogenized and extracted with dichloromethane, selected for its high extraction efficiency for lipophilic cannabinoids. The resulting suspension was centrifuged at 13,000 × g for 15 min, and the organic phase was collected, evaporated to dryness under nitrogen stream, and reconstituted in 1 mL of a 1:1 (v/v) acetonitrile–water mixture. An aliquot of this reconstituted solution was analyzed, and CBD concentration was subsequently determined using the same HPLC–MS/MS conditions described above for serum analysis.

For hematological analysis, 500 μL of blood was collected into K_3_-EDTA-coated tubes and immediately processed using an automated hematology analyzer (ADVIA® 120, Bayer, Germany) to assess hemoglobin concentration, hematocrit, and counts of erythrocytes, leukocytes, and platelets.

For biochemical assessments, 400 μL of blood was collected into anticoagulant-free tubes and allowed to clot for 30 min at room temperature. Samples were centrifuged at 1,500 × g for 10 min at 4 °C, and the resulting serum was stored at −20 °C until analysis. Biochemical markers of liver function—including aspartate aminotransferase (AST), alanine aminotransferase (ALT), total bilirubin, serum albumin, and total protein—were quantified using commercial kits (Spotchem II Liver-1 kit #33925, Menarini Diagnostics) and analyzed with a Spotchem EZ automated analyzer (SP-4430) according to the manufacturer’s instructions.

Body weight was recorded prior to the initiation of treatment and subsequently monitored three times per week for all experimental groups throughout the study duration (Figure 1B). Regular monitoring of body weight is a standard and sensitive indicator of general health status, nutritional balance, and potential systemic toxicity in pharmacological research (Talbot et al., 2020). Together, these measures provided a comprehensive evaluation of potential adverse effects associated with repeated CBD administration.

### 2.4 Audiogenic seizure induction and neuroethological analysis

AGS were induced in GASH/Sal animals via intense acoustic stimulation, following previously validated protocols (Barrera-Bailón et al., 2013; Cabral-Pereira et al., 2021). To evaluate treatment effects, seizures were induced 1 and 4 h after a single CBD or vehicle injection for the acute phase, and 4 h after the first intraperitoneal dose on days 7 and 14 for the chronic phase (Figure 1B). Each animal was placed in a cylindrical acrylic arena (50 cm height × 37 cm diameter) and allowed to acclimate for 1 min. Seizures were triggered by continuous white noise (0.5–18 kHz, 115–120 dB SPL) delivered from a speaker (Beyma T2010, Valencia, Spain) mounted above the arena and powered by an amplifier (Fonestar MA-25T, Revilla de Camargo, Spain). The sound was filtered above 500 Hz (Bruel & Kjaer microphone #4134 and preamplifier #2619), digitized at 44.1 kHz, and generated by a computer. Acoustic stimulation was terminated upon initiation of the wild running phase or after 20 s, whichever occurred first.

**FIGURE 2:**
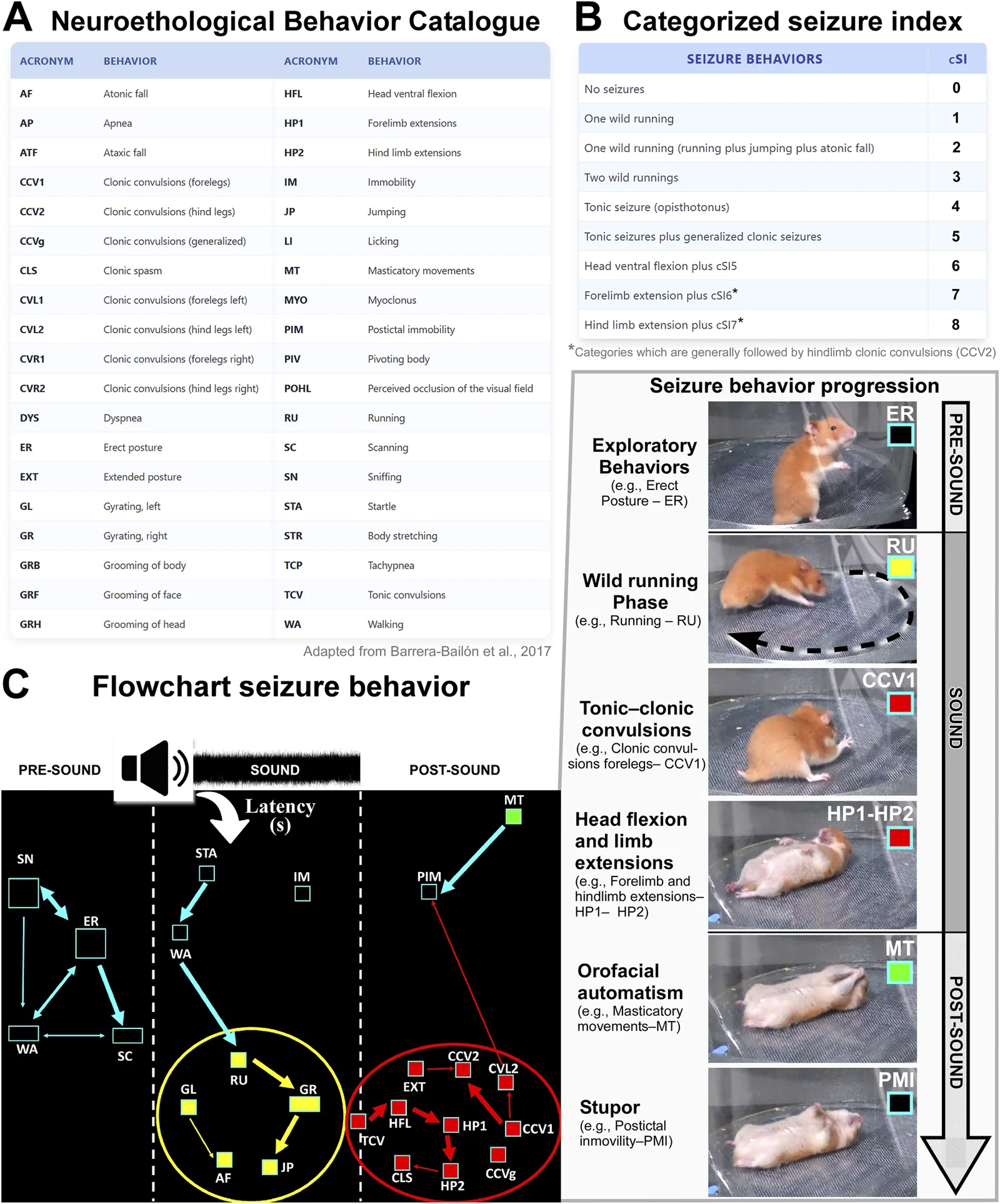
Audiogenic seizure analysis in the GASH/Sal model. **(A)** Table summarizing the abbreviations and definitions of the behavioral variables analyzed in the neuroethological study, which were used to generate flowcharts of behavioral sequences. **(B)** Table displaying the categorized seizure index (cSI), a measure of seizure severity in which observed behaviors are quantified and classified into discrete levels ranging from 0 (no seizures) to 8 (complete seizures), to facilitate statistical analysis. **(C)** Representative behavioral flowchart illustrating the temporal organization and statistical relationships among seizure-related behaviors. The height and width of each rectangle correspond to the frequency and total duration of the behavior, respectively. Arrow width indicates the statistical strength of transitions between behaviors, and arrow direction shows the preferred sequence. Behaviors related to the wild running phase (e.g., wild running, jumping, falling) are shown in yellow; behaviors characteristic of the convulsive phase (e.g., clonic seizures, limb extension) are shown in red, as previously described (Barrera-Bailón et al., 2013). Behavioral observations were analyzed within three temporal windows: pre-sound (before auditory stimulation), sound (during seizure-inducing auditory stimulation), and post-sound (after stimulation). Representative behavioral sequences recorded during these periods, assessed using neuroethological methods, are shown in the right panel.

Video recordings began 1 min before the onset of acoustic stimulation and continued until the animal recovered from post-ictal stupor. To analyze seizure behavior in detail, we employed the neuroethological approach using the ETHOMATIC software (Garcia-Cairasco et al., 1992), which has been previously applied to characterize seizure phenotypes in GASH/Sal animals (e.g., Cabral-Pereira et al., 2021; Gonçalves-Sánchez et al., 2024) as well as in other AGS models and humans (e.g., Garcia-Cairasco et al., 2004; Dal-Cól et al., 2006). Behaviors were annotated second by second based on a predefined ethogram of behavioral items (Figure 2A), originally described by Garcia-Cairasco et al. (1992). Seizure severity was scored using a categorized seizure index (cSI) based on observed behaviors, as illustrated in Figure 2B (Garcia-Cairasco et al., 2004; Barrera-Bailón et al., 2013; Barrera-Bailón et al., 2017). Based on these cSI scores, animals were classified as seizure-susceptible (score ≥2) or minimally affected (score <2). Seizure latency was defined as the time interval between the onset of acoustic stimulation and the initiation of the wild running phase. Behavioral analysis was conducted across three distinct temporal windows: pre-sound (1 min before stimulation), sound (first 30 s of stimulation), and post-sound (1 min after stimulation) (Figure 2C). The ETHOMATIC software calculated the frequency and duration of each behavior and performed Chi-square tests to identify statistically significant transitions between behavioral items. These relationships were visualized as behavioral flowcharts, in which the size of each rectangle represents the frequency and duration of a behavior, while arrow direction and width indicate the sequence and strength of behavioral associations (Figure 2C). Flowcharts were generated by the ETHOMATIC software and displayed using Microsoft PowerPoint 365 (Microsoft Corp., Redmond, WA, USA).

### 2.5 Gene expression analysis (quantitative real-time PCR)

To investigate the molecular effects of CBD, we analyzed the expression of selected genes in the inferior colliculus of GASH/Sal animals. As described above, at the end of the treatment period (day 14), the inferior colliculus was dissected immediately after euthanasia, snap-frozen in liquid nitrogen, and stored at −80 °C until analysis. The selected genes—*Trpv1, 5-Htr1a, Sigmar1, Adora1, Slc29a1,* and *Cnr1*—encode receptors and transporters involved in neuroprotection and the regulation of neuronal excitability and have been implicated in the pharmacological actions of CBD (Franco and Perucca, 2019; Yang et al., 2020). RNA extraction and cDNA synthesis were performed following protocols routinely used in our laboratory (López-López et al., 2017; Damasceno et al., 2020; Díaz-Rodríguez et al., 2024). Briefly, 2 μg of total RNA from each sample was reverse-transcribed into cDNA using oligo-dT and random hexamer primers with the First Strand cDNA Synthesis Kit (K1621, Thermo Fisher Scientific, MA, USA). Reverse transcriptase-negative controls were included to confirm the absence of genomic DNA contamination. Quantitative real-time PCR (RT-qPCR) was conducted using SYBR Green chemistry (2× Master Mix, #4367659, Applied Biosystems) on a QuantStudio 7 Flex Real-Time PCR System (Applied Biosystems). Each 20 μL reaction consisted of 10 μL of Master Mix, 0.4 μL of each gene-specific primer, 3 μL of serially diluted cDNA (optimized for quantification), and nuclease-free water. The thermal cycling protocol was: initial denaturation at 95 °C for 10 min, followed by 40 cycles of 95 °C for 15 s and 60 °C for 30 s (annealing/extension). Reactions were run in triplicate using 4–7 biological replicates per group. Primer sequences are provided in Supplementary Material 1. PCR efficiency was validated using standard curves generated from cDNA serial dilutions (60–1.65 ng/μL), confirming high target gene expression and strong linearity (R^2^ > 0.95). Amplification efficiency (E) was calculated using the formula: E = [10^(–1/slope)^ – 1] × 100. Similar slopes across all genes indicated consistent amplification efficiency within the tested range. *Actb* (β-actin) was used as the reference gene for normalization of target gene expression. This choice was based on its prior validation and consistent use in the GASH/Sal model under similar experimental conditions (e.g., in Díaz-Rodríguez et al., 2020; López-López et al., 2017; Zeballos et al., 2025), ensuring methodological continuity and enabling comparison with previously published datasets. Negative template (no-cDNA) controls were included in all RT-qPCR runs. Relative gene expression levels were determined using the ΔCt method, in which the cycle threshold (Ct) value of each target gene was normalized to that of the reference gene (ΔCt = Ct_target_–Ct_reference_), following the method of Livak and Schmittgen (2001). The target Ct value was calculated as the arithmetic mean of all technical replicates. After outlier exclusion (Burns et al., 2005), the resulting ΔCt values served as the primary measure of relative mRNA abundance for all subsequent statistical analyses. To facilitate the exploration and presentation of gene expression data, ΔCt values were further standardized within each gene to scaled ΔCt (z-score), calculated as (ΔCt_sample_ − mean ΔCt_gene_)/SD ΔCt_gene_). Since lower ΔCt indicate higher transcript abundance in qPCR assays, z-scores were sign-inverted for visualization such that higher values correspond to higher relative expression and lower values to lower relative expression. Static heatmap and bar plots visualizations were generated in GraphPad Prism (version 10.2.3; GraphPad Software, San Diego, CA, USA). In addition, an interactive data visualization tool was custom-developed as a self-contained HTML5 application to enable dynamic exploration of the complete dataset, including heatmaps, bar charts, and grouped summaries displaying group means ± SD. The application was implemented using native HTML5, Cascading Style Sheets (CSS3), and JavaScript (ECMAScript, 2020) without external libraries or server-side dependencies, thereby ensuring full reproducibility, and cross-platform compatibility across modern web browsers. Development was assisted by the artificial intelligence platform Claude Opus (Anthropic, San Francisco, CA, USA); all quantitative outputs, and biological interpretations incorporated in the application were independently verified by all authors prior to submission. This interactive tool is provided as Supplementary Material 4 to accompany this article.

### 2.6 Statistical analysis

For simple two-group comparisons of pharmacokinetic data and baseline body weight between treatment groups within each sex, independent samples Student's t-tests were applied. Prior to parametric testing, data were assessed for normality and homogeneity of variances using the Shapiro–Wilk and Levene’s tests, respectively. If assumptions were violated, data were transformed or non-parametric alternatives (e.g., Mann–Whitney U test) were considered. All statistical tests were conducted using GraphPad Prism, version 10.2.3 (GraphPad Software Inc., San Diego, CA, USA) and quantitative data were expressed as mean ± standard error of the mean (SEM), except for seizure severity analysis.

To evaluate the longitudinal effects of treatment on seizure severity (cSI) while accounting for biological sex, cSI was rated on an ordinal scale ranging from 0 (no seizure activity) to 8 (maximal, fully generalized seizure) and was assessed repeatedly in the same animals at five time points (pre-treatment baseline, and 1 h, 4 h, 7 days and 14 days after treatment). The experiment followed a 2 × 2 × 5 mixed factorial design, with treatment (CBD vs. sham) and sex (male vs. female) as between-subject factors and time as a within-subject, repeated-measures factor. Because the severity index is ordinal, parametric analysis of variance on the raw scores was inappropriate; the natural ordinal alternative—a cumulative-link (ordinal) mixed-effects model—could not be identifiably fitted because sham-treated control animals remained uniformly at the ceiling of the scale, producing quasi-complete separation. Rank-based, non-parametric methods were therefore used for all analysis. The global effects of treatment, sex and time, together with their interactions, were evaluated using the non-parametric analysis of longitudinal data in factorial experiments developed by Brunner and colleagues (Brunner et al., 2002), applying an F2-LD-F1 design (two whole-plot/between-subject factors [treatment and sex] and one sub-plot/within-subject factor [time]), as implemented in the nparLD package (Noguchi et al., 2012). ANOVA-type statistics (ATS), with their estimated degrees of freedom and corresponding P values, are reported for each main effect and interaction. Pre-specified comparisons were then performed to localize the effects. Because neither the treatment × sex interaction nor any male–female comparison was significant, the sexes were pooled for the cannabidiol-versus-sham and within-cannabidiol baseline comparisons (CBD, n = 16; sham, n = 12). Differences between treatments (CBD vs. sham, sexes pooled) at each time point, and differences between sexes within the CBD group at each time point (n = 8 per sex), were assessed with the Mann–Whitney U (Wilcoxon rank-sum) test. Within the CBD group (sexes pooled), the overall effect of time was tested with the Friedman test, and each post-treatment time point was compared with the animal’s own pre-treatment baseline using the Wilcoxon signed-rank test. Owing to the presence of tied values, the asymptotic (normal-approximation) versions of these tests were used. To control the family-wise error rate, P values were adjusted with the Holm method within each family of comparisons. Effect sizes are reported as the rank-biserial correlation coefficient for the between-group and between-sex (Mann–Whitney) comparisons; for the within-CBD baseline comparisons, the median change in cSI is reported. Severity data are summarized as the median and interquartile range (IQR). Analyses for seizure severity were carried out in R version 4.3.3 (R Core Team, 2024) using the nparLD package (v2.2) and base-R functions for the rank-based tests.

Seizure latency and the durations of the wild running and convulsive phases were analyzed separately using mixed-effects models (restricted maximum likelihood, REML). A mixed-effects approach was used for these variables because animals exhibiting complete seizure suppression (cSI = 0) cannot yield latency or phase-duration values and were excluded; as the number of such animals increased over the treatment period—particularly in CBD-treated females—these datasets were unbalanced and contained missing observations across timepoints, conditions under which mixed-effects models accommodate the data without the listwise deletion required by repeated-measures ANOVA. Separate models were fitted for each pre-specified pairwise comparison of interest—Sham vs. CBD within males, Sham vs. CBD within females, and males vs. females within each treatment group (Sham males vs. Sham females, and CBD-treated males vs. CBD-treated females)—each including the corresponding grouping factor (treatment or sex) as a between-subject factor and time as the within-subject repeated factor. Sphericity was not assumed, and the Geisser–Greenhouse correction was applied. When a significant effect or interaction was detected, groups were compared at each timepoint using Šídák’s multiple comparisons test.

Associations between seizure severity and CBD concentrations in serum and brain tissue were explored using Spearman’s rank correlation analysis, given the non-parametric distribution and potential variability of the data.

Gene expression data (ΔCt values) were analyzed using three-way ordinary analysis of variance (ANOVA), with sex (male vs. female), treatment (CBD vs. vehicle), and stimulation condition (unstimulated vs. acoustically stimulated) as between-subject factors. For each gene, the model evaluated all main effects, two-way interactions (Sex × Treatment, Sex × Condition, and Treatment × Condition), and the three-way Sex × Treatment × Condition interaction. The percentage of total variation accounted for by each main effect and interaction term was calculated from the ANOVA output as an index of relative effect size. Longitudinal changes in body weight were analyzed using a mixed-effects model (REML), with treatment and sex as between-subject fixed factors and time as the within-subject repeated measure. Hematological parameters and serum biochemical variables were analyzed using two-way ANOVA with treatment and sex as between-subject factors. For the seizure severity, gene expression, body weight, hematological, and biochemical analyses, *post hoc* comparisons following a significant effect or interaction were conducted using Tukey’s honestly significant difference (HSD) test; for the seizure latency and phase-duration analyses, the pre-specified comparisons were evaluated using Šídák’s multiple comparisons test (above). Both methods control the family-wise type I error rate. All tests were two-tailed, and statistical significance was defined at *p < 0.05* (*), *p < 0.01* (**), *p < 0.001* (***), and *p < 0.0001* (****). Outliers were identified and excluded only when justified according to the criteria described by Burns et al. (2005).

## 3 Results

### 3.1 Pharmacokinetics of CBD in serum and brain tissue following acute and chronic administration

**FIGURE 3:**
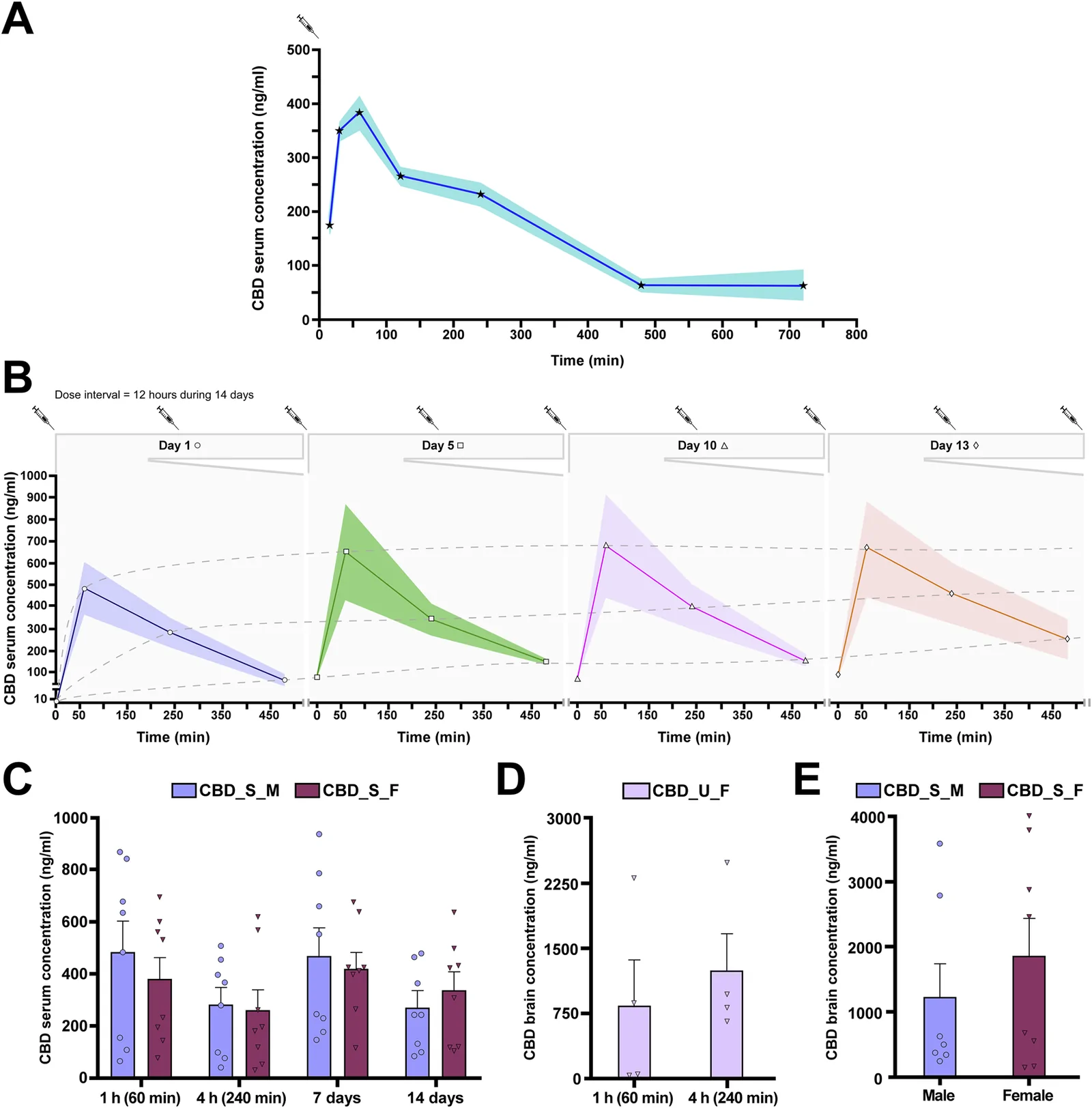
CBD concentrations in serum and brain tissue following acute and chronic administration. **(A)** Time course of serum CBD concentrations in non-stimulated female GASH/Sal animals (n = 8) following a single intraperitoneal injection of CBD (200 mg/kg). Peak levels were detected within 1-h post-administration. **(B)** Serum CBD concentrations in non-stimulated females (n = 6) during chronic treatment with CBD (200 mg/kg, every 12 h for 14 days). Pharmacokinetic profiles are shown in different colors for treatment days 1, 5, 10, and 13. A progressive rise in peak serum concentrations was observed across time points, stabilizing into a plateau (indicated by dashed lines). **(C)** Serum CBD concentrations in male (n = 8) and female (n = 8) animals exposed to audiogenic seizure induction protocols at the endpoint of the acute (1 and 4 h) and chronic (days 7 and 14) treatment phases. No significant sex-related differences were detected. **(D)** CBD concentrations in brain tissue of non-stimulated female animals at 1 h (n = 4) and 4 h (n = 4) following a single CBD dose (200 mg/kg), indicating measurable central exposure with notable between-subject variability. **(E)** Brain CBD concentrations in acoustically stimulated male (n = 8) and female (n = 8) animals at the end of chronic treatment. No significant sex-related differences were detected, although females exhibited higher mean concentrations. Note that considerable inter-individual variability was observed across animals. For all two-group comparisons (panels C, D, and E), unpaired Student's t-tests were applied. Statistical significance was set at *p < 0.05*. Values represent mean ± SEM.

To determine the pharmacokinetic profile of CBD in the GASH/Sal model, serum and brain concentrations were measured after both acute and chronic intraperitoneal administration of 200 mg/kg CBD (Figure 3). Following a single injection in non-stimulated females, serum CBD levels peaked at 384.6 ± 23.3 ng/mL at 60 min post-administration, declining to 281.5 ± 12.5 ng/mL at 2 h and stabilizing around 232.8 ± 18.0 ng/mL at 4 h (Figure 3A). Concentrations further decreased to 89.9 ± 8.6 ng/mL at 8 h and 72.5 ± 21.3 ng/mL at 12 h. CBD was detectable in serum for up to 12 h but fell below the detection threshold at 24 h. These results indicated that the optimal time points for assessing CBD’s anticonvulsant effects are 1- and 4-h post-injection, corresponding to peak serum levels and a subsequent phase of sustained systemic exposure, respectively—thereby enabling evaluation of both the onset and persistence of CBD’s potential therapeutic action.

To evaluate systemic CBD accumulation over the course of chronic treatment, serum concentrations were measured in non-stimulated female animals receiving CBD (200 mg/kg) every 12 h for 14 days. Sampling was performed at defined time points on treatment days 1, 5, 10, and 13 (Figure 3B). On day 1, peak serum levels reached 484.9 ± 118.1 ng/mL at 1-h post-injection, declining to 281.1 ± 66.3 ng/mL at 4 h and 63.2 ± 26.8 ng/mL at 8 h. By day 5, pre-dose baseline levels increased to 83.5 ± 3.7 ng/mL, with a peak of 649.4 ± 214.9 ng/mL at 1 h, followed by a reduction to 341.0 ± 68.7 ng/mL at 4 h and 148.0 ± 9.3 ng/mL at 8 h. This pattern continued through day 10, where pre-dose concentrations were 69.9 ± 3.2 ng/mL, reaching 678.0 ± 229.8 ng/mL at 1 h, and declining to 399.3 ± 98.7 ng/mL and 127.9 ± 10.6 ng/mL at 4 and 8 h, respectively. On day 13, a pre-dose level of 80.0 ± 5.3 ng/mL was followed by a peak concentration of 661.9 ± 234.7 ng/mL at 1-h post-injection, with subsequent decreases to 456.9 ± 138.9 ng/mL at 4 h and 248.5 ± 90.1 ng/mL at 8 h. Collectively, these data suggest progressive accumulation of CBD with repeated twice-daily dosing, reaching a reproducible steady-state serum concentration profile by approximately day 5 of treatment, characterized by consistent peak–trough fluctuations across subsequent dosing days (Figure 3B).

No statistically significant sex-related differences were detected in serum CBD concentrations following either acute or chronic administration in animals subjected to AGS stimulation (Figure 3C). These analyses were performed in male and female GASH/Sal animals that completed the full chronic treatment protocol illustrated in Figure 1B, including repeated CBD administration and seizure testing. After a single intraperitoneal injection of 200 mg/kg CBD, both males and females exhibited comparable serum concentrations at 1 h (∼450 ng/mL), followed by similar declines at 4 h. These findings suggest equivalent initial absorption and systemic elimination kinetics across sexes. During chronic treatment, serum CBD levels measured 4 h post-dose on treatment days 7 and 14 remained largely similar between males and females. A modest, non-significant difference in serum concentrations between sexes was observed on day 14; however, the direction of this difference was not consistent across time points, indicating inter-individual variability rather than a systematic sex-related effect (Figure 3C).

Since serum concentrations alone do not confirm drug penetration into the brain, CBD levels were also quantified in brain tissue to assess central exposure. In non-stimulated female animals, acute administration of CBD (200 mg/kg) resulted in brain concentrations of 828.0 ± 352.8 ng/mL at 1 h and 1,235.4 ± 424.7 ng/mL at 4 h post-dose (Figure 3D), indicating measurable brain exposure within this time window, despite greater inter-individual variability at the earlier time point. Following 14 days of twice-daily CBD administration, brain concentrations measured at the end of treatment were 1,214.0 ± 524.4 ng/mL in males and 1835.1 ± 587.2 ng/mL in females (Figure 3E). Although mean concentrations were higher in females, this difference did not reach statistical significance. When considered in relation to the acute female condition, these values are consistent with accumulation of CBD in brain tissue during repeated dosing. Overall, these findings indicate that the dosing regimen used (200 mg/kg every 12 h) produces sustained systemic and brain CBD exposure across the treatment period, supporting the use of this dosing schedule for subsequent analyses of CBD effects on seizure activity in the GASH/Sal model.

### 3.2 CBD attenuates audiogenic seizure severity: correlation with serum and brain levels

**FIGURE 4:**
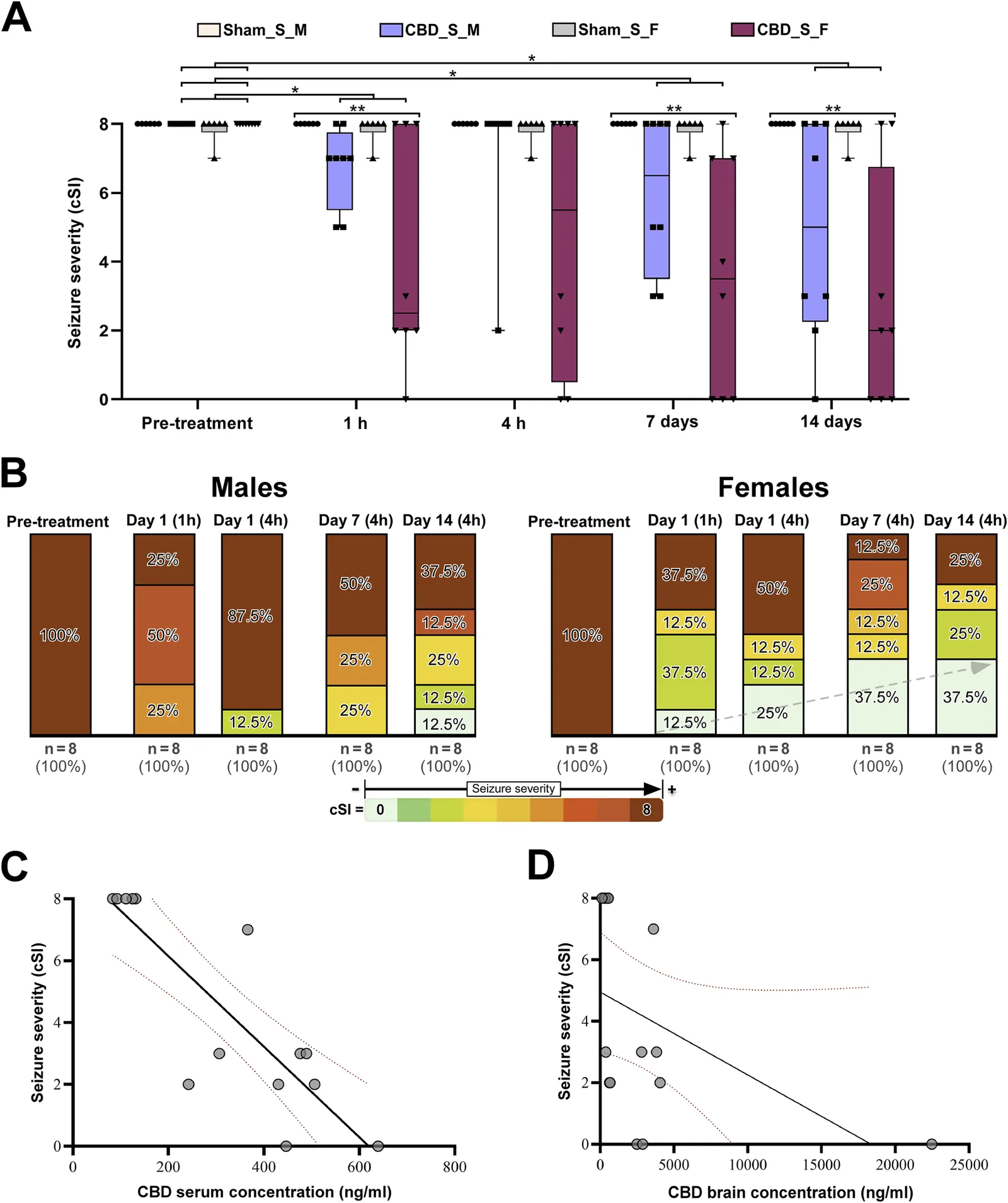
Effects of acute and chronic CBD treatment on seizure severity and its association with CBD exposure. **(A)** Seizure severity, quantified using the categorized seizure index (cSI; scale from 0 = no seizure to 8 = maximal generalized seizure), in sham- and CBD-treated GASH/Sal animals of both sexes at baseline (pre-treatment), during the acute phase (1 h and 4 h post-injection on day 1) and during the chronic phase (days 7 and 14, assessed 4 h post-injection). The same animals were assessed at every time point (n = 6 sham males, 8 CBD males, 6 sham females, 8 CBD females). Boxes show the median and interquartile range, whiskers the minimum and maximum, and individual animals are overlaid. All analyses were rank-based and non-parametric (full output and raw data in Supplementary Material 2). The main effects of treatment and time and the treatment × time interaction were all significant (*p* < 0.0001; nparLD; Supplementary Material 2, Panel B). Because the treatment × sex interaction and all male-versus-female comparisons were non-significant (Supplementary Material 2, Panels B and E), the sexes were pooled for the post-hoc comparisons and are displayed separately for clarity only. Brackets spanning each time point denote CBD versus sham (Mann–Whitney *U* test); brackets originating from pre-treatment denote CBD versus its own baseline (Wilcoxon signed-rank test). *P* values were Holm-adjusted and all tests were two-tailed; comparisons at pre-treatment and 4 h were non-significant and are unmarked. **(B)** Stacked bar plots showing the distribution (%) of CBD-treated animals across seizure severity categories in males (left) and females (right), at baseline and after acute (day 1, 1 h and 4 h) and chronic (days 7 and 14, 4 h post-injection) CBD administration. Vehicle-treated groups are not shown, as all Sham animals retained maximal seizure severity (cSI = 8) at every time point. The color scale (bottom) ranges from cSI = 0 (complete seizure suppression, light green) to cSI = 8 (maximal severity, dark brown). The dashed arrow highlights the progressive increase in the proportion of CBD-treated females achieving complete seizure suppression over time. **(C,D)** Spearman rank correlations between cSI scores and CBD concentrations measured in serum **(C)** and brain tissue **(D)** of CBD-treated animals at the end of the chronic treatment period (day 14). Solid lines represent linear regression overlays shown for visual reference only, whereas the reported *r* and *p* values were derived from Spearman rank correlation analyses. Dotted lines indicate the corresponding 95% confidence bands.

To evaluate the anticonvulsant effects of CBD, seizure severity was quantified across all experimental groups using the cSI (Figure 2A). Prior to treatment, GASH/Sal animals of both sexes exhibited uniformly maximal seizure severity (cSI = 8), with the sole exception of one female in the vehicle group that presented a marginally lower score (cSI = 7), confirming the consistency of the baseline audiogenic phenotype across groups (Figure 4A). Vehicle-treated animals retained maximal cSI scores throughout both the acute and chronic phases, attesting to the temporal stability of the untreated phenotype (Figure 4A).

Across the full 2 × 2 × 5 factorial design, non-parametric analysis of the longitudinal data (nparLD, F2-LD-F1) revealed significant main effects of treatment (ATS = 22.61, df = 1, *p* < 0.0001) and time (ATS = 8.78, df = 2.66, *p* < 0.0001) and a significant treatment × time interaction (ATS = 8.78, df = 2.66, *p* < 0.0001), indicating that the effect of CBD on seizure severity emerged and evolved over the course of the protocol (Figure 4A; Supplementary Material 2, Panel B). A significant main effect of sex was also detected (ATS = 4.15, df = 1, *p* = 0.042); however, neither the treatment × sex (*p* = 0.561) nor the treatment × sex × time (*p* = 0.446) interaction was significant, and male-versus-female comparisons within the CBD group did not differ at any time point (Supplementary Material 2, Panel E). Because the response to CBD did not depend significantly on sex, the sexes were pooled for all subsequent group comparisons.

At baseline, seizure severity was maximal and indistinguishable between groups (median cSI 8 [IQR 8–8] in all groups; CBD versus sham, *p* = 0.279), establishing comparable pre-treatment severity, while sham-treated animals remained at the ceiling of the scale throughout the 14-day protocol (Figure 4A; Supplementary Material 2, Panel A). Relative to sham, CBD significantly reduced cSI at 1 h (*W* = 34.5, *r* = −0.64; Holm-adjusted *p* = 0.0063), 7 days (*W* = 33.5, *r* = −0.65; *p* = 0.0063) and 14 days (*W* = 33.0, *r* = −0.66; *p* = 0.0063), with large effect sizes at all three time points; no significant difference was detected at 4 h (*p* = 0.241) (Figure 4A, lower brackets; Supplementary Material 2, Panel C).

The within-CBD time course mirrored these between-group findings. Seizure severity varied significantly across time within the CBD group (Friedman test, χ^2^(4) = 19.00, *p* < 0.001), and cSI was significantly lower than each animal’s own pre-treatment baseline at 1 h, 7 days and 14 days (Wilcoxon signed-rank test, Holm-adjusted *p* = 0.014 for all three) but not at 4 h (*p* = 0.057) (Figure 4A, upper brackets; Supplementary Material 2, Panel D). The magnitude of this within-animal reduction increased through the chronic phase, the median change in cSI from baseline reaching −1 at 1 h, −3 at 7 days and −5 at 14 days. Together, these analyses delineate a clear time-dependent profile: an acute anticonvulsant effect 1 h after administration, a transient return toward baseline at 4 h, and a progressively greater, sustained reduction in seizure severity during chronic treatment at 7 and 14 days.

Although the anticonvulsant response was numerically more pronounced in females than in males at every post-treatment time point — for example, at 14 days the median cSI was 2 [0–4.25] in CBD-treated females compared with 5 [2.75–8] in CBD-treated males (Supplementary Material 2, Panel A) — this apparent sex difference did not reach statistical significance in any direct male-versus-female comparison (Supplementary Material 2, Panel E) and was not accompanied by a significant treatment × sex interaction. The distribution of severity scores within the CBD group illustrates the same trend descriptively (Figure 4B). During the acute phase, the proportion of CBD-treated females achieving complete seizure suppression (cSI = 0) doubled from 12.5% at 1 h to 25% at 4 h, whereas no male fell below a cSI of 2 at either time point. The divergence widened under chronic dosing: at Day 7, 62.5% of females showed reduced severity — including 37.5% with complete suppression — while half of the males remained at maximal severity (cSI = 8) and none achieved suppression. By Day 14, 62.5% of females had low scores (cSI ≤ 2), with 37.5% maintaining complete suppression and only 25% retaining maximal severity, whereas among males 37.5% still scored at the ceiling and only 12.5% reached complete suppression. These proportions describe the same numerically greater female response and, in the absence of significant formal sex comparisons, should be interpreted as exploratory.

To examine the relationship between drug exposure and the observed anticonvulsant effect, Spearman rank correlations were computed between cSI values and CBD concentrations in serum and brain tissue at the end of the chronic period. Serum CBD concentrations were strongly and inversely correlated with seizure severity (*r* = −0.80, *p* = 0.0009), indicating that higher systemic exposure was associated with greater seizure attenuation (Figure 4C). A parallel inverse correlation was observed between brain CBD concentrations and cSI scores (*r* = −0.70, *p* = 0.005), supporting a contribution of central drug exposure to the anticonvulsant response (Figure 4D).

Collectively, these findings establish CBD as an effective anticonvulsant in the GASH/Sal model, reducing audiogenic seizure severity in a time-dependent manner: the effect appeared acutely, within 1 h of administration, and intensified progressively under chronic dosing to reach its maximum at 14 days. The response was numerically greater and more sustained in females than in males, but this apparent sex difference did not reach statistical significance in any direct comparison and should be regarded as an exploratory observation warranting confirmation in an adequately powered study. Finally, seizure severity was inversely correlated with both serum and brain CBD concentrations, pointing to a pharmacokinetically driven, exposure-dependent component of the anticonvulsant effect.

### 3.3 CBD treatment modifies audiogenic seizure behaviors over time

**FIGURE 5:**
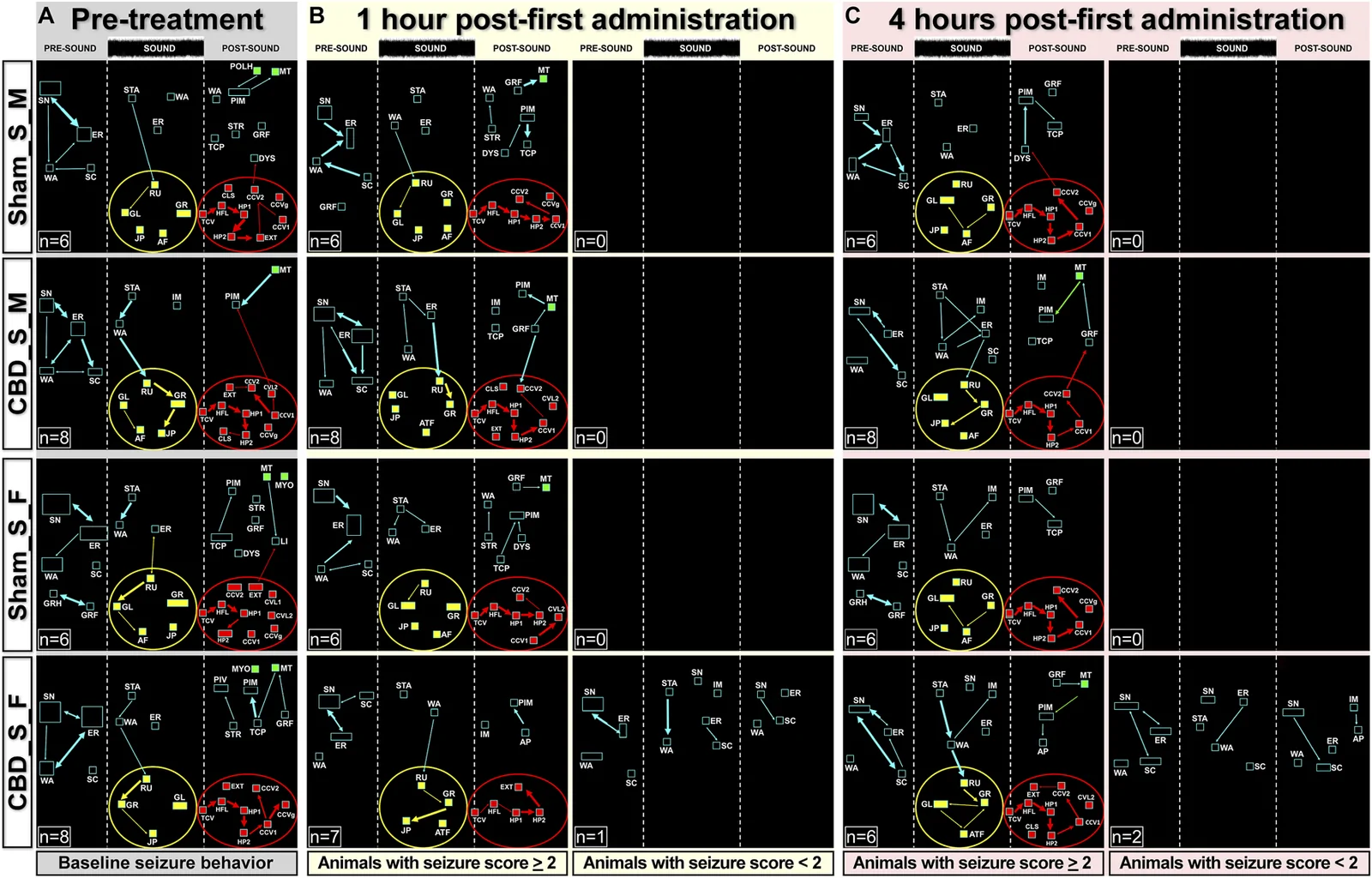
Effects of acute CBD treatment on audiogenic seizure behavior. **(A)** Baseline behavioral flowcharts from male and female GASH/Sal animals (assigned to CBD or vehicle groups) prior to treatment initiation. All animals exhibited complete audiogenic seizures, beginning with the wild running phase—characterized by turns to the left (GL) or right (GR), running (RU), jumping (JP), and atonic falls (AF) (yellow circle)—followed by tonic–clonic convulsions (TCV, CCV1, CCV2, CCVg) and generalized clonic seizures with hyperextensions (HP1, HP2) (red circle). Seizure episodes ended with postictal immobility (PIM) and respiratory distress (dyspnea, DYS; tachypnea, TCP). **(B)** Behavioral flowcharts recorded 1 h after the first intraperitoneal administration of CBD (200 mg/kg) or vehicle. In CBD-treated animals, seizure behaviors from both the wild running and convulsive phases were markedly reduced. In CBD-treated females (CBD_S_F), convulsive behaviors such as CCV1, CCV2, and CCVg were absent in animals with seizure scores ≥ 2, and one female displayed a seizure score < 2, indicating complete suppression of seizure activity. In CBD-treated males (CBD_S_M), CCVg was absent, while CCV1 and CCV2 were still observed. **(C)** Flowcharts of the same groups assessed 4 h after the first administration. Notably, two CBD-treated females showed seizure severity scores < 2, with complete absence of seizure-related behaviors. Flowcharts represent the collective behavioral profiles of all animals within each experimental group. Seizure activity was analyzed across three temporal windows: pre-sound, during sound, and post-sound. The number of animals (n) that exhibited the corresponding seizure-related behaviors is displayed in each panel for each experimental group. For interpretation of flowchart structure and behavior coding, refer to Figure 2.

**FIGURE 6:**
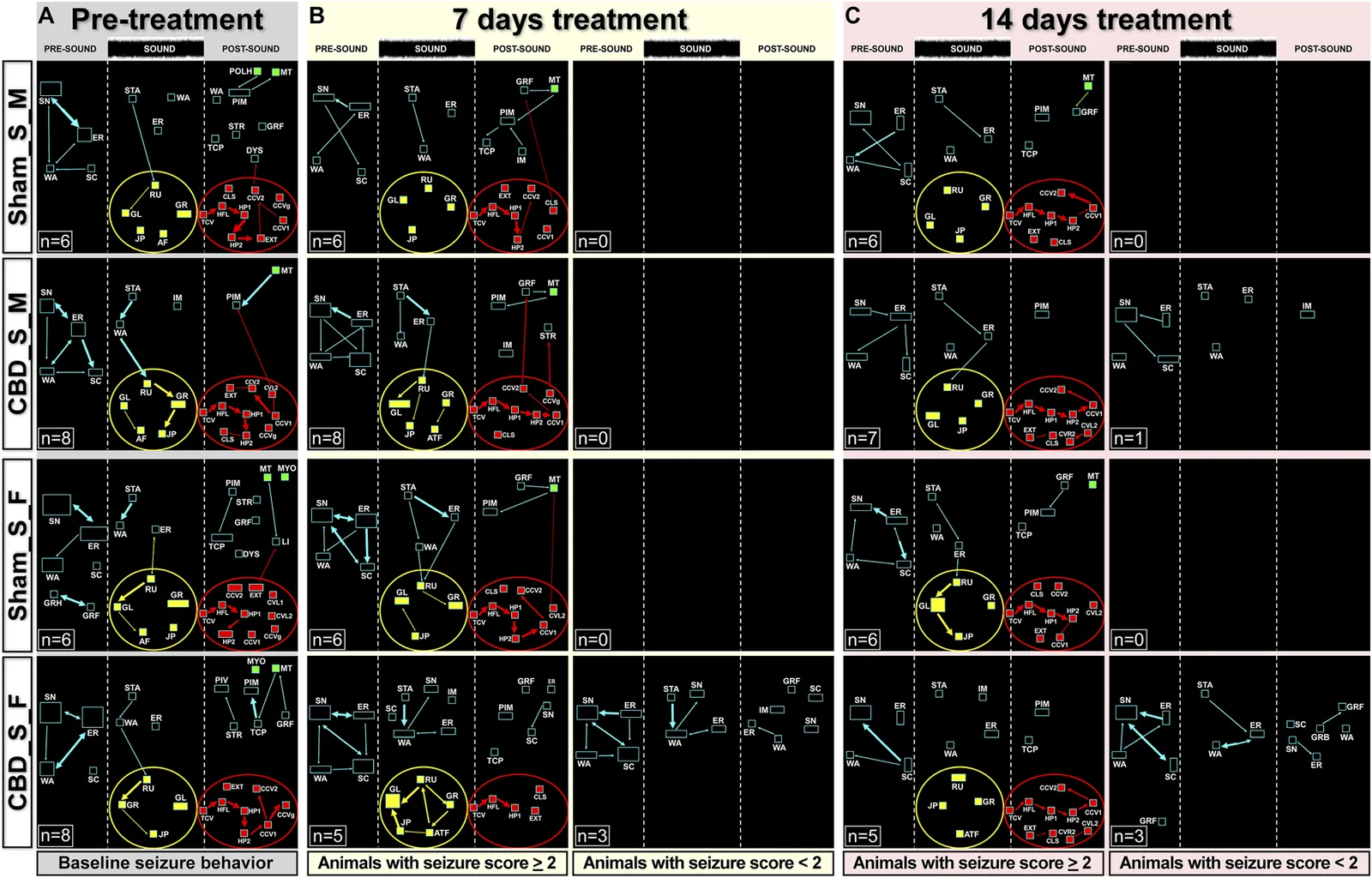
Effects of chronic CBD treatment on audiogenic seizure behavior. **(A)** Baseline behavioral flowcharts from male and female GASH/Sal animals (assigned to CBD or vehicle groups) prior to treatment. All animals exhibited complete audiogenic seizures, starting with the wild running phase—characterized by left (GL) or right (GR) turning, running (RU), jumping (JP), and atonic falls (AF) (yellow circle)—followed by tonic–clonic convulsions (TCV, CCV1, CCV2, CCVg) and generalized clonic seizures with hyperextensions (HP1, HP2) (red circle). Seizure episodes ended with postictal immobility (PIM) and respiratory distress (dyspnea, DYS; tachypnea, TCP). **(B)** Behavioral flowcharts after 7 days of chronic CBD administration (200 mg/kg, every 12 h). In CBD-treated females, seizure behaviors from both the wild running and convulsive phases were notably reduced (e.g., AF and CCVg), while male responses remained largely unchanged. Three CBD-treated females exhibited seizure severity scores < 2, with complete suppression of seizure activity. **(C)** Flowcharts after 14 days of chronic treatment. At this endpoint, three CBD-treated females and one male displayed seizure scores < 2, showing complete absence of seizure-related behaviors. Flowcharts represent the combined behavioral repertoire of all animals within each experimental group. Seizure activity was analyzed across three temporal windows: pre-sound, during sound, and post-sound. The number of animals (n) that exhibited the corresponding seizure-related behaviors is displayed in each panel for each experimental group. For flowchart structure and behavioral code definitions, see Figure 2.

To gain deeper insight into the effects of CBD, we systematically examined seizure-related behaviors across all experimental groups. A neuroethological approach was applied to characterize seizure expression using a validated behavioral dictionary (Figure 2A), alongside the categorized seizure severity index (cSI) data (Figures 4A,B). Prior to treatment, all GASH/Sal animals displayed the complete repertoire of AGS behaviors in response to high-intensity acoustic stimulation, in line with previous findings (Muñoz et al., 2017). Representative examples of these behaviors in untreated animals are presented in Figure 2C. Seizures began within 1–3 s of stimulus onset and followed a stereotyped sequence of five behavioral phases: behavioral arrest, wild running (∼5 s), tonic–clonic convulsions (∼33 s), head ventral flexion with forelimb and hindlimb extension, and finally postictal immobility (stupor). After this sequence, animals recovered and returned to exploratory behavior similar to their pre-stimulus baseline. As expected, all male and female GASH/Sal subjects exhibited the full seizure phenotype before the onset of treatment (Figures 5A, 6A). During the pre-sound window, animals predominantly engaged in exploratory behaviors such as sniffing (SN) and walking (WA). In the sound window, approximately 7 s after stimulus onset, wild running behaviors were consistently observed—including lateral turning (GL, GR), running (RU), jumping (JP), and atonic falls (AF). Post-sound, animals transitioned into the convulsive phase, characterized by tonic convulsions (TCV), followed by generalized clonic convulsions (CCV1, CCV2, CCVg), hyperextensions (HP1, HP2), and postictal immobility (PIM), often accompanied by respiratory distress (dyspnea, DYS; tachypnea, TCP) (Figures 5A, 6A).

Animals were then assigned to receive either vehicle (sham) or CBD according to the acute and subsequent chronic treatment regimens. In vehicle-treated animals, seizure behavior remained unchanged throughout the study. At both 1- and 4-h post-vehicle administration, all males and females displayed behavioral profiles similar to their pre-treatment baseline, including the full wild running and convulsive sequences (Figures 5B,C). After 7 and 14 days of repeated vehicle injections, seizure behaviors were similarly maintained (Figures 6B,C), indicating that neither handling procedures nor the vehicle itself had any measurable effect on seizure activity. In contrast, CBD-treated animals exhibited attenuation of seizure-related behaviors over time, with a numerically greater effect in females. In males, acute CBD administration produced mild anticonvulsant effects: both at 1- and 4-h post-injection, generalized clonic convulsions (CCVg) were abolished, but convulsive behaviors involving the limbs (CCV1, CCV2) remained partially visible, and all animals retained seizure severity scores ≥ 2 (Figures 5B,C). In females, however, acute CBD treatment produced a more pronounced reduction in seizure-related behaviors compared to males. One hour after administration, all convulsive behaviors (CCVg, CCV1, CCV2) were absent, and one female presented complete seizure suppression (cSI = 0). By 4 h post-administration, two CBD-treated females showed total abolition of seizure behaviors (Figures 5B,C).

With chronic CBD administration, behavioral improvements became more pronounced. In males, seizure-related behaviors remained mildly reduced after 7 days, similar to the acute condition (Figure 6B). After 14 days, further attenuation was observed, including one male with complete suppression of seizure behaviors (Figure 6C). In females, the chronic CBD treatment produced a robust anticonvulsant effect. By day 7, five CBD-treated females showed no convulsion-associated behaviors (CCVg, CCV1, CCV2), and three animals exhibited complete suppression of seizure activity (Figure 6B). At day 14, five animals with residual seizures (cSI ≥ 2) showed fewer behaviors in the wild running phase but re-expressed more convulsive elements compared to day 7. All remaining CBD-treated females displayed complete suppression of seizure behaviors (Figure 6C).

### 3.4 Effects of CBD treatment on seizure latency and duration of seizure phases

Seizure latency and the durations of the wild running and convulsive phases were analyzed in acoustically stimulated animals using linear mixed-effects models fitted by restricted maximum likelihood (REML), comparing Sham versus CBD separately within each sex as well as males vs. females within each treatment group. Animals with complete seizure suppression (cSI = 0) were excluded, as these parameters cannot be measured in the absence of a seizure. Denominator degrees of freedom for the within-subject (time) factor were corrected for non-sphericity using the Geisser–Greenhouse ε (reported in Supplementary Material 3), and pairwise comparisons were adjusted with the Šídák method.

**FIGURE 7:**
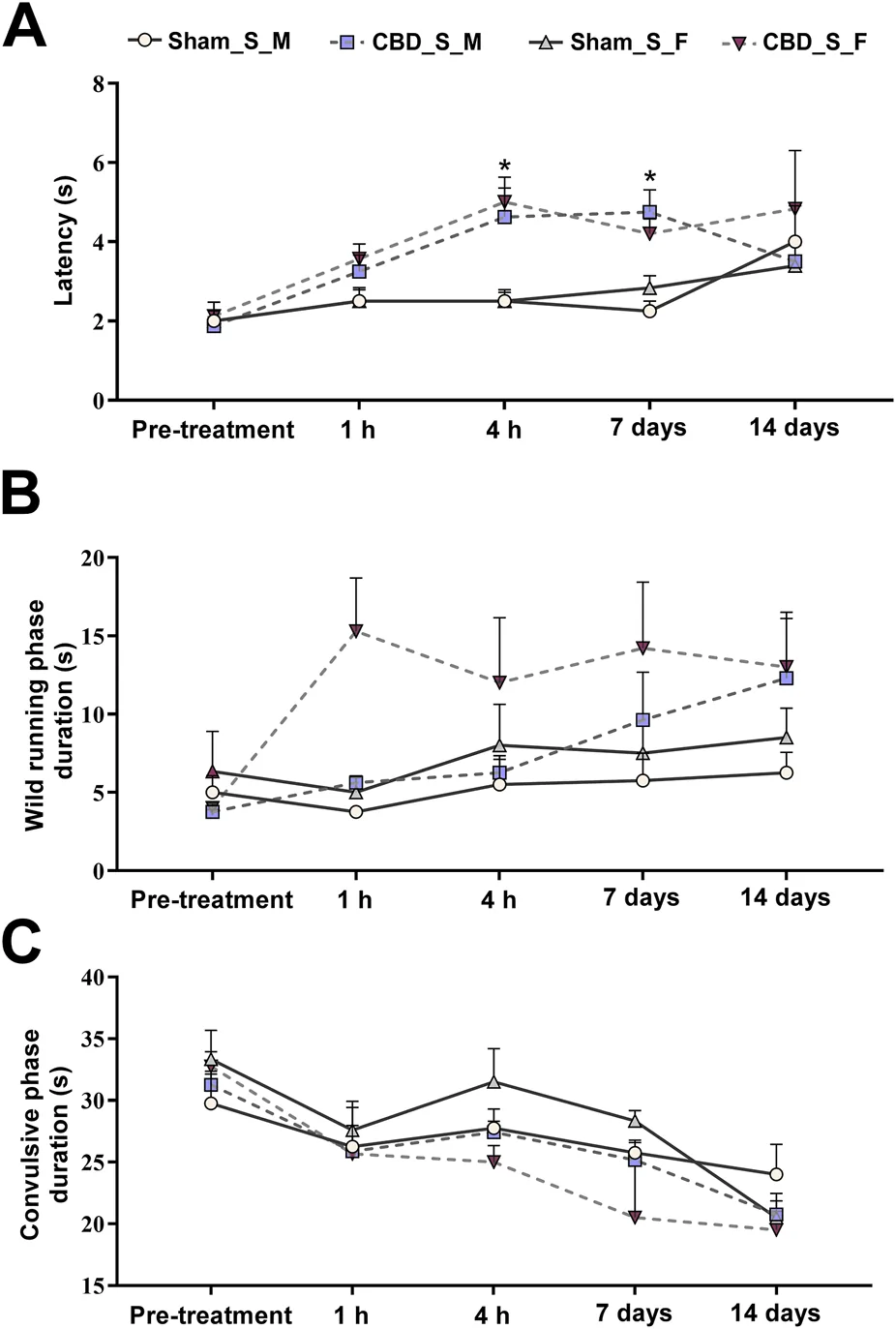
Effects of acute and chronic CBD treatment on seizure latency and phase durations. **(A)** Latency to seizure onset after acoustic stimulation in male and female GASH/Sal animals treated with CBD (200 mg/kg) or vehicle (sham) across baseline, acute (1 and 4 h), and chronic (days 7 and 14) time points. CBD increased seizure latency relative to sham in both sexes, with the within-sex Sham–CBD difference reaching significance at 4 h in females and at day 7 in males (direct male–female comparisons were not significant), consistent with an earlier effect in females and a delayed, chronic-phase effect in males. **(B)** Duration of the wild running phase tended to be longer in CBD-treated females than in sham-treated females, most notably at 1 h after the first injection; this difference did not reach significance at any individual time point, although the analysis revealed a significant effect of time and a significant Time × Treatment interaction in females. **(C)** Mean convulsive-phase duration in CBD-treated animals of both sexes tended to decrease across the treatment period, reaching its lowest values at day 14; in females this corresponded to a significant overall effect of treatment (lower convulsive duration than sham), whereas no significant treatment effect was detected in males. Animals with complete seizure suppression (cSI = 0) were excluded from all panels, as latency and seizure phase durations cannot be measured in the absence of seizures. Data were analyzed using mixed-effects models (restricted maximum likelihood, REML) followed by Šídák’s multiple comparisons test for the pre-specified comparisons (full statistical output in Supplementary Material 3). Statistically significant differences between treatment groups (Sham vs. CBD within each sex) are indicated as * (*p < 0.05*). All variables (seizure latency and phase durations) are measured in seconds (s). Data are presented as mean ± SEM.

For seizure latency (Figure 7A), the model revealed a significant Time × Treatment interaction in males (F_(4, 38)_ = 3.10, *p = 0.027*) and a significant main effect of time (F_(2.88, 27.32)_ = 3.98, *p* = 0.019), with the treatment effect approaching significance (F_(1, 10)_ = 4.81, *p = 0.053*). Šídák-adjusted comparisons localized this effect to day 7, where CBD-treated males showed a 2.5-s longer latency than sham males (*p = 0.013*). In females, latency was governed by a significant main effect of treatment (F_(1, 51)_ = 12.03, *p = 0.001*), without a significant interaction (F_(4, 51)_ = 1.10, *p = 0.367*). Here, the increase reached significance earlier, at 4 h post-injection (Δ = 2.5 s; *p = 0.045*). CBD therefore prolonged latency to seizure onset in both sexes, but earlier in females (4 h) than in males (day 7).

For the wild running phase (Figure 7B), females showed a significant main effect of time (F_(2.87, 27.98)_ = 3.10, *p = 0.044*) and a significant Time × Treatment interaction (F_(4, 39)_ = 2.93, *p = 0.033*), indicating a treatment-dependent change in the temporal profile of this phase. No individual timepoint, however, reached significance after Šídák adjustment (all adjusted *p ≥ 0.109*), including the transient increase in CBD-treated females at 1 h (Δ = 10.3 s; *p = 0.109*). In males, no effect of time, treatment, or their interaction was detected (all *p ≥ 0.184*).

For the convulsive phase (Figure 7C), CBD-treated females exhibited a significant main effect of treatment (F_(1, 12)_ = 5.33, *p = 0.040*), reflecting a shorter overall convulsive duration than sham females, together with a significant effect of time (F_(2.41, 16.84)_ = 9.22, *p = 0.001*); the interaction was not significant (F_(4, 28)_ = 1.17, *p = 0.345*). No single timepoint survived Šídák adjustment (all adjusted *p ≥ 0.298*). In males, convulsive duration varied with time (F_(2.44, 20.12)_ = 5.16, *p = 0.012*) but was unaffected by treatment (F_(1, 10)_ = 0.16, *p = 0.699*).

Across all three parameters, no significant differences were detected between males and females within either the sham or the CBD group (all Šídák-adjusted *p > 0.05*). Collectively, CBD prolonged seizure latency in both sexes (reaching significance at 4 h within females and at day 7 within males) and, within the female group, significantly altered the temporal structure of the wild running phase and reduced overall convulsive duration; direct male–female comparisons were not significant for any parameter.

### 3.5 Effects of CBD treatment on body weight, hematological and serum biochemical profiles

**FIGURE 8:**
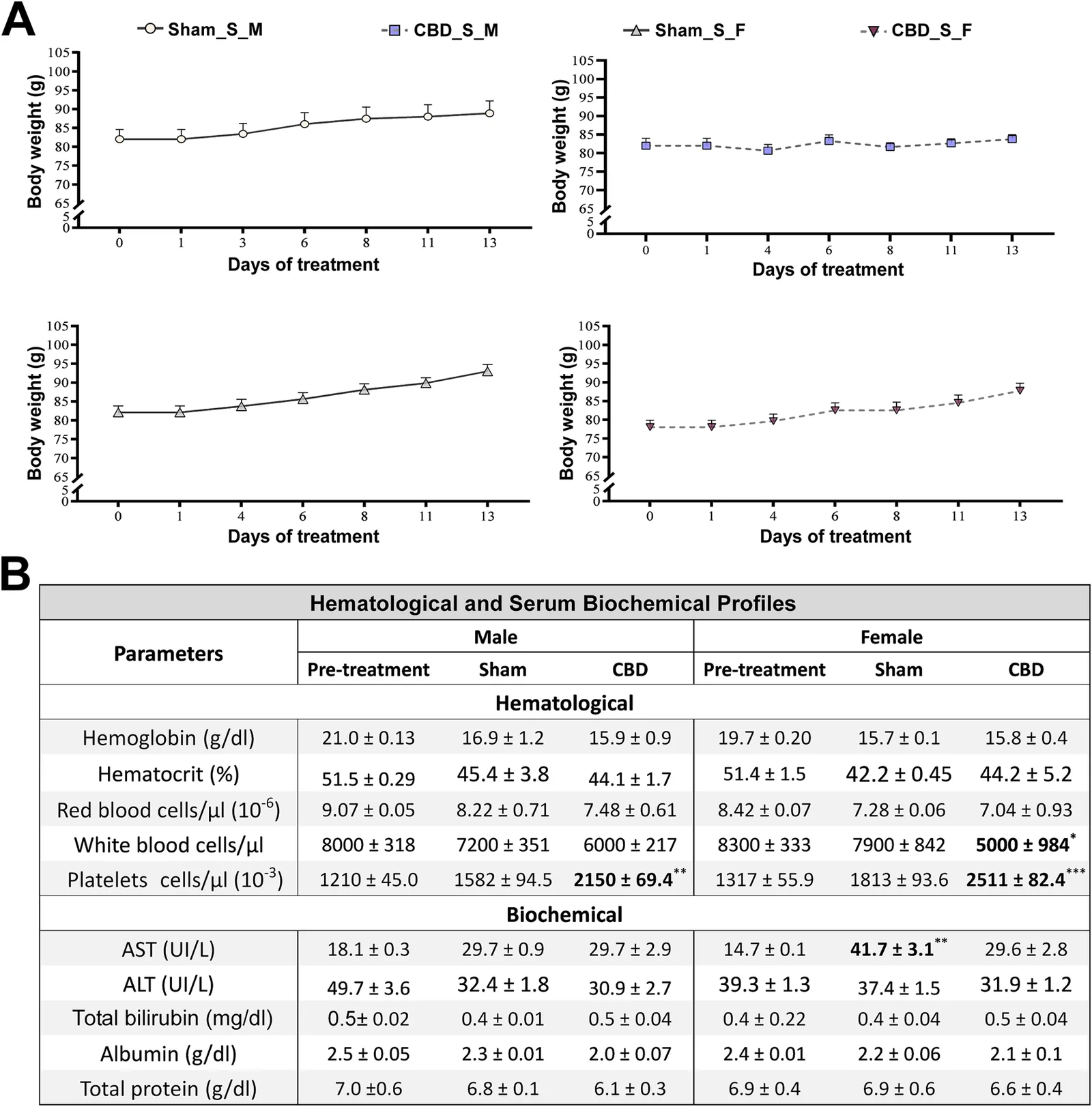
Effects of CBD treatment on body weight, hematological and biochemical profiles. **(A)** Graphs display the time course of body weight changes over the 13-day treatment period in male and female GASH/Sal animals treated with vehicle (Sham) or CBD. Baseline body weights (time 0 on the x-axis) were statistically comparable between treatment groups within each sex (females: Sham 82.1 ± 1.8 g vs. CBD 78.0 ± 1.9 g, *p = 0.13*; males: Sham 82.0 ± 2.5 g vs. CBD 82.0 ± 1.9 g, *p = 1.00*; unpaired t-test). Body weight was analysed using a mixed-effects model (REML), with treatment and sex as between-subject factors and time as a within-subject repeated measure. All groups followed parallel growth trajectories, indicating no CBD-related effects on body weight gain. **(B)** The table summarizes the hematological and biochemical profiles of GASH/Sal animals following 14 days of chronic treatment. Hematological parameters include hemoglobin concentration, hematocrit (percentage of red blood cells in blood), red blood cell count, white blood cell count, and platelet count (per µl). Liver function was assessed through five biochemical markers: aspartate aminotransferase (AST), alanine aminotransferase (ALT), total bilirubin, albumin, and total protein levels. Parameters were analyzed by two-way ANOVA (treatment and sex) followed by Tukey’s *post hoc* test. Asterisks and bold values in the table indicate statistically significant differences (*p < 0.05, **p < 0.01, ***p < 0.001) in CBD-treated animals compared to sham controls. Data are presented as means ± S.E.M.

To assess the safety and tolerability of chronic CBD administration, we monitored body weight and evaluated hematological and serum biochemical parameters across all experimental groups (Figure 8). Baseline body weights were statistically comparable between treatment groups within each sex (females: Sham vs. CBD, *p = 0.13*; males: Sham vs. CBD, *p = 1.00*; unpaired t-tests), confirming successful randomization. Body weight was monitored three times per week throughout the treatment period, and no adverse effects associated with chronic CBD administration were observed (Figure 8A). Analysis using a mixed-effects model, with time included as a repeated measure and treatment and sex as fixed factors, revealed a significant effect of time (F_(6,189)_ = 7.416, *p < 0.0001*) and sex (F_(1,189)_ = 24.24, *p < 0.0001*), consistent with normal growth progression and expected sex-related differences in body weight. In contrast, no significant effect of treatment was detected (F_(1,189)_ = 0.1291, *p = 0.7198*), and no significant interactions were observed for time × treatment, treatment × sex, or time × treatment × sex. Although minor numerical differences in weekly weight gain were observed between groups, all animals displayed comparable weight trajectories over time (Figure 8A). In females, sham-treated animals gained an average of 3.5 g during the first week and 7.4 g during the second week, whereas CBD-treated females gained 4.5 g and 5.3 g, respectively. In males, sham animals gained an average of 4.0 g during the first week and 2.9 g during the second week, while CBD-treated males gained 1.3 g and 0.5 g over the same periods. Overall, these findings indicate that chronic CBD administration did not alter normal growth patterns, feeding behavior or general health status in either sex during the experimental period.

Hematological parameters remained largely within normal physiological ranges, with no significant differences between groups under pre-treatment conditions (Figure 8B). After 14 days of treatment, hemoglobin levels, hematocrit, and red blood cell counts showed no significant changes in CBD-treated animals compared to their corresponding sham controls, in either sex. However, a significant reduction in white blood cell count was observed in CBD-treated females relative to sham controls (5,000 ± 984 cells/μL vs. 7,900 ± 842 cells/μL, *p < 0.01*). No such difference was found in males. Conversely, platelet counts significantly increased in both CBD-treated males (2,150 ± 69.4 ×10^3^ cells/μL) and females (2,511 ± 82.4 ×10^3^ cells/μL) compared to their respective sham groups (1,582 ± 94.5 ×10^3^ cells/μL in males, *p < 0.01*; 1813 ± 93.6 ×10^3^ cells/μL in females, *p < 0.001*), suggesting a potential modulatory effect of CBD on thrombopoiesis.

Liver function was assessed using five serum biochemical markers: aspartate aminotransferase (AST), alanine aminotransferase (ALT), total bilirubin, albumin, and total protein (Figure 8B). After 14 days of treatment, AST levels were elevated in both sham-treated males and females compared to their pre-treatment values, reaching statistical significance in females (*p < 0.01*), and hence, suggesting a procedural or time-dependent physiological response. However, CBD-treated females exhibited significantly lower AST levels than their sham counterparts (29.6 ± 2.8 U/L vs. 41.7 ± 3.1 U/L, *p < 0.01*), indicating absence of hepatotoxicity. No significant differences in AST or ALT levels were found in males between CBD and sham groups. Furthermore, no statistically significant alterations were observed in serum bilirubin, albumin, or total protein levels across any group, reinforcing the overall hepatic safety of the chronic CBD regimen. Together, these findings indicate that 14 days of twice-daily intraperitoneal CBD administration (200 mg/kg) are well tolerated in GASH/Sal animals, with no adverse effects on body weight, hematological profiles, or liver function, suggesting no systemic toxicity.

### 3.6 Global transcriptional profiles in the inferior colliculus across chronic treatment, acoustic seizure stimulation and sex

**FIGURE 9:**
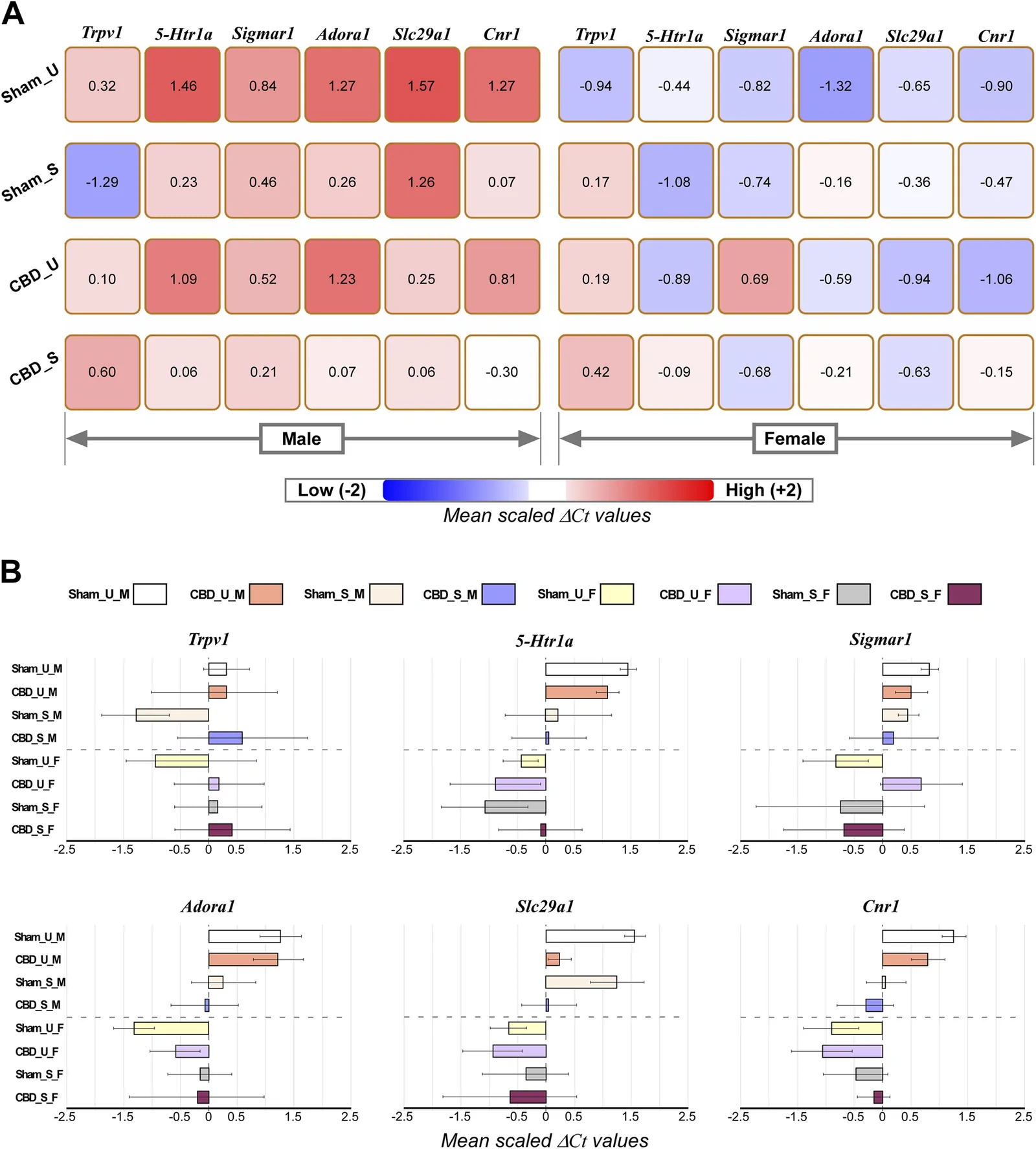
Global gene expression profiles in the inferior colliculus across chronic treatment, acoustic seizure stimulation and sex. **(A)** Heatmap displaying the mean scaled ΔCt values (z-score) of *Trpv1*, *5-Htr1a*, *Sigmar1*, *Adora1*, *Slc29a1*, and *Cnr1* in the inferior colliculus of male and female GASH/Sal animals treated with CBD or vehicle (Sham), with or without acoustic seizure induction. The color gradient represents the mean scaled relative expression, where red indicates higher expression (up to +2) and blue indicates lower expression (down to −2) relative to the gene mean. Columns represent individual gene targets, while rows represent experimental groups: Sham_U_M (male sham, unstimulated), CBD_U_M (male CBD, unstimulated), Sham_S_M (male sham, stimulated), CBD_S_M (male CBD, stimulated), Sham_U_F (female sham, unstimulated), CBD_U_F (female CBD, unstimulated), Sham_S_F (female sham, stimulated), and CBD_S_F (female CBD, stimulated). **(B)** Horizontal bar plots illustrating the distribution of mean scaled ΔCt values (z-score) ± standard deviation (SD) for each gene target across the eight experimental groups. A dashed vertical line at zero denotes the mean gene expression level across all samples. A dashed horizontal line separates the male (upper) from the female (lower) subgroups within each panel. All six panels share a fixed x-axis range of −2.5 to +2.5 standard deviation units to enable direct inter-gene comparison of effect magnitudes. Note that sex emerges as the dominant source of variance in the inferior colliculus gene expression profiles across this six-gene panel, with stimulation seizure condition exerting a secondary and gene-specific modulating influence, most conspicuous in males. n = 4–7 biological samples per experimental group.

To investigate molecular mechanisms associated with chronic CBD treatment and seizure activity, we quantified the expression of six genes related to excitability and neuroprotection—*Trpv1, 5-Htr1a, Sigmar1, Adora1, Slc29a1,* and *Cnr1*—in the inferior colliculus. Scaled ΔCt values (z-scores) were calculated to provide an overview of transcriptional patterns across the eight experimental groups and were visualized using heatmaps and bar plots (Figure 9; Supplementary Material 4). This global analysis revealed a clear primary segregation of samples by sex. Male animals were characterized by broadly positive z-scores across *5-Htr1a*, *Sigmar1*, *Adora1*, *Slc29a1*, and *Cnr1*, indicating above-average expression of these transcripts relative to the dataset mean. By contrast, female animals displayed predominantly negative z-scores for the same genes, reflecting below-average expression. The magnitude of this sex-associated divergence was gene-dependent: *Slc29a1* and *5-Htr1a* showed the largest male–female difference in mean z-score across all conditions, followed by *Adora1*, *Cnr1*, and *Sigmar1* (Figure 9A). *Trpv1* was a notable exception, exhibiting a weaker and more heterogeneous sex-related pattern with substantial within-group variability in both sexes (Figure 9A).

Within the male group, a secondary stratification according to seizure stimulation condition was evident for several genes. Unstimulated males (Sham_U_M and CBD_U_M) showed the highest z-scores for *Adora1* and *Slc29a1*, whereas AGS stimulation reduced these values, particularly in CBD-treated stimulated males (CBD_S_M; Figure 9B; Supplementary Material 4). For *5-Htr1a*, unstimulated Sham males exhibited the highest group mean in the entire dataset, with modest attenuation following CBD treatment under the same condition and a more pronounced reduction under seizure stimulated conditions regardless of treatment (Sham_S_M and CBD_S_M; Figure 9B; Supplementary Material 4). *Cnr1* exhibited a comparable pattern in males, with the highest mean z-score observed in Sham_U_M animals. This elevation was attenuated by CBD treatment under unstimulated conditions and declined further following seizures, such that CBD-treated stimulated males (CBD_S_M) approached the range observed in females (Figure 9B; Supplementary Material 4).

In females, expression of *Adora1*, *Slc29a1*, and *Cnr1* was uniformly below the gene grand mean across nearly all groups and conditions. The most pronounced negative z-scores in the entire dataset were observed in Sham_U_F for *Adora1*. CBD treatment partially attenuated this suppression in unstimulated females (CBD_U_F: Figure 9B) and, notably, was associated with a positive shift in *Sigmar1* expression in unstimulated females (Sham_U_F vs. CBD_U_F: Figure 9B), representing one of the few descriptive increases observed in females (Figure 9B; Supplementary Material 4).

*Trpv1* displayed the most heterogeneous pattern across groups. Under basal conditions, female Sham animals exhibited below-average expression (Sham_U_F: Figure 9B), whereas the combination of CBD treatment and stimulation yielded the most positive *Trpv1* z-scores among all female groups (CBD_S_F: Figure 9B). In males, vehicle-treated stimulation was associated with the lowest *Trpv1* mean expression (Sham_S_M; Figure 9B), whereas CBD treatment under stimulated conditions reversed this pattern (CBD_S_M; Figure 9B), suggesting that CBD led a context-dependent normalization of stimulation-associated *Trpv1* suppression.

Collectively, these descriptive analyses identify sex as the dominant source of variance in inferior colliculus gene expression across the six-gene panel, with seizure stimulation exerting a secondary, gene-specific influence that was most evident in males. CBD treatment produced heterogeneous effects that varied according to sex, stimulation condition, and target gene (Figure 9; Supplementary Material 4).

### 3.7 Sex as the primary determinant of inferior colliculus gene expression with secondary modulation by CBD and seizure activity

**FIGURE 10:**
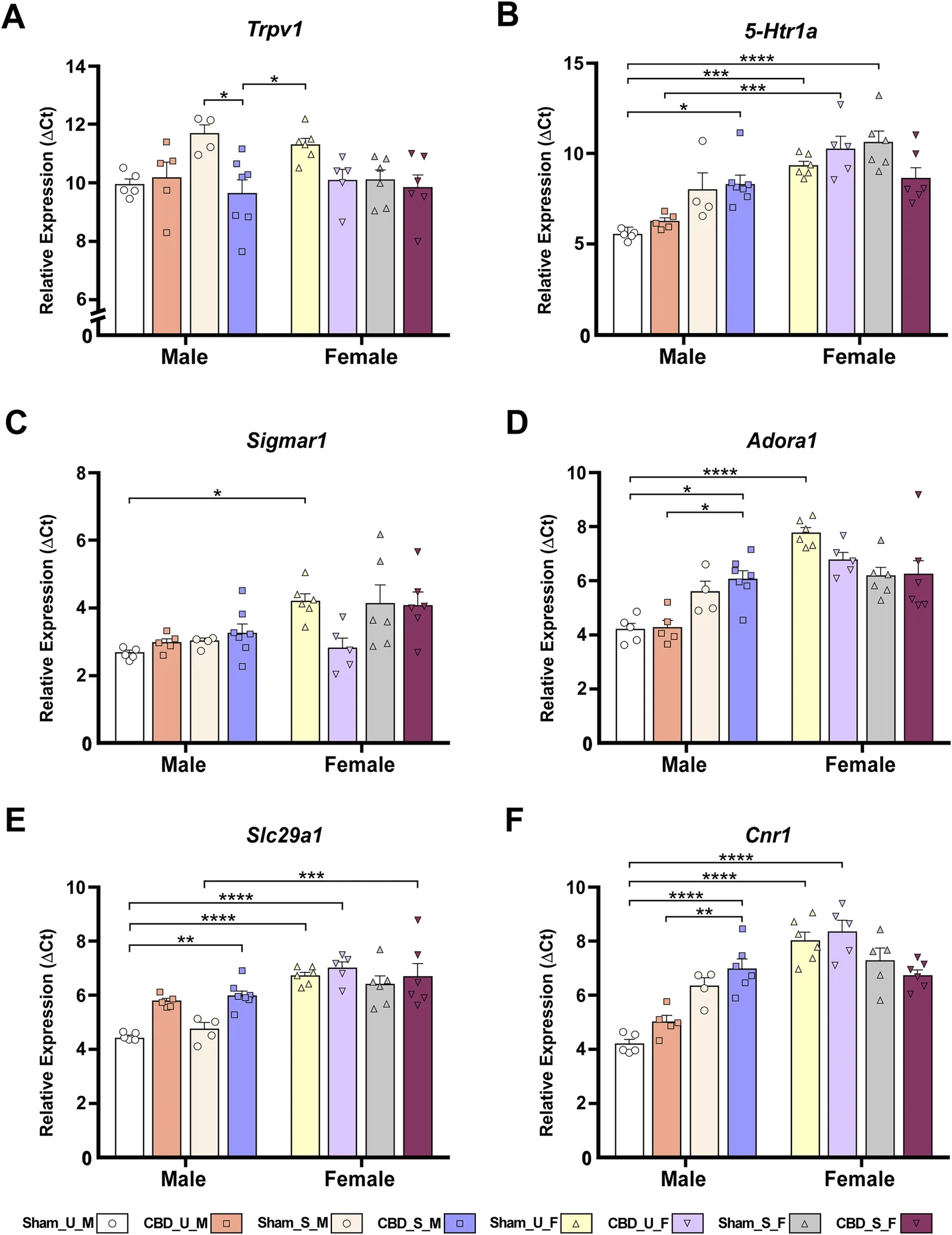
Sex-, treatment-, and seizure-dependent modulation of gene expression in the inferior colliculus. Relative mRNA expression levels of *Trpv1* **(A)**, *5-Htr1a* **(B)**, *Sigmar1* **(C)**, *Adora1* **(D)**, *Slc29a1* **(E)**, and *Cnr1* **(F)** were quantified by RT-qPCR in the inferior colliculus of male and female GASH/Sal animals treated with cannabidiol (CBD) or vehicle (Sham), with or without acoustic seizure induction. Expression values are shown as mean ΔCt values normalized to β-actin (mean ± SEM). Individual biological replicates are overlaid on each bar. Experimental groups were: Sham_U_M (male sham, unstimulated), CBD_U_M (male CBD, unstimulated), Sham_S_M (male sham, stimulated), CBD_S_M (male CBD, stimulated), Sham_U_F (female sham, unstimulated), CBD_U_F (female CBD, unstimulated), Sham_S_F (female sham, stimulated), and CBD_S_F (female CBD, stimulated). Data were analyzed by three-way ANOVA (sex × treatment × condition) followed by Tukey’s multiple comparisons test. Horizontal brackets indicate the most biologically relevant significant *post hoc* pairwise comparisons; complete three-way ANOVA outputs and Tukey’s multiple-comparisons results for all 28 pairwise combinations per gene are provided in Supplementary Material 5. Asterisks denote statistical significance (*p < 0.05, **p < 0.01, ***p < 0.001, ****p < 0.0001). n = 4–7 biological replicates per group.

To formally determine the contribution of sex, chronic CBD treatment, and AGS stimulation to transcriptional regulation in the inferior colliculus of the GASH/Sal model, ΔCt values for *Trpv1, 5-Htr1a, Sigmar1, Adora1, Slc29a1,* and *Cnr1* were analyzed by three-way ANOVA (Sex × Treatment × Stimulation Condition) (Figure 10; Table 1; Supplementary Material 5).

**TABLE 1:** Summary of three-way ANOVA effects on inferior colliculus gene expression.

Gene	Sex	Treatment	Condition (unstimulated vs. stimulated)	Sex × treatment	Sex × Condition	Treatment × Condition	Sex × Treatment × Condition
*Trpv1*	ns	ns	**	*	ns	ns	**
*5-Htr1a*	****	**	ns	**	ns	*	ns
*Sigmar1*	**	ns	ns	ns	*	ns	ns
*Adora1*	****	ns	ns	****	ns	ns	ns
*Slc29a1*	****	ns	***	ns	*	ns	ns
*Cnr1*	****	ns	ns	****	ns	ns	ns

Effects of Sex, Treatment (CBD), and Condition (Unstimulated vs. Stimulated) on relative mRNA expression (ΔCt values) for *Trpv1*, *5-Htr1a*, *Sigmar1*, *Adora1*, *Slc29a1*, and *Cnr1*. Statistical significance is denoted as follows: *p < 0.05, **p < 0.01, ***p < 0.001, and ****p < 0.0001; *ns* indicates a non-significant result (*p > 0.05*).

Across the gene panel, biological sex emerged as the most robust and consistent determinant of transcriptional variation. Significant main effects of sex were detected for *5-Htr1a* (F_(1, 36)_ = 48.84, *p < 0.0001*), *Adora1* (F_(1, 36)_ = 40.86, *p < 0.0001*), *Slc29a1* (F_(1, 36)_ = 57.23, *p < 0.0001*), and *Sigmar1* (F_(1, 36)_ = 12.57, *p = 0.001*) and *Cnr1* (F_(1, 34)_ = 71.26, *p < 0.0001*), while *Trpv1* was notably the only gene unaffected by sex (*p = 0.9236*) (Table 1). Consistent with the global z-score profiles, females generally exhibited higher ΔCt values, indicating lower relative mRNA abundance than males. Sex explained a particularly large proportion of the variance for *5-Htr1a* (44.31%), *Slc29a1* (51.05%) and *Cnr1* (43.69%), highlighting marked sexual dimorphism in these pathways (Supplementary Material 5).

The effect of seizure stimulation was selective rather than global. Significant main effects of condition (stimulated vs. unstimulated) were observed only for *Trpv1* (F_(1,36)_ = 8.45, *p = 0.0062*) and *Slc29a1* (F_(1,36)_ = 16.60, *p = 0.0002*), indicating that seizure activity preferentially triggers rapid transcriptional remodeling in genes involved in neuronal excitability and nucleoside-mediated neuromodulation (Table 1; Supplementary Material 5).

The stimulation condition-dependent responses were further modified by sex for specific genes. Significant Sex × Condition interactions were detected for *Sigmar1* (F_(1,36)_ = 4.55*, p = 0.0397*) and *Slc29a1* (F_(1,36)_ = 6.74, *p = 0.0135*), indicating sex-specific transcriptional responses to acoustic seizure stimulation. No independent stimulation condition effects were detected for *5-Htr1a, Sigmar1, Adora1,* or *Cnr1* (all *p* > 0.05) (Table 1; Supplementary Material 5).

Chronic CBD treatment did not exert a uniform transcriptional effect across the gene panel. Instead, it targeted specific neurochemical pathways. A significant main effect of treatment was identified only for *5-Htr1a* (F_(1,36)_ = 7.58, *p = 0.0092*), whereas *Cnr1* showed a trend toward significance (*p = 0.0668*). No main treatment effects were detected for *Trpv1, Sigmar1, Adora1,* or *Slc29a1* (Table 1; Supplementary Material 5). These findings indicate that the principal transcriptional signature of chronic CBD treatment in the inferior colliculus involves serotonergic signaling, with a possible contribution of cannabinoid pathways.

Several higher-order interactions further demonstrated that CBD responses were strongly context dependent. Significant Sex × Treatment interactions were observed for *Cnr1* (F_(1,34)_ = 47.83, *p < 0.0001*) and *Adora1* (F_(1,36)_ = 24.62, *p < 0.0001*), indicating markedly different treatment responses between males and females. *5-Htr1a* also showed significant Sex × Treatment (F_(1,36)_ = 10.10, *p = 0.0030*) and Treatment × Condition (F_(1,36)_ = 4.63, *p = 0.0382*) interactions, demonstrating modulation of CBD effects by both sex and seizure state. For *Trpv1*, a significant three-way Sex × Treatment × Condition interaction was detected (F_(1,36)_ = 8.10, *p = 0.0073*), indicating that regulation of this transcript depended on the combined influence of sex, CBD exposure, and stimulation condition (Table 1).

Tukey’s *post hoc* comparisons supported these interaction effects, with significant pairwise differences occurring more frequently in males than in females (Figure 10). In males, CBD treatment and/or stimulation commonly shifted expression away from unstimulated Sham levels, whereas female groups were comparatively stable across conditions (Figure 10; Supplementary Material 5).

Overall, these analyses identify sex as the principal determinant of the transcriptional environment in the GASH/Sal inferior colliculus, while chronic CBD treatment and seizure stimulation act as secondary, gene-specific modulators whose effects depend strongly on biological context.

## 4 Discussion

This study suggests that CBD exerts time-dependent anticonvulsant effects in the GASH/Sal model of audiogenic seizures accompanied by a sex-dependent molecular organization of the epileptogenic focus. Pharmacokinetic analyses indicated that both acute and chronic intraperitoneal administration of CBD (200 mg/kg) produced measurable and sustained exposure in serum and brain tissue, with notable inter-individual variability in the measured concentrations. Peak systemic concentrations were observed 1-h post-injection and steady-state concentrations were achieved by day 5 of chronic treatment. Behavioral and neuroethological assessments revealed a significant reduction in seizure severity that was numerically greater in females than in males, although this sex difference was not statistically significant; a substantial proportion of females achieved complete suppression (cSI = 0) after chronic dosing. In males, the anticonvulsant effects were more modest and emerged progressively over the course of treatment. Correlation analyses further indicated that higher CBD concentrations in serum and brain tissue were associated with lower seizure severity, suggesting that systemic and central exposure may contribute to the observed anticonvulsant effects, although additional pharmacodynamic and disease-related factors are likely involved. Notably, chronic CBD treatment was well tolerated, with no significant adverse effects on body weight, hematological parameters, or liver function. At the molecular level, gene expression analyses in the epileptogenic focus revealed that CBD effects are strongly context-dependent, with sex-specific baseline transcriptional states representing the principal determinant of responses to both seizures and treatment. Seizure stimulation induced selective gene-specific changes, particularly in *Trpv1* and *Slc29a1*, whereas chronic CBD treatment produced a significant main effect on *5-Htr1a*. Modulation of *Adora1*, *Cnr1*, and *Trpv1* was primarily driven by sex- and condition-dependent interactions rather than uniform treatment effects. Together, these findings indicate that chronic CBD treatment modulates seizure-related phenotypes and selected molecular pathways, the latter in a sex-dependent manner, and identify the inferior colliculus as a key neuroanatomical substrate for investigating CBD mechanisms in AGS models.

### 4.1 Pharmacokinetics and bioavailability of CBD in the GASH/Sal model

Interpretation of CBD pharmacokinetics across experimental studies is inherently constrained by differences in species, strain, formulation, analytical matrices (plasma vs. serum), and routes of administration. Within these limitations, the present data indicate that systemic exposure to CBD in the GASH/Sal hamster is markedly lower than that reported in other rodent models. After a single intraperitoneal administration of 200 mg/kg CBD, peak serum concentrations in non-stimulated GASH/Sal animals reached 384.6 ng/mL at 60 min. This exposure is substantially lower than plasma concentrations reported by Deiana et al. (2012) in rats (∼2.6 µg/mL) and mice (∼14.3 µg/mL) following a 120 mg/kg dose formulated in Cremophor® and administered via the same route. Thus, despite a 1.67-fold higher dose, systemic CBD levels in the GASH/Sal model were reduced by nearly one order of magnitude relative to rats and more than one order of magnitude relative to mice. Although the use of serum rather than plasma may contribute to lower measured CBD concentrations—given the drug loss during coagulation through adsorption to the fibrin clot and cellular components—this methodological difference is unlikely to fully explain the magnitude of the observed discrepancy. Instead, these findings point to pronounced species- or strain-specific differences in absorption and clearance. This interpretation is supported by prior work in the GASH/Sal model, in which a 100 mg/kg dose yielded peak serum concentrations of ∼263 ng/mL (Cabral-Pereira et al., 2021). Together with earlier reports of shortened elimination half-lives for multiple antiseizure medications in GASH/Sal animals (Barrera-Bailón et al., 2013), these data suggest enhanced metabolic clearance or altered drug disposition associated with this genetic epileptic phenotype. Reduced systemic exposure therefore provides a plausible pharmacokinetic explanation for the limited anticonvulsant efficacy observed at lower CBD doses in this model and supports the rationale for the higher dosing strategy used in the present study. The relevance of repeated administration is further underscored by comparison with the work of Do Val-da Silva et al. (2017) in a rat model of pilocarpine-induced temporal lobe epilepsy. In that study, plasma CBD was detectable shortly after a single 10 mg/kg injection but declined to low or undetectable levels at later time points, whereas repeated administration led to progressively higher plasma concentrations. Although direct quantitative comparison is not feasible due to major differences in dose, species, and sampling strategy, this pattern is consistent with our observation that sustained exposure in the GASH/Sal model requires repeated dosing. Quantification of CBD in brain tissue was essential to confirm central exposure, particularly given evidence that epilepsy may alter blood-brain barrier function (Marchi et al., 2012). Following acute administration, the brain-to-serum concentration ratio in GASH/Sal animals was approximately 2.1, indicating efficient penetration into brain tissue despite low circulating levels. This value closely aligns with the brain-to-plasma ratios reported by Deiana et al. (2012) in rats using a comparable formulation, suggesting that central distribution of CBD is not fundamentally impaired in the GASH/Sal model. Preserved blood-brain barrier permeability to CBD is consistent with recent findings by Zeballos et al. (2025), who reported no upregulation of cerebral P-glycoprotein (P-gp) in GASH/Sal animals following audiogenic kindling. Unlike other epilepsy models in which seizure activity induces P-gp overexpression and may restrict antiseizure drug penetration, P-gp levels in GASH/Sal animals remained stable or were slightly reduced in regions such as the inferior colliculus (Zeballos et al., 2025). This transporter profile may contribute to the maintained brain-to-serum CBD ratios observed in the present study, suggesting that chronic seizure activity in this model does not impose a P-gp–mediated barrier to CBD brain entry. While Deiana et al. (2012) reported a rapid decline in brain CBD concentrations after a single dose, the twice-daily administration over 14 days employed here resulted in substantially higher and sustained brain levels. When considered relative to the acute condition evaluated in females, chronic dosing resulted in comparable or higher mean brain CBD concentrations at the end of treatment, reaching 1,214 ± 524 ng/mL in males and 1835 ± 587 ng/mL in females. Although the difference between sexes was not statistically significant, these values are consistent with the accumulation of this highly lipophilic compound in brain tissue during repeated administration and suggest that chronic CBD dosing may help sustain central exposure despite limited systemic bioavailability. A relevant limitation of the present study is the absence of data on 7-hydroxy-cannabidiol, the primary active metabolite of CBD. This metabolite exhibits anticonvulsant activity, readily enters brain tissue, and may contribute substantially to therapeutic effects, potentially exceeding those of the parent compound in some seizure models (Rana et al., 2023). Clinical studies further demonstrate that exposure to this metabolite is influenced by concomitant antiseizure medications and displays marked inter-individual variability (Morrison et al., 2019; Johannessen Landmark et al., 2025). Neither the present work nor our previous studies in the GASH/Sal model evaluated the contribution of CBD metabolites, despite evidence that metabolite-mediated effects may occur independently of changes in parent-drug concentrations. Thus, future studies incorporating metabolite-specific pharmacokinetic and pharmacodynamic analyses in serum and brain tissue will therefore be necessary to fully characterize the mechanisms underlying CBD efficacy in this model. Finally, the inter-individual variability observed in serum and brain CBD concentrations in the GASH/Sal model is consistent with the established pharmacokinetic profile of CBD, including in humans, which is characterized by extensive tissue distribution, accumulation with repeated dosing, and pronounced variability (Millar et al., 2018). This variability likely reflects a combination of external and intrinsic factors, such as *ad libitum* feeding conditions and inter-individual differences in adipose tissue mass and metabolic capacity. Given its high lipophilicity, CBD can accumulate in adipose tissue and be released gradually, contributing to prolonged elimination and fluctuating systemic and brain levels. In line with these mechanisms, Millar et al. (2018) demonstrated a marked influence of fed versus fasted states on CBD bioavailability, supporting the possibility that dietary and physiological differences influenced CBD distribution and clearance in the present study. Despite limited systemic exposure in GASH/Sal hamsters, repeated administration of CBD at 200 mg/kg twice daily resulted in sustained brain accumulation. This supports the pharmacokinetic suitability of this regimen for exposure levels associated with anticonvulsant efficacy and underscores the need for dosing strategies adapted to the distinctive pharmacokinetic profile of this genetic epilepsy model.

### 4.2 Contextualizing the modulation of audiogenic seizures by CBD in the GASH/Sal model

Our study indicates that both acute and chronic intraperitoneal administration of CBD (200 mg/kg) significantly modulates AGS in the GASH/Sal model in a time-dependent manner, with a numerically greater response in females that did not reach statistical significance. This modulation was reflected in reduced seizure severity, prolonged latency to seizure onset, altered behavioral phase durations, and—particularly in females—complete seizure suppression in a substantial subset of animals. These effects align with extensive preclinical evidence supporting the anticonvulsant efficacy of CBD across a range of experimental epilepsy models. CBD has shown seizure-suppressing effects in rodent models of generalized seizures, such as maximal electroshock and pentylenetetrazol, as well as in focal and limbic seizure models, including the penicillin-induced partial seizure and pilocarpine-induced temporal lobe epilepsy models (Consroe and Wolkin, 1977; Jones et al., 2010; Jones et al., 2012). In AGS models, CBD reduced the severity and duration of midbrain-driven seizures in genetically epilepsy-prone rats (Lazarini-Lopes et al., 2023a) and attenuated seizure expression in Angelman syndrome mouse models (Gu et al., 2019). Importantly, these preclinical findings have translated into clinical success: randomized controlled trials have shown that purified CBD significantly reduces seizure frequency in patients with treatment-resistant epilepsies such as Dravet syndrome and Lennox–Gastaut syndrome (Devinsky et al., 2017; Thiele et al., 2018), leading to regulatory approval of CBD-based therapies. Therefore, this bench-to-bedside progression underscores the translational relevance of preclinical models like the GASH/Sal model for cannabinoid research. Building on prior work in the GASH/Sal model showing only mild effects at 100 mg/kg in males (Cabral-Pereira et al., 2021), we found that increasing the dose to 200 mg/kg produced significantly improved anticonvulsant outcomes. Notably, the neuroethological characterization closely paralleled the quantitative cSI analysis, indicating that the observed behavioral attenuation following CBD treatment was consistent with the statistically detected reductions in seizure severity. At the descriptive level, female animals exhibited more pronounced and rapid responses, with some achieving complete seizure suppression (cSI = 0) as early as 1 h post-injection. Males, in turn, required repeated dosing over 14 days to achieve more modest seizure control; these patterns reflect numerical trends not supported by significant male–female comparisons. This finding mirrors observations in a preclinical model of temporal lobe epilepsy, where oral CBD administration provided progressive seizure protection (Patra et al., 2019). Beyond overall reductions in seizure severity, CBD reshaped the temporal structure of seizure expression, delaying onset and redistributing the balance between seizure phases. Latency to seizure onset increased significantly in both sexes, with the within-sex effect reaching significance earlier in females (4 h) than in males (day 7); direct male–female comparisons, however, were not significant. The two principal seizure phases were also affected within the female group, where CBD significantly altered the wild running phase (transient lengthening at 1 h) and shortened the overall convulsive phase; no significant treatment effect on these phases was detected in males. Together, these dynamics suggest that CBD may partially decouple seizure progression, shifting expression away from the generalized convulsive component toward the less severe hypermotor (wild running) phase. Supporting this, a similar dissociation has been reported in other models, including febrile seizures, where CBD delayed seizure onset and reduced convulsive severity (Yu et al., 2020). Sex-based differences in seizure susceptibility and pharmacoresponsiveness are well established in both preclinical and clinical contexts, though their manifestations are not always clear (Christensen et al., 2005; Scharfman and MacLusky, 2014; Cepeda et al., 2022). However, despite these known disparities, most preclinical studies have historically relied on a single sex—often males—to reduce variability, thereby overlooking the biological relevance of sex as a modulator of CBD treatment response. Our study helps to address this gap by characterizing the response to CBD in both sexes of the GASH/Sal model. Descriptively, females tended to show faster, stronger, and more sustained anticonvulsant responses, including more frequent seizure suppression, whereas males tended to respond more slowly; importantly, these differences were numerical and did not reach significance in direct male–female comparisons. Because serum and brain CBD concentrations did not differ significantly between sexes, any contribution to this apparent (non-significant) sex difference would more plausibly reflect endogenous biological factors than differential drug exposure. Prior studies have shown that hormonal fluctuations during the estrous cycle can modulate neuronal excitability and seizure susceptibility (Nicoletti et al., 1985; Scharfman and MacLusky, 2006) and may therefore influence CBD responsiveness. Supporting this, Fabris et al. (2022) reported that CBD produces anxiolytic-like effects in both sexes, but females respond only during the late diestrus phase and at lower doses than males, suggesting sex- and hormone-dependent modulation of CBD effects. While we did not monitor the estrous cycle in female GASH/Sal animals—a limitation of this study—our findings nonetheless highlight the critical role of sex as a biological variable in preclinical epilepsy research. This perspective aligns with recent calls to address both the opportunities and the practical challenges of incorporating sex and hormonal status into experimental design (Christian-Hinman, 2024) and positions the GASH/Sal model as a useful platform for advancing more personalized epilepsy therapies.

### 4.3 Safety and tolerability of chronic CBD treatment in the GASH/Sal model

The evaluation of safety and tolerability is essential when considering any candidate for chronic antiseizure therapy. Our long-term administration of CBD (200 mg/kg, i.p., twice daily for 14 days) was well-tolerated, showing no evidence of systemic toxicity or adverse effects on hepatic or hematological function. Given that body weight is a sensitive indicator of general health and potential drug-related side effects, we closely monitored changes. Although previous studies in rats reported a reduction in body weight gain following a 14-day intraperitoneal CBD regimen (Ignatowska-Jankowska et al., 2011), our findings in the GASH/Sal model showed only a mild, non-significant attenuation of weight gain in males relative to sham controls and baseline, with no comparable trend in females. This male-predominant pattern cannot be attributed to differential drug exposure: serum and brain CBD concentrations were, if anything, numerically higher in females — consistent with sex-dependent CBD pharmacokinetics reported in rodents and with the more mixed evidence in humans (reviewed in Matheson et al., 2022) — so an exposure-driven effect would be expected to manifest in females rather than males. The dissociation instead points to sex-specific pharmacodynamic or metabolic factors, in line with evidence that CBD may modulate appetite, energy metabolism, or hypothalamic signaling in a concentration-dependent manner (Pinto and Martel, 2022; Rodrigues et al., 2023). Consistent with this, our earlier data using a lower CBD dose (100 mg/kg) likewise showed no impact on normal growth (Cabral-Pereira et al., 2021). Given its small magnitude and lack of statistical significance, this trend should be regarded as exploratory and warrants confirmation in adequately powered studies.

Given reports of neurotoxic effects at very high CBD concentrations in cell culture systems (Romariz et al., 2024), it is important to consider the potential neurological consequences of chronic administration in the present study, particularly as the dose used is substantially higher, on a mg/kg basis, than the approved dose in humans. Although dedicated neurotoxicity assays were not performed, our previous work also evaluated locomotor activity and emotionality following chronic CBD administration in the GASH/Sal model using the open-field test (Cabral-Pereira et al., 2021). CBD treatment resulted in reduced locomotor and exploratory activity, effects that were more pronounced when combined with valproic acid and are consistent with mild sedative properties previously described in other rodent epilepsy models (Pickens, 1981). Importantly, these behavioral changes were not accompanied by signs of motor deterioration, abnormal neurological presentation, or behavioral patterns suggestive of toxic encephalopathy. Furthermore, in the present study, seizure severity assessments did not reveal paradoxical worsening, atypical seizure phenotypes, or progressive behavioral impairment during the chronic treatment period. Together, these findings argue against overt neurotoxic effects under the experimental conditions employed. Nevertheless, given the relatively high dose required to achieve adequate systemic exposure in this strain and known interspecies pharmacokinetic differences, these findings should not be directly extrapolated to clinical contexts, and the potential neurological risks associated with high systemic CBD exposures warrant continued investigation.

One of the most clinically relevant concerns associated with CBD therapy is hepatotoxicity, especially when used in combination with other antiseizure medications such as valproate. Clinical studies have reported elevated liver transaminases in 5%–20% of epileptic patients receiving CBD alongside valproic acid (Devinsky et al., 2017; 2018; Thiele et al., 2018), and elevations in liver enzymes have also been described in healthy volunteers receiving short-term CBD monotherapy under controlled clinical conditions (Watkins et al., 2021; Florian et al., 2025). Preclinical studies in rodents and non-human primates have further shown dose-dependent increases in hepatic enzymes and liver weight at high doses (300 mg/kg), albeit without overt functional impairment (Rosenkrantz et al., 1981; Ewing et al., 2019). In the present study, chronic administration of CBD at 200 mg/kg did not produce significant alterations in standard serum markers of liver function (AST, ALT, bilirubin, albumin or total proteins) in either sex. These findings are consistent with our previous observations in the GASH/Sal model using a lower CBD dose, including when co-administered with valproic acid (Cabral-Pereira et al., 2021). Importantly, these results should be interpreted as indicating an absence of detectable hepatotoxicity under the specific experimental conditions and endpoints assessed, rather than as evidence of clinical hepatic safety. It is possible that the GASH/Sal model lacks sensitivity to detect subtle or delayed forms of drug-induced liver injury, or that species-specific differences in CBD metabolism limit direct translational inference. Accordingly, while our findings suggest that the dosing regimens used were well tolerated in this model, they do not exclude the potential for hepatotoxicity in humans, particularly under conditions of prolonged exposure, higher systemic levels, or pharmacokinetic interactions with concomitant medications. Hematological profiles remained within physiological ranges across all groups, except for a significant increase in platelet counts in both CBD-treated males and females. Although the biological relevance of this increase is uncertain and consistent with previous observations in our laboratory (Cabral-Pereira et al., 2021), clinical studies have reported the opposite effect. Notably, thrombocytopenia has been described in pediatric epilepsy patients receiving CBD in combination with valproic acid (McNamara et al., 2020), suggesting that drug interactions or patient-specific factors may influence platelet regulation. The discrepancy between these clinical observations and our preclinical findings may reflect species differences or model-specific responses. In addition, white blood cell counts were significantly reduced in CBD-treated females relative to sham controls, an effect not observed in males, whereas red blood cell counts, hemoglobin, and hematocrit were unchanged across all groups. This reduction in white blood cells is consistent with the immunomodulatory profile of CBD, which has been reported to suppress leukocyte proliferation and promote predominantly anti-inflammatory and immunosuppressive responses across several experimental systems (Nichols and Kaplan, 2020).

A methodological consideration in this study relates to the use of repeated intraperitoneal injections every 12 h for 14 days. Although widely used for systemic drug delivery in rodents, this route of administration carries risks such as variability in absorption, local tissue irritation, and stress from frequent handling (Arioli and Rossi, 1970; Claassen, 1994). Given that behavioral stress can confound outcomes in sex-difference research on epilepsy (Scharfman and MacLusky, 2014), we minimized procedural distress by performing all injections under light isoflurane anesthesia. This refinement adheres to the 3Rs framework and is particularly important for the GASH/Sal strain, which is both seizure-prone and behaviorally sensitive. All groups, including controls, were subjected to the same anesthetic protocol and handling schedule, ensuring methodological consistency and preventing procedural bias. Previous studies have reported interactions between cannabinoids and anesthetic agents, including modulation of isoflurane induction and recovery dynamics, primarily in the context of CBD premedication under sustained anesthetic exposure (Uršič et al., 2022). In contrast, in the present study isoflurane exposure was restricted to brief anesthesia during intraperitoneal administration and blood sample collection, and was not used at any other stage of the experimental procedure, including seizure testing, thereby making a meaningful pharmacodynamic interaction unlikely. Although transient effects of isoflurane on hepatic enzyme activity have been reported (Li et al., 2021), no such alterations were observed in our biochemical analyses, supporting the reliability of this refined protocol. An additional consideration is the use of Cremophor RH 40 as the formulation vehicle. Although this hydrogenated castor oil derivative has a substantially more favorable safety profile than the structurally related Cremophor EL, non-ionic surfactants of this class are not biologically inert and have been associated with complement activation and hematological alterations (Gelderblom et al., 2001). Nonetheless, because all groups — including sham controls — received identical volumes of vehicle under the same administration protocol, any vehicle-mediated effects would be expected to affect groups equally, preserving the internal validity of between-group comparisons. Furthermore, stable body weight, normal hematological values, and unchanged liver enzyme levels indicate that chronic high-dose CBD was well tolerated via this administration route. Beyond safety, pharmacokinetic analyses confirmed consistent systemic and central CBD exposure, with differences in exposure-response relationships across models (Devinsky et al., 2014; Lazarini-Lopes et al., 2020; Lazarini-Lopes et al., 2023a). Together, these results support a refined and safe protocol for repeated CBD administration and highlight the translational relevance of the GASH/Sal model for long-term cannabinoid therapy studies.

### 4.4 Coordinated transcriptional regulation in the inferior colliculus is dominated by sex, with selective modulation by CBD and seizure induction

The transcriptional landscape of the inferior colliculus in the GASH/Sal model is not a passive substrate uniformly remodeled by pharmacological or seizure stimulation condition. Our data reveal instead a hierarchically organized molecular environment, in which biological sex constitutes the dominant regulatory axis, whereas chronic CBD treatment and acoustic seizure stimulation act as secondary modulators whose impact is conditional on this underlying architecture. This hierarchy carries direct implications for how anticonvulsant therapies should be conceptualized, evaluated and ultimately translated. One intriguing pattern arising from our dataset is an apparent paradox: the numerically greater female behavioral response, if confirmed in adequately powered studies, would sit alongside their significantly lower inhibitory transcript levels. Under a linear interpretation of transcript abundance as functional reserve, this configuration is counterintuitive — the 5-HT1A receptor (Salgado-Commissariat and Alkadhi, 1997; Gariboldi et al., 1996; Sarnyai et al., 2000; Bagdy et al., 2007), A1 adenosine receptor (During and Spencer, 1992; Boison, 2016; Weltha et al., 2019), CB1 receptor (Karlócai et al., 2011) and Sigma-1 receptor (Ryskamp et al., 2019) are all established mediators of seizure suppression, and their lower expression in the more seizure-resistant sex demands explanation. We propose that the resolution lies in reframing transcript abundance as an indicator of regulatory state rather than functional capacity. Within this framework, the elevated baseline of inhibitory transcripts in males may reflect a transcriptional environment under chronic compensatory load — a system already mobilized to counterbalance ongoing seizure susceptibility and consequently approaching the upper limit of its dynamic range. The lower female baseline, by contrast, is most parsimoniously interpreted as a homeostatically buffered configuration that retains responsive bandwidth and can therefore engage adaptive transcriptional programs when challenged. This conceptualization aligns with broader principles of allostatic load in chronic neurological disease, in which apparently elevated expression of protective mediators paradoxically reflects pathway exhaustion rather than reserve (McEwen, 2004; Vezzani et al., 2019).

This reframing is empirically grounded in the post-hoc structure of our data, which constitutes the strongest single signature of the dataset and has not been previously described in this model. Female animals retained relative transcriptional stability across experimental conditions for all six genes analyzed, whereas males exhibited reproducible destabilization under combined pharmacological and seizure induction. We interpret this asymmetry as evidence that the apparently uniform Sex × Treatment interactions detected for *Cnr1*, *Adora1* and *5-Htr1a* are not driven by enhancement in females but by destabilization in males — a distinction that fundamentally changes the mechanistic narrative. The dominant transcriptional event is therefore not what CBD does to the female brain, but what it fails to do to the male brain when seizures occur. In males, the combination of chronic CBD and seizure stimulation coordinately suppresses the very pathways through which CBD’s anticonvulsant action is presumed to be exerted, producing a molecular configuration that is the opposite of therapeutically desirable. This interpretation is concordant with previous work documenting CBD-induced *Slc29a1* downregulation in male animals (Cabral-Pereira et al., 2021) and with the established role of *Cnr1* in the pro-epileptogenic environment of the GASH/Sal inferior colliculus (Fuerte-Hortigón et al., 2021), and provides a parsimonious molecular rationale for the limited and delayed behavioral response previously reported in male animals of this strain.

The serotonergic system emerges as the principal transcriptional target of chronic CBD in the inferior colliculus, whereas no other neuromodulatory pathway showed a comparable treatment-related response. This selective effect suggests that CBD does not broadly alter transcriptional activity in this region, but instead modulates a restricted set of molecular targets, consistent with its pharmacological profile and the proposed role of 5-HT1A signaling in its anticonvulsant effects. Importantly, this modulation appeared to depend on the experimental condition. The Treatment × Condition interaction governing *5-Htr1a* indicates that CBD’s transcriptional impact on the serotonergic system is contingent on whether seizure activity has occurred, with the failure of CBD to sustain serotonergic transcription specifically in stimulated males representing a key molecular limitation on therapeutic efficacy. This finding has direct translational implications. State-dependent pharmacology — in which a drug’s transcriptional fingerprint is determined by the activity state of the target tissue — implies that drug efficacy cannot be reliably predicted from baseline tissue characterization alone and must be evaluated under the pathophysiological conditions in which the drug is intended to act. For CBD specifically, this argues that meaningful preclinical assessment of serotonergic engagement requires post-ictal sampling rather than steady-state evaluation in unstimulated animals.

Seizure-related transcriptional changes in our dataset were gene-selective rather than widespread, and several of these effects differed between sexes. Among them, the sex-specific regulation of *Slc29a1* may be particularly relevant from a mechanistic perspective. Equilibrative nucleoside transporter 1 sets the gain of A1 receptor-mediated tonic inhibition by governing extracellular adenosine availability (Boison, 2008; Hamil et al., 2012), and a sex-divergent ENT1 response to seizure activity therefore implies that the adenosinergic anticonvulsant brake is recruited differently in males and females during and after seizure events. Consequently, these findings suggest that adenosine homeostasis may not respond uniformly across sexes and support the idea that adenosinergic approaches for seizure control could benefit from sex-stratified evaluation.

The contextual integration of *Trpv1* regulation, captured by a three-way Sex × Treatment × Condition interaction, deserves particular interpretive care because the directionality of CBD’s effect determines whether this finding is read as adaptive or maladaptive. Vehicle-treated stimulated males displayed the most suppressed *Trpv1* expression of the entire dataset, and CBD restored expression toward baseline rather than driving a paradoxical pro-excitatory upregulation above it. This distinction may be also mechanistically relevant. While *Trpv1* upregulation has been associated with enhanced excitability in temporal lobe epilepsy and acoustic seizure models (Sun et al., 2013; Nazıroğlu and Övey, 2015; Lazarini-Lopes et al., 2022), TRPV1 is also a direct CBD target whose sustained engagement typically results in functional desensitization. The reversal of stimulation-induced *Trpv1* suppression by CBD is therefore most coherently interpreted as a recalibration of vanilloid signaling toward homeostatic baseline rather than as a destabilizing pro-excitatory event. The contrast with the destabilization observed for *Cnr1*, *Adora1* and *Slc29a1* in the same animals is instructive: CBD’s transcriptional effects are not uniformly disruptive in males but are pathway-specific, with some targets normalized and others suppressed below baseline. This dissociation argues against a single explanatory mechanism for sex-dependent CBD efficacy and instead points to a pathway-by-pathway accounting in which the net therapeutic outcome reflects the algebraic sum of normalizing and destabilizing effects across multiple molecular axes.

Several limitations frame the scope of these inferences. Whole-tissue mRNA quantification cannot disentangle neuronal from glial contributions, a distinction of growing importance given the recognized roles of microglia and astrocytes in mediating CBD’s anti-inflammatory and neuroprotective actions (Scarante et al., 2021; Landucci et al., 2022) and the documented sex-dependent differences in neuron-to-glia ratios across brain regions (Autio-Kimura et al., 2024). Some fraction of the dimorphism reported here may therefore reflect differential cellular composition rather than differential per-cell expression, a question that single-cell or cell-type-resolved transcriptomic approaches are well positioned to address. Transcript abundance does not translate linearly into protein availability or functional receptor signaling, and protein-level validation will be required before transcriptional signatures can be used as predictive biomarkers of therapeutic response. The single post-treatment sampling window does not capture temporal dynamics, leaving open whether male transcriptional destabilization represents a transient perturbation or a sustained reconfiguration. Finally, the absence of circulating sex steroid measurements means that the hormonal mediation of the dimorphism we describe remains inferred rather than demonstrated; estrous cycle-resolved or gonadectomy-controlled designs will be necessary to causally implicate sex hormone signaling in the molecular phenotype.

The broader implication of these findings might extend beyond the GASH/Sal model. Our findings suggest that pooling sexes or relying primarily on male animals in preclinical antiepileptic drug evaluation may overlook a key dimension of biological variability, given that sex appears to operate as a major organizing principle—rather than an additive variable—of the transcriptional response to both seizures and pharmacological intervention. The molecular signature of CBD efficacy is not encoded in any single pathway but in the stability of the inhibitory transcriptome under combined pharmacological and ictogenic challenge — a property that is preserved in females and disrupted in males in this model. If this principle generalizes to other genetic and acquired epilepsies, it implies that meaningful translation of CBD and other anticonvulsants will depend on sex-stratified preclinical evaluation, on assessment under post-ictal rather than baseline conditions, and on a shift from pathway-centric to system-stability-centric biomarkers of therapeutic response. The path toward precision anticonvulsant therapy is unlikely to run through the identification of a single dominant target; it will more probably require integrated readouts of how sex-specific molecular environments respond, collectively, to the therapeutic challenge.

## Data Availability

The original contributions presented in the study are included in the article/Supplementary Material, further inquiries can be directed to the corresponding author.
