# Supplementary material for: Anticonvulsant effects, safety, and sex-dependent molecular signatures of cannabidiol treatment in a preclinical model of audiogenic epilepsy
Source: Front Pharmacol. 2026 Aug 7;17:1745696. doi: 10.3389/fphar.2026.1745696 (PMC13493383; doi:10.3389/fphar.2026.1745696)
Supplement: Supplementary file 1 [file DataSheet1.pdf]

Supplementary Material 1. List of oligonucleotide primers used for RT-qPCRs.

Gen target	ID Transcript Ensembl *Mesocricetus auratus* [a]	Primer forward	Primer reverse	Size of products (bp)	E^b (%)
Trpv1	*ENSMAUG00000008181*	GACGAGGGTGAACTGGACTACC	CTTGACACCCTCACAGTTGC	60	2.16
5-Htr1a	*ENSMAUG00000007970*	CACCATCAGCAAGGATCACG	AGAGCAGCAGCGGGATATAGA	85	2.03
Sigmar1	*ENSMAUG00000011503*	AGAGAGGGCACCACGAAAAG	AAGGAGCGGAGGGTATAGAAGA	88	1.97
Adora1	*ENSMAUG00000018351*	CATCGTATCCCTGGCGGTAG	ACGCAGGTGTGGAAGTAGGTCT	75	2.01
Slc29a1	*ENSMAUG00000006341*	CATCAGGAGGTGTGTGGGTTT	TCATGGTTCCAGGGTTCTCG	162	2.07
Cnr1	*ENSMAUG00000014040*	TGTTGACTTCCATGTGTTCCA	GGTCTGGTGACGATCCTCTT	126	1.98
Actb	*ENSMAUG00000008763*	AGCCATGTACGTAGCCATCC	ACCCTCATAGATGGGCACAG	105	2.03

"a" Indicates the identifier (ID) of each gene in the corresponding Ensembl sequences of the Syrian hamster (*M. auratus*). "b" Indicates percentage of qPCR primer efficiency (E). Primer efficiency was calculated according to the following equation: $E = [10^{[-1/slope]} - 1] \times 100$
