# Supplementary material for: Anticonvulsant effects, safety, and sex-dependent molecular signatures of cannabidiol treatment in a preclinical model of audiogenic epilepsy
Source: Front Pharmacol. 2026 Aug 7;17:1745696. doi: 10.3389/fphar.2026.1745696 (PMC13493383; doi:10.3389/fphar.2026.1745696)
Supplement: Supplementary file 2 [file DataSheet2.pdf]

Supplementary Material 2. Full statistical summary of the categorized seizure index (cSI).

Seizure severity was scored on an ordinal scale from 0 (no seizure) to 8 (maximal generalized seizure) and assessed repeatedly in the same animals at five time points in a 2 × 2 × 5 mixed factorial design (treatment and sex as between-subject factors; time as a within-subject factor; n = 6/8/6/8 per group). All analyses were rank-based and non-parametric. Data are summarized as median [interquartile range].

A. Descriptive statistics — median [IQR] of cSI by group and time point

Group	Pre-treatment	1 h	4 h	7 d	14 d
Sham · Male (n=6)	8 [8–8]	8 [8–8]	8 [8–8]	8 [8–8]	8 [8–8]
Sham · Female (n=6)	8 [8–8]	8 [8–8]	8 [8–8]	8 [8–8]	8 [8–8]
CBD · Male (n=8)	8 [8–8]	7 [6.5–7.25]	8 [8–8]	6.5 [4.5–8]	5 [2.75–8]
CBD · Female (n=8)	8 [8–8]	2.5 [2–8]	5.5 [1.5–8]	3.5 [0–7]	2 [0–4.25]

B. Global analysis — nonparametric factorial test for longitudinal data (nparLD, F2-LD-F1 design)

Effect	ATS	df	P	Sig.
Treatment	22.61	1	<0.001	****
Sex	4.15	1	0.042	*
Time	8.78	2.66	<0.001	****
Treatment × Sex	0.34	1	0.561	ns
Treatment × Time	8.78	2.66	<0.001	****
Sex × Time	0.87	2.66	0.446	ns
Treatment × Sex × Time	0.87	2.66	0.446	ns

C. Cannabidiol versus sham at each time point (Mann–Whitney U test)

Time	n (CBD / sham)	W	r	P	P (Holm)	Sig.
Pre-treatment	16 / 12	104	0.08	0.279	0.279	ns
1 h	16 / 12	34.5	−0.64	0.0016	0.0063	**
4 h	16 / 12	71.5	−0.26	0.120	0.241	ns
7 d	16 / 12	33.5	−0.65	0.0014	0.0063	**
14 d	16 / 12	33	−0.66	0.0013	0.0063	**

D. Within-cannabidiol time course

Overall effect of time (Friedman test): $\chi^2(4) = 19.00$, $P < 0.001$.

Each post-treatment time point versus the animal's own baseline (Wilcoxon signed-rank test):

Comparison	n	V	Median change	P	P (Holm)	Sig.
1 h vs baseline	11	0	−1	0.0036	0.014	*

Comparison	n	V	Median change	P	P (Holm)	Sig.
4 h vs baseline	5	0	0	0.057	0.057	ns
7 d vs baseline	11	0	−3	0.0037	0.014	*
14 d vs baseline	11	0	−5	0.0035	0.014	*

E. Sex comparison within the cannabidiol group (male versus female) at each time point (Mann–Whitney U test)

Time	n (M / F)	W	r	P	P (Holm)	Sig.
Pre-treatment	8 / 8	32	0.00	—	—	—
1 h	8 / 8	43	0.34	0.258	0.477	ns
4 h	8 / 8	44.5	0.39	0.125	0.477	ns
7 d	8 / 8	47	0.47	0.119	0.477	ns
14 d	8 / 8	43.5	0.36	0.234	0.477	ns

Notes

Abbreviations: ATS, ANOVA-type statistic (nparLD); IQR, interquartile range; r, rank-biserial correlation (effect size); Sig., significance; V and W, Wilcoxon signed-rank and Wilcoxon rank-sum test statistics, respectively.

Panel A is descriptive and contains no statistical test.
Panels C and D pool males and females (cannabidiol, n = 16; sham, n = 12), whereas Panel E reports the sexes separately (n = 8 per sex within the cannabidiol group). The sexes were pooled in Panels C and D because neither the treatment × sex interaction (Panel B) nor any male-versus-female comparison (Panel E) was significant.

*P values within each family of pairwise comparisons (Panels C, D and E) were adjusted for multiple testing using the Holm procedure; P (Holm) denotes the adjusted value. All tests were two-tailed and statistical significance was set at P < 0.05. Sig., significance: *P < 0.05, **P < 0.01, ***P < 0.001, ****P < 0.0001; ns, non-significant (P ≥ 0.05); —, not estimable. Markers reflect the Holm-adjusted P in Panels C–E and the ATS P in Panel B.*

The Mann–Whitney U and Wilcoxon rank-sum tests are equivalent; the statistic is reported as W, as returned by R's wilcox.test function. Non-integer degrees of freedom for the within-subject (time) effects in Panel B reflect the Box-type approximation used by nparLD.

In Panel C, a negative r indicates lower seizure severity in the cannabidiol group relative to sham. In Panel E, a positive r indicates higher severity (i.e., a weaker response) in males; the pre-treatment comparison was not estimable (—) because all animals were at the ceiling of the scale.

In Panel D, n is the number of cannabidiol-treated animals with a non-zero change from baseline at each time point: animals that remained at the maximal baseline score (cSI = 8) yield tied pairs that the Wilcoxon signed-rank test discards, so n is below the full group size of 16. For the same reason the positive-rank sum (V) equals 0 at every time point — because all animals started at the ceiling, any within-animal change could only be a reduction — and the median change in cSI is reported to convey the magnitude and direction of the effect.

A cumulative-link mixed-effects (ordinal) model was not used: sham-treated animals remained uniformly at the ceiling of the scale, producing quasi-complete separation and a singular Hessian (a non-identifiable model). Rank-based methods were therefore adopted for all analyses.

Analyses were performed in R v4.3.3 using the nparLD package (F2-LD-F1 design) and base-R functions.

F. Raw data for categorized seizure index (cSI).

Animal	Sex	Treatment	Time	Categorized seizure index (cSI)
Sham_S_M_1	M	Sham	Pre-treatment	8
Sham_S_M_1	M	Sham	1 h	8
Sham_S_M_1	M	Sham	4 h	8
Sham_S_M_1	M	Sham	7 d	8
Sham_S_M_1	M	Sham	14 d	8
Sham_S_M_2	M	Sham	Pre-treatment	8
Sham_S_M_2	M	Sham	1 h	8
Sham_S_M_2	M	Sham	4 h	8
Sham_S_M_2	M	Sham	7 d	8
Sham_S_M_2	M	Sham	14 d	8
Sham_S_M_3	M	Sham	Pre-treatment	8
Sham_S_M_3	M	Sham	1 h	8
Sham_S_M_3	M	Sham	4 h	8
Sham_S_M_3	M	Sham	7 d	8
Sham_S_M_3	M	Sham	14 d	8
Sham_S_M_4	M	Sham	Pre-treatment	8
Sham_S_M_4	M	Sham	1 h	8
Sham_S_M_4	M	Sham	4 h	8
Sham_S_M_4	M	Sham	7 d	8
Sham_S_M_4	M	Sham	14 d	8
Sham_S_M_5	M	Sham	Pre-treatment	8
Sham_S_M_5	M	Sham	1 h	8
Sham_S_M_5	M	Sham	4 h	8
Sham_S_M_5	M	Sham	7 d	8
Sham_S_M_5	M	Sham	14 d	8
Sham_S_M_6	M	Sham	Pre-treatment	8
Sham_S_M_6	M	Sham	1 h	8
Sham_S_M_6	M	Sham	4 h	8
Sham_S_M_6	M	Sham	7 d	8
Sham_S_M_6	M	Sham	14 d	8
CBD_S_M_1	M	CBD	Pre-treatment	8
CBD_S_M_1	M	CBD	1 h	5
CBD_S_M_1	M	CBD	4 h	8
CBD_S_M_1	M	CBD	7 d	5
CBD_S_M_1	M	CBD	14 d	7
CBD_S_M_2	M	CBD	Pre-treatment	8
CBD_S_M_2	M	CBD	1 h	7
CBD_S_M_2	M	CBD	4 h	8
CBD_S_M_2	M	CBD	7 d	3

**CBD_S_M_2**	M	CBD	14 d	3
**CBD_S_M_3**	M	CBD	Pre-treatment	8
**CBD_S_M_3**	M	CBD	1 h	8
**CBD_S_M_3**	M	CBD	4 h	8
**CBD_S_M_3**	M	CBD	7 d	8
**CBD_S_M_3**	M	CBD	14 d	3
**CBD_S_M_4**	M	CBD	Pre-treatment	8
**CBD_S_M_4**	M	CBD	1 h	5
**CBD_S_M_4**	M	CBD	4 h	2
**CBD_S_M_4**	M	CBD	7 d	3
**CBD_S_M_4**	M	CBD	14 d	8
**CBD_S_M_5**	M	CBD	Pre-treatment	8
**CBD_S_M_5**	M	CBD	1 h	7
**CBD_S_M_5**	M	CBD	4 h	8
**CBD_S_M_5**	M	CBD	7 d	8
**CBD_S_M_5**	M	CBD	14 d	8
**CBD_S_M_6**	M	CBD	Pre-treatment	8
**CBD_S_M_6**	M	CBD	1 h	7
**CBD_S_M_6**	M	CBD	4 h	8
**CBD_S_M_6**	M	CBD	7 d	5
**CBD_S_M_6**	M	CBD	14 d	8
**CBD_S_M_7**	M	CBD	Pre-treatment	8
**CBD_S_M_7**	M	CBD	1 h	8
**CBD_S_M_7**	M	CBD	4 h	8
**CBD_S_M_7**	M	CBD	7 d	8
**CBD_S_M_7**	M	CBD	14 d	2
**CBD_S_M_8**	M	CBD	Pre-treatment	8
**CBD_S_M_8**	M	CBD	1 h	7
**CBD_S_M_8**	M	CBD	4 h	8
**CBD_S_M_8**	M	CBD	7 d	8
**CBD_S_M_8**	M	CBD	14 d	0
**Sham_S_F_1**	F	Sham	Pre-treatment	8
**Sham_S_F_1**	F	Sham	1 h	8
**Sham_S_F_1**	F	Sham	4 h	8
**Sham_S_F_1**	F	Sham	7 d	8
**Sham_S_F_1**	F	Sham	14 d	8
**Sham_S_F_2**	F	Sham	Pre-treatment	8
**Sham_S_F_2**	F	Sham	1 h	8
**Sham_S_F_2**	F	Sham	4 h	8
**Sham_S_F_2**	F	Sham	7 d	8
**Sham_S_F_2**	F	Sham	14 d	8
**Sham_S_F_3**	F	Sham	Pre-treatment	7
**Sham_S_F_3**	F	Sham	1 h	7

**Sham_S_F_3**	F	Sham	4 h	7
**Sham_S_F_3**	F	Sham	7 d	7
**Sham_S_F_3**	F	Sham	14 d	7
**Sham_S_F_4**	F	Sham	Pre-treatment	8
**Sham_S_F_4**	F	Sham	1 h	8
**Sham_S_F_4**	F	Sham	4 h	8
**Sham_S_F_4**	F	Sham	7 d	8
**Sham_S_F_4**	F	Sham	14 d	8
**Sham_S_F_5**	F	Sham	Pre-treatment	8
**Sham_S_F_5**	F	Sham	1 h	8
**Sham_S_F_5**	F	Sham	4 h	8
**Sham_S_F_5**	F	Sham	7 d	8
**Sham_S_F_5**	F	Sham	14 d	8
**Sham_S_F_6**	F	Sham	Pre-treatment	8
**Sham_S_F_6**	F	Sham	1 h	8
**Sham_S_F_6**	F	Sham	4 h	8
**Sham_S_F_6**	F	Sham	7 d	8
**Sham_S_F_6**	F	Sham	14 d	8
**CBD_S_F_1**	F	CBD	Pre-treatment	8
**CBD_S_F_1**	F	CBD	1 h	3
**CBD_S_F_1**	F	CBD	4 h	0
**CBD_S_F_1**	F	CBD	7 d	7
**CBD_S_F_1**	F	CBD	14 d	0
**CBD_S_F_2**	F	CBD	Pre-treatment	8
**CBD_S_F_2**	F	CBD	1 h	8
**CBD_S_F_2**	F	CBD	4 h	8
**CBD_S_F_2**	F	CBD	7 d	0
**CBD_S_F_2**	F	CBD	14 d	2
**CBD_S_F_3**	F	CBD	Pre-treatment	8
**CBD_S_F_3**	F	CBD	1 h	8
**CBD_S_F_3**	F	CBD	4 h	8
**CBD_S_F_3**	F	CBD	7 d	8
**CBD_S_F_3**	F	CBD	14 d	0
**CBD_S_F_4**	F	CBD	Pre-treatment	8
**CBD_S_F_4**	F	CBD	1 h	8
**CBD_S_F_4**	F	CBD	4 h	8
**CBD_S_F_4**	F	CBD	7 d	0
**CBD_S_F_4**	F	CBD	14 d	8
**CBD_S_F_5**	F	CBD	Pre-treatment	8
**CBD_S_F_5**	F	CBD	1 h	2
**CBD_S_F_5**	F	CBD	4 h	2
**CBD_S_F_5**	F	CBD	7 d	0
**CBD_S_F_5**	F	CBD	14 d	2

**CBD_S_F_6**	F	CBD	Pre-treatment	8
**CBD_S_F_6**	F	CBD	1 h	2
**CBD_S_F_6**	F	CBD	4 h	3
**CBD_S_F_6**	F	CBD	7 d	4
**CBD_S_F_6**	F	CBD	14 d	3
**CBD_S_F_7**	F	CBD	Pre-treatment	8
**CBD_S_F_7**	F	CBD	1 h	2
**CBD_S_F_7**	F	CBD	4 h	8
**CBD_S_F_7**	F	CBD	7 d	7
**CBD_S_F_7**	F	CBD	14 d	8
**CBD_S_F_8**	F	CBD	Pre-treatment	8
**CBD_S_F_8**	F	CBD	1 h	0
**CBD_S_F_8**	F	CBD	4 h	0
**CBD_S_F_8**	F	CBD	7 d	3
**CBD_S_F_8**	F	CBD	14 d	0
