# Supplementary material for: Anticonvulsant effects, safety, and sex-dependent molecular signatures of cannabidiol treatment in a preclinical model of audiogenic epilepsy
Source: Front Pharmacol. 2026 Aug 7;17:1745696. doi: 10.3389/fphar.2026.1745696 (PMC13493383; doi:10.3389/fphar.2026.1745696)
Supplement: Supplementary file 3 [file DataSheet3.pdf]

Supplementary Material 3. Full statistical summary of the latency, wild-running duration, and convulsive-phase duration.

Analysis: Mixed-effects model (Restricted Maximum Likelihood, REML) followed by Sidak’s multiple comparisons test.

A) Comparisons for seizure latency:

Seizure latency: Sham vs CBD (within the male group)

Table Analyzed	Seizure latency Sham vs CBD males
**Mixed-effects model (REML)**	Matching: Stacked
Assume sphericity?	No
Alpha	0,05

Fixed effects (type III)	P value	P value summary	Statistically significant (P < 0,05)?	F (DFn, DFd)	Geisser-Greenhouse's epsilon
Time	**0,0191**	*	**Yes**	**F (2,876, 27,32) = 3,977**	0,719
Treatment	0,0531	ns	No	F (1, 10) = 4,806	
Time x Treatment	**0,0265**	*	**Yes**	**F (4, 38) = 3,102**	

Compare each cell mean with the other cell mean in that row	Multiple comparation
Number of families	1
Number of comparisons per family	5
**Alpha**	**0,05**

Sidak's multiple comparisons test	Mean Diff,	95,00% CI of diff,	Significant?	Summary	Adjusted P Value
Sham_S_M - CBD_S_M					
Pre-treatment	0,125	-0,6645 to 0,9145	No	ns	0,9895
1 h	-0,75	-2,339 to 0,8395	No	ns	0,5987
4 h	-2,125	-4,678 to 0,4279	No	ns	0,1167
**7 (days)**	**-2,5**	**-4,470 to -0,5295**	**Yes**	*	**0,013**
14 (days)	0,5	-3,761 to 4,761	No	ns	0,9949

Test details	Mean 1	Mean 2	Mean Diff,	SE of diff,	N1	N2	t	DF
Sham_S_M - CBD_S_M	2	1,875	0,125	0,2266	4	8	0,5517	7
Pre-treatment	2,5	3,25	-0,75	0,503	4	8	1,491	9,959
1 h	2,5	4,625	-2,125	0,7854	4	8	2,706	8,854
4 h	2,25	4,75	-2,5	0,6124	4	8	4,082	9,22
7 (days)	4	3,5	0,5	1,041	4	6	0,4804	4,81
14 (days)								

Seizure latency: Sham vs CBD (within the female group)

Table Analyzed	Seizure latency Sham vs CBD females
**Mixed-effects model (REML)**	Matching: Stacked
Assume sphericity?	No
Alpha	0,05

Fixed effects (type III)	P value	P value summary	Statistically significant (P < 0,05)?	F (DFn, DFd)	Geisser-Greenhouse's epsilon
Time	0,0586	ns	No	F (1,327, 16,91) = 3,791	0,3317
Treatment	**0,0011**	**	**Yes**	**F (1, 51) = 12,03**	
Time x Treatment	0,3665	ns	No	F (4, 51) = 1,100	

Compare each cell mean with the other cell mean in that row	Multiple comparation
Number of families	1
Number of comparisons per family	5
**Alpha**	**0,05**

Sidak's multiple comparisons test	Mean Diff,	95,00% CI of diff,	Significant?	Summary	Adjusted P Value
Sham_S_F - CBD_S_F					
Pre-treatment	-0,125	-1,454 to 1,204	No	ns	0,9995
1 h	-1,071	-2,627 to 0,4842	No	ns	0,2522
**4 h**	**-2,5**	**-4,938 to -0,06154**	**Yes**	*	**0,0448**
7 (days)	-1,367	-2,972 to 0,2387	No	ns	0,1043
14 (days)	-1,433	-7,113 to 4,247	No	ns	0,9166

Test details	Mean 1	Mean 2	Mean Diff,	SE of diff,	N1	N2	t	DF
Sham_S_F - CBD_S_F								
Pre-treatment	2	2,125	-0,125	0,4352	6	8	0,2872	11,8
1 h	2,5	3,571	-1,071	0,5027	6	7	2,131	11
4 h	2,5	5	-2,5	0,6708	6	6	3,727	6,231
7 (days)	2,833	4,2	-1,367	0,4842	6	5	2,823	8,223
14 (days)	3,4	4,833	-1,433	1,556	5	6	0,9212	6,164

Seizure latency: female vs male (within the Sham group)

Table Analyzed	Seizure latency females vs males Sham
Mixed-effects model (REML)	Matching: Stacked
Assume sphericity?	No
Alpha	0,05

Fixed effects (type III)	P value	P value summary	Statistically significant (P < 0,05)?	F (DFn, DFd)	Geisser-Greenhouse's epsilon
**Time**	**0,0099**	**	**Yes**	**F (2,155, 16,70) = 5,976**	0,5387
Sex	0,9935	ns	No	F (1, 8) = 7,077e-005	
Time x Sex	0,6198	ns	No	F (4, 31) = 0,6670	

Compare each cell mean with the other cell mean in that row	Multiple comparation
Number of families	1
Number of comparisons per family	5
**Alpha**	**0,05**

Sidak's multiple comparisons test	Mean Diff,	95,00% CI of diff,	Significant?	Summary	Adjusted P Value
Sham_S_M - Sham_S_F					
Pre-treatment	0	-1,036 to 1,036	No	ns	>0,9999
1 h	0	-1,498 to 1,498	No	ns	>0,9999
4 h	0	-1,320 to 1,320	No	ns	>0,9999
7 (days)	-0,5833	-1,908 to 0,7414	No	ns	0,6274
14 (days)	0,6	-3,679 to 4,879	No	ns	0,9887

Test details	Mean 1	Mean 2	Mean Diff,	SE of diff,	N1	N2	t	DF
Sham_S_M - Sham_S_F								
Pre-treatment	2	2	0	0,2582	4	6	0	5
1 h	2,5	2,5	0	0,4472	4	6	0	7,941
4 h	2,5	2,5	0	0,3651	4	6	0	6,316
7 (days)	2,25	2,833	-0,5833	0,3962	4	6	1,472	7,982
14 (days)	4	3,4	0,6	1,046	4	5	0,5738	4,813

Seizure latency: female vs male (within the CBD group)

Table Analyzed	Seizure latency females vs males CBD
Mixed-effects model (REML)	Matching: Stacked
Assume sphericity?	No
Alpha	0,05

Fixed effects (type III)	P value	P value summary	Statistically significant (P < 0,05)?	F (DFn, DFd)	Geisser-Greenhouse's epsilon
**Time**	**0,0041**	**	**Yes**	**F (1,858, 27,87) = 7,003**	0,4645
Sex	0,3847	ns	No	F (1, 60) = 0,7669	
Time x Sex	0,7277	ns	No	F (4, 60) = 0,5112	

Compare each cell mean with the other cell mean in that row	Multiple comparation
Number of families	1
Number of comparisons per family	5
**Alpha**	**0,05**

Sidak's multiple comparisons test	Mean Diff,	95,00% CI of diff,	Significant?	Summary	Adjusted P Value
CBD_S_M - CBD_S_F					
Pre-treatment	-0,25	-1,520 to 1,020	No	ns	0,9836
1 h	-0,3214	-1,981 to 1,338	No	ns	0,9855
4 h	-0,375	-3,316 to 2,566	No	ns	0,9978
7 (days)	0,55	-1,537 to 2,637	No	ns	0,9404
14 (days)	-1,333	-7,010 to 4,343	No	ns	0,9359

Test details	Mean 1	Mean 2	Mean Diff,	SE of diff,	N1	N2	t	DF
CBD_S_M - CBD_S_F								
Pre-treatment	1,875	2,125	-0,25	0,4173	8	8	0,5991	11,98
1 h	3,25	3,571	-0,3214	0,5529	8	7	0,5814	12,99
4 h	4,625	5	-0,375	0,9662	8	6	0,3881	11,99
7 (days)	4,75	4,2	0,55	0,6727	8	5	0,8176	10,86
14 (days)	3,5	4,833	-1,333	1,553	6	6	0,8587	6,142

B) Comparisons for wild-running duration:

Wild-running duration: Sham vs CBD (within the male group)

Table Analyzed	wild running phase Sham vs CBD males
Mixed-effects model (REML)	Matching: Stacked
Assume sphericity?	No
Alpha	0,05

Fixed effects (type III)	P value	P value summary	Statistically significant (P < 0,05)?	F (DFn, DFd)	Geisser-Greenhouse's epsilon
Time	0,1845	ns	No	F (1,987, 19,38) = 1,847	0,4969
Treatment	0,2075	ns	No	F (1, 10) = 1,816	
Time x Treatment	0,5042	ns	No	F (4, 39) = 0,8468	

Compare each cell mean with the other cell mean in that row	Multiple comparation
Number of families	1
Number of comparisons per family	5
Alpha	0,05

Sidak's multiple comparisons test	Mean Diff,	95,00% CI of diff,	Significant?	Summary	Adjusted P Value
Sham_S male - CBD_S male					
Pre-treatment	1,25	-4,817 to 7,317	No	ns	0,8672
1 h	-1,875	-3,174 to -0,5758	No	ns	
4 h	-0,75	-9,586 to 8,086	No	ns	0,9986
7 (days)	-3,875	-15,02 to 7,267	No	ns	0,8282
14 (days)	-6,036	-19,95 to 7,875	No	ns	0,6243

Test details	Mean 1	Mean 2	Mean Diff,	SE of diff,	N1	N2	t	DF
Sham_S male - CBD_S male								
Pre-treatment	5	3,75	1,25	1,092	4	8	1,144	3,139
1 h	3,75	5,625	-1,875	0,4092	4	8	4,583	9,75
4 h	5,5	6,25	-0,75	2,039	4	8	0,3678	4,355
7 (days)	5,75	9,625	-3,875	3,519	4	8	1,101	9,87
14 (days)	6,25	12,29	-6,036	4,047	4	7	1,491	7,296

Wild-running duration: Sham vs CBD (within the female group)

Table Analyzed	wild running phase Sham vs CBD females
Mixed-effects model (REML)	Matching: Stacked
Assume sphericity?	No
Alpha	0,05

Fixed effects (type III)	P value	P value summary	Statistically significant (P < 0,05)?	F (DFn, DFd)	Geisser-Greenhouse's epsilon
**Time**	**0,0444**	*	**Yes**	**F (2,869, 27,98) = 3,104**	0,7173
Treatment	0,0957	ns	No	F (1, 12) = 3,270	
**Time x Treatment**	**0,0327**	*	**Yes**	**F (4, 39) = 2,931**	

Compare each cell mean with the other cell mean in that row	Multiple comparation
Number of families	1
Number of comparisons per family	5
Alpha	0,05

Sidak's multiple comparisons test	Mean Diff,	95,00% CI of diff,	Significant?	Summary	Adjusted P Value
Sham_S female - CBD_S female					
Pre-treatment	2,333	-7,873 to 12,54	No	ns	0,9266
1 h	-10,29	-22,61 to 2,038	No	ns	0,1092
4 h	-4	-20,18 to 12,18	No	ns	0,9442
7 (days)	-6,7	-24,44 to 11,04	No	ns	0,6691
14 (days)	-4,5	-18,98 to 9,977	No	ns	0,8315

Test details	Mean 1	Mean 2	Mean Diff,	SE of diff,	N1	N2	t	DF
Sham_S female - CBD_S female								
Pre-treatment	6,333	4	2,333	2,586	6	8	0,9025	5,163
1 h	5	15,29	-10,29	3,562	6	7	2,887	7,152
4 h	8	12	-4	4,919	6	6	0,8131	8,458
7 (days)	7,5	14,2	-6,7	4,563	6	5	1,468	5,33
14 (days)	8,5	13	-4,5	3,977	6	5	1,132	6,208

Wild-running duration: female vs male (within the Sham group)

Table Analyzed	Wild running females vs males Sham
Mixed-effects model (REML)	Matching: Stacked
Assume sphericity?	No
Alpha	0,05

Fixed effects (type III)	P value	P value summary	Statistically significant (P < 0,05)?	F (DFn, DFd)	Geisser-Greenhouse's epsilon
Row Factor	0,2724	ns	No	F (2,226, 17,81) = 1,406	0,5566
Column Factor	0,4252	ns	No	F (1, 8) = 0,7059	
Row Factor x Column Factor	0,9885	ns	No	F (4, 32) = 0,07801	

Compare each cell mean with the other cell mean in that row	Multiple comparation
Number of families	1
Number of comparisons per family	5
Alpha	0,05

Sidak's multiple comparisons test	Mean Diff,	95,00% CI of diff,	Significant?	Summary	Adjusted P Value
Sham_S male - Sham_S female					
Pre-treatment	-1,333	-11,24 to 8,573	No	ns	0,9945
1 h	-1,25	-5,426 to 2,926	No	ns	0,8319
4 h	-2,5	-13,28 to 8,277	No	ns	0,9539
7 (days)	-1,75	-10,14 to 6,645	No	ns	0,9682
14 (days)	-2,25	-9,923 to 5,423	No	ns	0,8883

Test details	Mean 1	Mean 2	Mean Diff,	SE of diff,	N1	N2	t	DF
Sham_S male - Sham_S female								
Pre-treatment	5	6,333	-1,333	2,783	4	6	0,4791	6,586
1 h	3,75	5	-1,25	1,094	4	6	1,143	5,539
4 h	5,5	8	-2,5	3,217	4	6	0,7771	7,932
7 (days)	5,75	7,5	-1,75	2,459	4	6	0,7117	7,45
14 (days)	6,25	8,5	-2,25	2,29	4	6	0,9824	7,93

Wild-running duration: female vs male (within the CBD group)

Table Analyzed	wild running phase female vs males CBD
Mixed-effects model (REML)	Matching: Stacked
Assume sphericity?	No
Alpha	0,05

Fixed effects (type III)	P value	P value summary	Statistically significant (P < 0,05)?	F (DFn, DFd)	Geisser-Greenhouse's epsilon
**Time**	**0,0074**	**	**Yes**	**F (2,767, 31,82) = 4,944**	0,6918
Sex	0,0703	ns	No	F (1, 14) = 3,840	
Timer x Sex	0,1813	ns	No	F (4, 46) = 1,636	

Compare each cell mean with the other cell mean in that row	Multiple comparation
Number of families	1
Number of comparisons per family	5
Alpha	0,05

Sidak's multiple comparisons test	Mean Diff,	95,00% CI of diff,	Significant?	Summary	Adjusted P Value
CBD_S female - CBD_S male					
Pre-treatment	0,25	-0,8981 to 1,398	No	ns	0,9716
1 h	9,661	-2,850 to 22,17	No	ns	0,1387
4 h	5,75	-10,60 to 22,10	No	ns	0,7277
7 (days)	4,575	-12,83 to 21,98	No	ns	0,9258
14 (days)	0,7143	-15,73 to 17,15	No	ns	>0,9999

Test details	Mean 1	Mean 2	Mean Diff,	SE of diff,	N1	N2	t	DF
CBD_S female - CBD_S male								
Pre-treatment	4	3,75	0,25	0,366	8	8	0,6831	10,29
1 h	15,29	5,625	9,661	3,415	7	8	2,829	6,109
4 h	12	6,25	5,75	4,244	6	8	1,355	5,431
7 (days)	14,2	9,625	4,575	5,211	5	8	0,8779	8,02
14 (days)	13	12,29	0,7143	5,192	5	7	0,1376	9,869

C) Comparisons for convulsion phase duration:

Convulsive phase duration: Sham vs CBD (within the male group)

Table Analyzed	convulsive phase Sham vs CBD males
Mixed-effects model (REML)	Matching: Stacked
Assume sphericity?	No
Alpha	0,05

Fixed effects (type III)	P value	P value summary	Statistically significant (P < 0,05)?	F (DFn, DFd)	Geisser-Greenhouse's epsilon
**Time**	**0,0116**	*****	**Yes**	**F (2,439, 20,12) = 5,155**	0,6097
Treatment	0,699	ns	No	F (1, 10) = 0,1584	
Time x Treatment	0,8426	ns	No	F (4, 33) = 0,3494	

Compare each cell mean with the other cell mean in that row		Multiple comparation
Number of families	1	
Number of comparisons per family	5	
**Alpha**	**0,05**	

**Sidak's multiple comparisons test**	Mean Diff,	95,00% CI of diff,	Significant?	Summary	Adjusted P Value
Sham_S male - CBD_S male	-1,5	-12,94 to 9,944	No	ns	0,9895
Pre-treatment	0,375	-13,94 to 14,69	No	ns	>0,9999
1 h	0,3214	-6,808 to 7,450	No	ns	>0,9999
4 h	0,5833	-5,732 to 6,899	No	ns	0,9992
7 (days)	3,25	-8,705 to 15,20	No	ns	0,8204
14 (days)					

Test details	Mean 1	Mean 2	Mean Diff,	SE of diff,	N1	N2	t	DF
Sham_S male - CBD_S male								
Pre-treatment	29,75	31,25	-1,5	2,64	4	8	0,5682	4,353
1 h	26,25	25,88	0,375	3,799	4	8	0,0987	5,712
4 h	27,75	27,43	0,3214	1,788	4	7	0,1797	5,063
7 (days)	25,75	25,17	0,5833	1,832	4	6	0,3184	7,232
14 (days)	24	20,75	3,25	2,689	4	4	1,209	4,18

Convulsive phase duration: Sham vs CBD (within the female group)

Table Analyzed	convulsive phase Sham vs CBD females
Mixed-effects model (REML)	Matching: Stacked
Assume sphericity?	No
Alpha	0,05

Fixed effects (type III)	P value	P value summary	Statistically significant (P < 0,05)?	F (DFn, DFd)	Geisser-Greenhouse's epsilon
**Time**	**0,0013**	******	**Yes**	**F (2,406, 16,84) = 9,217**	0,6016
**Treatment**	**0,0395**	*****	**Yes**	**F (1, 12) = 5,334**	
Time x Treatment	0,3445	ns	No	F (4, 28) = 1,172	

Compare each cell mean with the other cell mean in that row		Multiple comparation
Number of families	1	
Number of comparisons per family	5	
**Alpha**	**0,05**	

**Sidak's multiple comparisons test**	Mean Diff,	95,00% CI of diff,	Significant?	Summary	Adjusted P Value
Sham_S female - CBD_S female					
Pre-treatment	0,5833	-8,315 to 9,482	No	ns	0,9999
1 h	1,933	-8,276 to 12,14	No	ns	0,9714
4 h	6,5	-4,000 to 17,00	No	ns	0,2979
7 (days)	7,833	-15,33 to 31,00	No	ns	0,579
14 (days)	1	-9,323 to 11,32	No	ns	0,9978

Test details	Mean 1	Mean 2	Mean Diff,	SE of diff,	N1	N2	t	DF
Sham_S female - CBD_S female								
Pre-treatment	33,33	32,75	0,5833	2,627	6	8	0,2221	7,64
1 h	27,6	25,67	1,933	2,752	5	3	0,7026	5,921
4 h	31,5	25	6,5	3,025	6	4	2,149	7,079
7 (days)	28,33	20,5	7,833	4,297	6	4	1,823	3,242
14 (days)	20,5	19,5	1	2,483	6	2	0,4027	4,677

Convulsive phase duration: female vs male (within the Sham group)

Table Analyzed	Convulsive phase females vs males Sham
Mixed-effects model (REML)	Matching: Stacked
Assume sphericity?	No
Alpha	0,05

Fixed effects (type III)	P value	P value summary	Statistically significant (P < 0,05)?	F (DFn, DFd)	Geisser-Greenhouse's epsilon
**Time**	**0,0195**	*****	**Yes**	**F (1,778, 13,78) = 5,548**	0,4446
Sex	0,4049	ns	No	F (1, 8) = 0,7732	
Time x Sex	0,4174	ns	No	F (4, 31) = 1,010	

Compare each cell mean with the other cell mean in that row		Multiple comparation
Number of families	1	
Number of comparisons per family	5	
**Alpha**	**0,05**	

**Sidak's multiple comparisons test**	Mean Diff,	95,00% CI of diff,	Significant?	Summary	Adjusted P Value
Sham_S male - Sham_S female					
Pre-treatment	-3,583	-15,02 to 7,853	No	ns	0,8518
1 h	-1,35	-16,04 to 13,34	No	ns	0,9989
4 h	-3,75	-14,38 to 6,879	No	ns	0,7864
7 (days)	-2,583	-6,680 to 1,514	No	ns	0,2892
14 (days)	3,5	-7,748 to 14,75	No	ns	0,8386

Test details	Mean 1	Mean 2	Mean Diff,	SE of diff,	N1	N2	t	DF
Sham_S male - Sham_S female								
Pre-treatment	29,75	33,33	-3,583	3,343	4	6	1,072	7,401
1 h	26,25	27,6	-1,35	3,94	4	5	0,3427	5,848
4 h	27,75	31,5	-3,75	3,116	4	6	1,203	7,475
7 (days)	25,75	28,33	-2,583	1,2	4	6	2,153	7,452
14 (days)	24	20,5	3,5	3,149	4	6	1,111	6,526

Convulsive phase duration: female vs male (within the CBD group)

Table Analyzed	convulsive phase males vs females CBD
Mixed-effects model (REML)	Matching: Stacked
Assume sphericity?	No
Alpha	0,05

Fixed effects (type III)	P value	P value summary	Statistically significant (P < 0,05)?	F (DFn, DFd)	Geisser-Greenhouse's epsilon
**Time**	**<0,0001**	****	**Yes**	**F (2,863, 21,47) = 12,01**	0,7158
Sex	0,2778	ns	No	F (1, 14) = 1,275	
Time x Sex	0,4713	ns	No	F (4, 30) = 0,9090	

Compare each cell mean with the other cell mean in that row		Multiple comparation
Number of families	1	
Number of comparisons per family	5	
**Alpha**	**0,05**	

**Sidak's multiple comparisons test**	Mean Diff,	95,00% CI of diff,	Significant?	Summary	Adjusted P Value
CBD_S female - CBD_S male					
Pre-treatment	1,5	-3,376 to 6,376	No	ns	0,9058
1 h	-0,2083	-8,581 to 8,164	No	ns	>0,9999
4 h	-2,429	-8,570 to 3,713	No	ns	0,6475
7 (days)	-4,667	-25,68 to 16,35	No	ns	0,8935
14 (days)	-1,25	-17,29 to 14,79	No	ns	0,9848

Test details	Mean 1	Mean 2	Mean Diff,	SE of diff,	N1	N2	t	DF
CBD_S female - CBD_S male								
Pre-treatment	32,75	31,25	1,5	1,642	8	8	0,9135	13,91
1 h	25,67	25,88	-0,2083	2,546	3	8	0,08182	8,476
4 h	25	27,43	-2,429	1,624	4	7	1,496	5,66
7 (days)	20,5	25,17	-4,667	4,514	4	6	1,034	3,903
14 (days)	19,5	20,75	-1,25	1,865	2	4	0,6702	2,175
