# Supplementary material for: Anticonvulsant effects, safety, and sex-dependent molecular signatures of cannabidiol treatment in a preclinical model of audiogenic epilepsy
Source: Front Pharmacol. 2026 Aug 7;17:1745696. doi: 10.3389/fphar.2026.1745696 (PMC13493383; doi:10.3389/fphar.2026.1745696)
Supplement: Supplementary file 4 [file DataSheet5.pdf]

**Supplementary Material 5. Full Statistical Summary of *Trpv1* gene expression in the inferior colliculus of GASH/Sal animals across chronic treatment, acoustic seizure stimulation and sex.**

| Three-way ANOVA | Ordinary | | | | |
|---|---|---|---|---|---|
| Alpha | 0.05 | | | | |
| | | | | | |
| Source of Variation | % of total variation | P value | P value summary | Significant? | |
| Sex | 0.01601 | 0.9236 | ns | No | |
| Treatment | 0.09056 | 0.8196 | ns | No | |
| Condition | 14.49 | 0.0062 | ** | Yes | |
| Sex x Treatment | 9.346 | 0.0253 | * | Yes | |
| Sex x Condition | 0.1326 | 0.7826 | ns | No | |
| Treatment x Condition | 2.321 | 0.2525 | ns | No | |
| Sex x Treatment x Condition | 13.89 | 0.0073 | ** | Yes | |
| | | | | | |
| ANOVA table | SS | DF | MS | F (DFn, DFd) | P value |
| Sex | 0.008047 | 1 | 0.008047 | F (1, 36) = 0.009333 | P=0.9236 |
| Treatment | 0.04552 | 1 | 0.04552 | F (1, 36) = 0.05279 | P=0.8196 |
| Condition | 7.283 | 1 | 7.283 | F (1, 36) = 8.447 | P=0.0062 |
| Sex x Treatment | 4.697 | 1 | 4.697 | F (1, 36) = 5.448 | P=0.0253 |
| Sex x Condition | 0.06663 | 1 | 0.06663 | F (1, 36) = 0.07727 | P=0.7826 |
| Treatment x Condition | 1.166 | 1 | 1.166 | F (1, 36) = 1.353 | P=0.2525 |
| Sex x Treatment x Condition | 6.982 | 1 | 6.982 | F (1, 36) = 8.097 | P=0.0073 |
| Residual | 31.04 | 36 | 0.8623 | | |
| | | | | | |
| Compare each cell mean with every other cell mean | | | | | |
| | | | | | |
| Number of families | 1 | | | | |
| Number of comparisons per family | 28 | | | | |
| Alpha | 0.05 | | | | |

| Tukey's multiple comparisons test | Mean Diff. | 95.00% CI of diff. | Significant? | Summary | Adjusted P Value |
|---|---|---|---|---|---|
| Female:Sham_U vs. Female:CBD_U | 1.223 | -0.5853 to 3.031 | No | ns | 0.3901 |
| Female:Sham_U vs. Female:Sham_S | 1.204 | -0.5194 to 2.928 | No | ns | 0.3497 |
| Female:Sham_U vs. Female:CBD_S | 1.473 | -0.2512 to 3.197 | No | ns | 0.1414 |
| Female:Sham_U vs. Male:Sham_U | 1.363 | -0.4451 to 3.171 | No | ns | 0.261 |
| Female:Sham_U vs. Male:CBD_U | 1.129 | -0.6789 to 2.937 | No | ns | 0.4908 |
| Female:Sham_U vs. Male:Sham_S | -0.371 | -2.298 to 1.556 | No | ns | 0.9984 |
| Female:Sham_U vs. Male:CBD_S | 1.669 | 0.008061 to 3.330 | Yes | * | 0.0482 |
| Female:CBD_U vs. Female:Sham_S | -0.0183 | -1.826 to 1.790 | No | ns | >0.9999 |
| Female:CBD_U vs. Female:CBD_S | 0.25 | -1.558 to 2.058 | No | ns | 0.9998 |
| Female:CBD_U vs. Male:Sham_U | 0.1402 | -1.748 to 2.029 | No | ns | >0.9999 |
| Female:CBD_U vs. Male:CBD_U | -0.09363 | -1.982 to 1.795 | No | ns | >0.9999 |
| Female:CBD_U vs. Male:Sham_S | -1.594 | -3.597 to 0.4093 | No | ns | 0.2046 |
| Female:CBD_U vs. Male:CBD_S | 0.4465 | -1.302 to 2.195 | No | ns | 0.9907 |
| Female:Sham_S vs. Female:CBD_S | 0.2683 | -1.456 to 1.992 | No | ns | 0.9996 |
| Female:Sham_S vs. Male:Sham_U | 0.1585 | -1.650 to 1.966 | No | ns | >0.9999 |
| Female:Sham_S vs. Male:CBD_U | -0.07533 | -1.883 to 1.733 | No | ns | >0.9999 |
| Female:Sham_S vs. Male:Sham_S | -1.575 | -3.503 to 0.3520 | No | ns | 0.1791 |
| Female:Sham_S vs. Male:CBD_S | 0.4648 | -1.196 to 2.126 | No | ns | 0.9842 |
| Female:CBD_S vs. Male:Sham_U | -0.1098 | -1.918 to 1.698 | No | ns | >0.9999 |
| Female:CBD_S vs. Male:CBD_U | -0.3436 | -2.152 to 1.464 | No | ns | 0.9985 |
| Female:CBD_S vs. Male:Sham_S | -1.844 | -3.771 to 0.08367 | No | ns | 0.0693 |
| Female:CBD_S vs. Male:CBD_S | 0.1965 | -1.465 to 1.858 | No | ns | >0.9999 |
| Male:Sham_U vs. Male:CBD_U | -0.2338 | -2.122 to 1.655 | No | ns | >0.9999 |
| Male:Sham_U vs. Male:Sham_S | -1.734 | -3.737 to 0.2691 | No | ns | 0.1312 |
| Male:Sham_U vs. Male:CBD_S | 0.3063 | -1.442 to 2.055 | No | ns | 0.9991 |
| Male:CBD_U vs. Male:Sham_S | -1.5 | -3.503 to 0.5029 | No | ns | 0.2682 |
| Male:CBD_U vs. Male:CBD_S | 0.5401 | -1.208 to 2.288 | No | ns | 0.9725 |
| Male:Sham_S vs. Male:CBD_S | 2.04 | 0.1687 to 3.912 | Yes | * | 0.0245 |

| Test details | Mean 1 | Mean 2 | Mean Diff. | SE of diff. | N1 | N2 | q | DF |
|---|---|---|---|---|---|---|---|---|
| Female:Sham_U vs. Female:CBD_U | 11.3 | 10.08 | 1.223 | 0.5623 | 6 | 5 | 3.075 | 36 |
| Female:Sham_U vs. Female:Sham_S | 11.3 | 10.1 | 1.204 | 0.5361 | 6 | 6 | 3.177 | 36 |
| Female:Sham_U vs. Female:CBD_S | 11.3 | 9.827 | 1.473 | 0.5361 | 6 | 6 | 3.885 | 36 |
| Female:Sham_U vs. Male:Sham_U | 11.3 | 9.937 | 1.363 | 0.5623 | 6 | 5 | 3.428 | 36 |
| Female:Sham_U vs. Male:CBD_U | 11.3 | 10.17 | 1.129 | 0.5623 | 6 | 5 | 2.84 | 36 |
| Female:Sham_U vs. Male:Sham_S | 11.3 | 11.67 | -0.371 | 0.5994 | 6 | 4 | 0.8753 | 36 |
| Female:Sham_U vs. Male:CBD_S | 11.3 | 9.631 | 1.669 | 0.5166 | 6 | 7 | 4.569 | 36 |
| Female:CBD_U vs. Female:Sham_S | 10.08 | 10.1 | -0.0183 | 0.5623 | 5 | 6 | 0.04603 | 36 |
| Female:CBD_U vs. Female:CBD_S | 10.08 | 9.827 | 0.25 | 0.5623 | 5 | 6 | 0.6287 | 36 |
| Female:CBD_U vs. Male:Sham_U | 10.08 | 9.937 | 0.1402 | 0.5873 | 5 | 5 | 0.3375 | 36 |
| Female:CBD_U vs. Male:CBD_U | 10.08 | 10.17 | -0.09363 | 0.5873 | 5 | 5 | 0.2255 | 36 |
| Female:CBD_U vs. Male:Sham_S | 10.08 | 11.67 | -1.594 | 0.6229 | 5 | 4 | 3.618 | 36 |
| Female:CBD_U vs. Male:CBD_S | 10.08 | 9.631 | 0.4465 | 0.5437 | 5 | 7 | 1.161 | 36 |
| Female:Sham_S vs. Female:CBD_S | 10.1 | 9.827 | 0.2683 | 0.5361 | 6 | 6 | 0.7077 | 36 |
| Female:Sham_S vs. Male:Sham_U | 10.1 | 9.937 | 0.1585 | 0.5623 | 6 | 5 | 0.3986 | 36 |
| Female:Sham_S vs. Male:CBD_U | 10.1 | 10.17 | -0.07533 | 0.5623 | 6 | 5 | 0.1895 | 36 |
| Female:Sham_S vs. Male:Sham_S | 10.1 | 11.67 | -1.575 | 0.5994 | 6 | 4 | 3.717 | 36 |
| Female:Sham_S vs. Male:CBD_S | 10.1 | 9.631 | 0.4648 | 0.5166 | 6 | 7 | 1.272 | 36 |
| Female:CBD_S vs. Male:Sham_U | 9.827 | 9.937 | -0.1098 | 0.5623 | 6 | 5 | 0.2762 | 36 |
| Female:CBD_S vs. Male:CBD_U | 9.827 | 10.17 | -0.3436 | 0.5623 | 6 | 5 | 0.8642 | 36 |
| Female:CBD_S vs. Male:Sham_S | 9.827 | 11.67 | -1.844 | 0.5994 | 6 | 4 | 4.35 | 36 |
| Female:CBD_S vs. Male:CBD_S | 9.827 | 9.631 | 0.1965 | 0.5166 | 6 | 7 | 0.538 | 36 |
| Male:Sham_U vs. Male:CBD_U | 9.937 | 10.17 | -0.2338 | 0.5873 | 5 | 5 | 0.563 | 36 |
| Male:Sham_U vs. Male:Sham_S | 9.937 | 11.67 | -1.734 | 0.6229 | 5 | 4 | 3.936 | 36 |
| Male:Sham_U vs. Male:CBD_S | 9.937 | 9.631 | 0.3063 | 0.5437 | 5 | 7 | 0.7968 | 36 |
| Male:CBD_U vs. Male:Sham_S | 10.17 | 11.67 | -1.5 | 0.6229 | 5 | 4 | 3.406 | 36 |
| Male:CBD_U vs. Male:CBD_S | 10.17 | 9.631 | 0.5401 | 0.5437 | 5 | 7 | 1.405 | 36 |
| Male:Sham_S vs. Male:CBD_S | 11.67 | 9.631 | 2.04 | 0.582 | 4 | 7 | 4.957 | 36 |

**Supplementary Material 5. Full Statistical Summary of *5-Htr1a* gene expression in the inferior colliculus of GASH/Sal animals across chronic treatment, acoustic seizure stimulation and sex.**

| Three-way ANOVA | Ordinary | | | | |
|---|---|---|---|---|---|
| Alpha | 0.05 | | | | |
| | | | | | |
| Source of Variation | % of total variation | P value | P value summary | Significant? | |
| Sex | 44.31 | <0.0001 | **** | Yes | |
| Treatment | 6.878 | 0.0092 | ** | Yes | |
| Condition | 0.0000958 | 0.9919 | ns | No | |
| Sex x Treatment | 9.162 | 0.003 | ** | Yes | |
| Sex x Condition | 1.759 | 0.1724 | ns | No | |
| Treatment x Condition | 4.199 | 0.0382 | * | Yes | |
| Sex x Treatment x Condition | 2.399 | 0.1127 | ns | No | |
| | | | | | |
| ANOVA table | SS | DF | MS | F (DFn, DFd) | P value |
| Sex | 76.83 | 1 | 76.83 | F (1, 36) = 48.84 | P<0.0001 |
| Treatment | 11.93 | 1 | 11.93 | F (1, 36) = 7.581 | P=0.0092 |
| Condition | 0.0001661 | 1 | 0.0001661 | F (1, 36) = 0.000105 | P=0.9919 |
| Sex x Treatment | 15.89 | 1 | 15.89 | F (1, 36) = 10.10 | P=0.0030 |
| Sex x Condition | 3.05 | 1 | 3.05 | F (1, 36) = 1.939 | P=0.1724 |
| Treatment x Condition | 7.28 | 1 | 7.28 | F (1, 36) = 4.628 | P=0.0382 |
| Sex x Treatment x Condition | 4.159 | 1 | 4.159 | F (1, 36) = 2.644 | P=0.1127 |
| Residual | 56.63 | 36 | 1.573 | | |
| | | | | | |
| Compare each cell mean with every other cell mean | | | | | |
| | | | | | |
| Number of families | 1 | | | | |
| Number of comparisons per family | 28 | | | | |
| Alpha | 0.05 | | | | |

| Tukey's multiple comparisons test | Mean Diff. | 95.00% CI of diff. | Significant? | Summary | Adjusted P Value |
|---|---|---|---|---|---|
| Female:Sham_U vs. Female:CBD_U | -0.9098 | -3.352 to 1.532 | No | ns | 0.9273 |
| Female:Sham_U vs. Female:Sham_S | -1.285 | -3.613 to 1.044 | No | ns | 0.6408 |
| Female:Sham_U vs. Female:CBD_S | 0.7 | -1.628 to 3.028 | No | ns | 0.9763 |
| Female:Sham_U vs. Male:Sham_U | 3.805 | 1.363 to 6.247 | Yes | *** | 0.0004 |
| Female:Sham_U vs. Male:CBD_U | 3.075 | 0.6327 to 5.517 | Yes | ** | 0.0058 |
| Female:Sham_U vs. Male:Sham_S | 1.332 | -1.271 to 3.935 | No | ns | 0.7206 |
| Female:Sham_U vs. Male:CBD_S | 1.004 | -1.240 to 3.247 | No | ns | 0.8334 |
| Female:CBD_U vs. Female:Sham_S | -0.3747 | -2.817 to 2.067 | No | ns | 0.9996 |
| Female:CBD_U vs. Female:CBD_S | 1.61 | -0.8323 to 4.052 | No | ns | 0.4223 |
| Female:CBD_U vs. Male:Sham_U | 4.715 | 2.165 to 7.266 | Yes | **** | <0.0001 |
| Female:CBD_U vs. Male:CBD_U | 3.984 | 1.434 to 6.535 | Yes | *** | 0.0003 |
| Female:CBD_U vs. Male:Sham_S | 2.242 | -0.4637 to 4.947 | No | ns | 0.1669 |
| Female:CBD_U vs. Male:CBD_S | 1.913 | -0.4481 to 4.275 | No | ns | 0.1872 |
| Female:Sham_S vs. Female:CBD_S | 1.985 | -0.3439 to 4.313 | No | ns | 0.1433 |
| Female:Sham_S vs. Male:Sham_U | 5.09 | 2.648 to 7.532 | Yes | **** | <0.0001 |
| Female:Sham_S vs. Male:CBD_U | 4.359 | 1.917 to 6.801 | Yes | **** | <0.0001 |
| Female:Sham_S vs. Male:Sham_S | 2.616 | 0.01312 to 5.220 | Yes | * | 0.0481 |
| Female:Sham_S vs. Male:CBD_S | 2.288 | 0.04438 to 4.532 | Yes | * | 0.0429 |
| Female:CBD_S vs. Male:Sham_U | 3.105 | 0.6634 to 5.547 | Yes | ** | 0.0051 |
| Female:CBD_S vs. Male:CBD_U | 2.375 | -0.06733 to 4.817 | No | ns | 0.0616 |
| Female:CBD_S vs. Male:Sham_S | 0.6318 | -1.971 to 3.235 | No | ns | 0.9931 |
| Female:CBD_S vs. Male:CBD_S | 0.3036 | -1.940 to 2.547 | No | ns | 0.9998 |
| Male:Sham_U vs. Male:CBD_U | -0.7307 | -3.281 to 1.820 | No | ns | 0.9819 |
| Male:Sham_U vs. Male:Sham_S | -2.474 | -5.179 to 0.2318 | No | ns | 0.094 |
| Male:Sham_U vs. Male:CBD_S | -2.802 | -5.163 to -0.4404 | Yes | * | 0.0109 |
| Male:CBD_U vs. Male:Sham_S | -1.743 | -4.448 to 0.9625 | No | ns | 0.4514 |
| Male:CBD_U vs. Male:CBD_S | -2.071 | -4.433 to 0.2903 | No | ns | 0.1215 |
| Male:Sham_S vs. Male:CBD_S | -0.3283 | -2.856 to 2.199 | No | ns | 0.9999 |

| Test details | Mean 1 | Mean 2 | Mean Diff. | SE of diff. | N1 | N2 | q | DF |
|---|---|---|---|---|---|---|---|---|
| Female:Sham_U vs. Female:CBD_U | 9.32 | 10.23 | -0.9098 | 0.7595 | 6 | 5 | 1.694 | 36 |
| Female:Sham_U vs. Female:Sham_S | 9.32 | 10.6 | -1.285 | 0.7241 | 6 | 6 | 2.509 | 36 |
| Female:Sham_U vs. Female:CBD_S | 9.32 | 8.62 | 0.7 | 0.7241 | 6 | 6 | 1.367 | 36 |
| Female:Sham_U vs. Male:Sham_U | 9.32 | 5.514 | 3.805 | 0.7595 | 6 | 5 | 7.086 | 36 |
| Female:Sham_U vs. Male:CBD_U | 9.32 | 6.245 | 3.075 | 0.7595 | 6 | 5 | 5.725 | 36 |
| Female:Sham_U vs. Male:Sham_S | 9.32 | 7.988 | 1.332 | 0.8096 | 6 | 4 | 2.326 | 36 |
| Female:Sham_U vs. Male:CBD_S | 9.32 | 8.316 | 1.004 | 0.6978 | 6 | 7 | 2.034 | 36 |
| Female:CBD_U vs. Female:Sham_S | 10.23 | 10.6 | -0.3747 | 0.7595 | 5 | 6 | 0.6978 | 36 |
| Female:CBD_U vs. Female:CBD_S | 10.23 | 8.62 | 1.61 | 0.7595 | 5 | 6 | 2.998 | 36 |
| Female:CBD_U vs. Male:Sham_U | 10.23 | 5.514 | 4.715 | 0.7932 | 5 | 5 | 8.406 | 36 |
| Female:CBD_U vs. Male:CBD_U | 10.23 | 6.245 | 3.984 | 0.7932 | 5 | 5 | 7.104 | 36 |
| Female:CBD_U vs. Male:Sham_S | 10.23 | 7.988 | 2.242 | 0.8414 | 5 | 4 | 3.768 | 36 |
| Female:CBD_U vs. Male:CBD_S | 10.23 | 8.316 | 1.913 | 0.7344 | 5 | 7 | 3.684 | 36 |
| Female:Sham_S vs. Female:CBD_S | 10.6 | 8.62 | 1.985 | 0.7241 | 6 | 6 | 3.876 | 36 |
| Female:Sham_S vs. Male:Sham_U | 10.6 | 5.514 | 5.09 | 0.7595 | 6 | 5 | 9.478 | 36 |
| Female:Sham_S vs. Male:CBD_U | 10.6 | 6.245 | 4.359 | 0.7595 | 6 | 5 | 8.117 | 36 |
| Female:Sham_S vs. Male:Sham_S | 10.6 | 7.988 | 2.616 | 0.8096 | 6 | 4 | 4.57 | 36 |
| Female:Sham_S vs. Male:CBD_S | 10.6 | 8.316 | 2.288 | 0.6978 | 6 | 7 | 4.637 | 36 |
| Female:CBD_S vs. Male:Sham_U | 8.62 | 5.514 | 3.105 | 0.7595 | 6 | 5 | 5.783 | 36 |
| Female:CBD_S vs. Male:CBD_U | 8.62 | 6.245 | 2.375 | 0.7595 | 6 | 5 | 4.422 | 36 |
| Female:CBD_S vs. Male:Sham_S | 8.62 | 7.988 | 0.6318 | 0.8096 | 6 | 4 | 1.104 | 36 |
| Female:CBD_S vs. Male:CBD_S | 8.62 | 8.316 | 0.3036 | 0.6978 | 6 | 7 | 0.6153 | 36 |
| Male:Sham_U vs. Male:CBD_U | 5.514 | 6.245 | -0.7307 | 0.7932 | 5 | 5 | 1.303 | 36 |
| Male:Sham_U vs. Male:Sham_S | 5.514 | 7.988 | -2.474 | 0.8414 | 5 | 4 | 4.158 | 36 |
| Male:Sham_U vs. Male:CBD_S | 5.514 | 8.316 | -2.802 | 0.7344 | 5 | 7 | 5.395 | 36 |
| Male:CBD_U vs. Male:Sham_S | 6.245 | 7.988 | -1.743 | 0.8414 | 5 | 4 | 2.93 | 36 |
| Male:CBD_U vs. Male:CBD_S | 6.245 | 8.316 | -2.071 | 0.7344 | 5 | 7 | 3.988 | 36 |
| Male:Sham_S vs. Male:CBD_S | 7.988 | 8.316 | -0.3283 | 0.7861 | 4 | 7 | 0.5905 | 36 |

**Supplementary Material 5. Full Statistical Summary of *Sigmar1* gene expression in the inferior colliculus of GASH/Sal animals across chronic treatment, acoustic seizure stimulation and sex.**

| Three-way ANOVA | Ordinary | | | | |
|---|---|---|---|---|---|
| Alpha | 0.05 | | | | |
| | | | | | |
| Source of Variation | % of total variation | P value | P value summary | Significant? | |
| Sex | 19.89 | 0.0011 | ** | Yes | |
| Treatment | 6.124 | 0.0569 | ns | No | |
| Condition | 1.565 | 0.3265 | ns | No | |
| Sex x Treatment | 0.5521 | 0.5583 | ns | No | |
| Sex x Condition | 7.203 | 0.0397 | * | Yes | |
| Treatment x Condition | 2.96 | 0.1798 | ns | No | |
| Sex x Treatment x Condition | 3.614 | 0.1394 | ns | No | |
| | | | | | |
| ANOVA table | SS | DF | MS | F (DFn, DFd) | P value |
| Sex | 7.219 | 1 | 7.219 | F (1, 36) = 12.57 | P=0.0011 |
| Treatment | 2.223 | 1 | 2.223 | F (1, 36) = 3.871 | P=0.0569 |
| Condition | 0.5681 | 1 | 0.5681 | F (1, 36) = 0.9895 | P=0.3265 |
| Sex x Treatment | 0.2004 | 1 | 0.2004 | F (1, 36) = 0.3490 | P=0.5583 |
| Sex x Condition | 2.614 | 1 | 2.614 | F (1, 36) = 4.554 | P=0.0397 |
| Treatment x Condition | 1.074 | 1 | 1.074 | F (1, 36) = 1.871 | P=0.1798 |
| Sex x Treatment x Condition | 1.312 | 1 | 1.312 | F (1, 36) = 2.285 | P=0.1394 |
| Residual | 20.67 | 36 | 0.5741 | | |
| | | | | | |
| Number of comparisons per family | 28 | | | | |
| Alpha | 0.05 | | | | |

| Tukey's multiple comparisons test | Mean Diff. | 95.00% CI of diff. | Significant? | Summary | Adjusted P Value |
|---|---|---|---|---|---|
| Female:Sham_U vs. Female:CBD_U | 1.391 | -0.08472 to 2.866 | No | ns | 0.0768 |
| Female:Sham_U vs. Female:Sham_S | 0.07433 | -1.332 to 1.481 | No | ns | >0.9999 |
| Female:Sham_U vs. Female:CBD_S | 0.132 | -1.275 to 1.539 | No | ns | >0.9999 |
| Female:Sham_U vs. Male:Sham_U | 1.528 | 0.05238 to 3.003 | Yes | * | 0.0379 |
| Female:Sham_U vs. Male:CBD_U | 1.231 | -0.2445 to 2.706 | No | ns | 0.161 |
| Female:Sham_U vs. Male:Sham_S | 1.176 | -0.3969 to 2.748 | No | ns | 0.2701 |
| Female:Sham_U vs. Male:CBD_S | 0.9454 | -0.4101 to 2.301 | No | ns | 0.3519 |
| Female:CBD_U vs. Female:Sham_S | -1.316 | -2.792 to 0.1591 | No | ns | 0.1096 |
| Female:CBD_U vs. Female:CBD_S | -1.259 | -2.734 to 0.2167 | No | ns | 0.1426 |
| Female:CBD_U vs. Male:Sham_U | 0.1371 | -1.404 to 1.678 | No | ns | >0.9999 |
| Female:CBD_U vs. Male:CBD_U | -0.1598 | -1.701 to 1.381 | No | ns | >0.9999 |
| Female:CBD_U vs. Male:Sham_S | -0.2148 | -1.849 to 1.420 | No | ns | 0.9999 |
| Female:CBD_U vs. Male:CBD_S | -0.4452 | -1.872 to 0.9814 | No | ns | 0.9709 |
| Female:Sham_S vs. Female:CBD_S | 0.05767 | -1.349 to 1.464 | No | ns | >0.9999 |
| Female:Sham_S vs. Male:Sham_U | 1.453 | -0.02195 to 2.929 | No | ns | 0.056 |
| Female:Sham_S vs. Male:CBD_U | 1.156 | -0.3189 to 2.632 | No | ns | 0.2195 |
| Female:Sham_S vs. Male:Sham_S | 1.101 | -0.4712 to 2.674 | No | ns | 0.3468 |
| Female:Sham_S vs. Male:CBD_S | 0.871 | -0.4845 to 2.227 | No | ns | 0.4546 |
| Female:CBD_S vs. Male:Sham_U | 1.396 | -0.07962 to 2.871 | No | ns | 0.0749 |
| Female:CBD_S vs. Male:CBD_U | 1.099 | -0.3765 to 2.574 | No | ns | 0.2744 |
| Female:CBD_S vs. Male:Sham_S | 1.044 | -0.5289 to 2.616 | No | ns | 0.4137 |
| Female:CBD_S vs. Male:CBD_S | 0.8134 | -0.5421 to 2.169 | No | ns | 0.5408 |
| Male:Sham_U vs. Male:CBD_U | -0.2969 | -1.838 to 1.244 | No | ns | 0.9984 |
| Male:Sham_U vs. Male:Sham_S | -0.3519 | -1.986 to 1.282 | No | ns | 0.9967 |
| Male:Sham_U vs. Male:CBD_S | -0.5823 | -2.009 to 0.8443 | No | ns | 0.8881 |
| Male:CBD_U vs. Male:Sham_S | -0.05498 | -1.689 to 1.579 | No | ns | >0.9999 |
| Male:CBD_U vs. Male:CBD_S | -0.2854 | -1.712 to 1.141 | No | ns | 0.9979 |
| Male:Sham_S vs. Male:CBD_S | -0.2304 | -1.758 to 1.297 | No | ns | 0.9997 |

| Test details | Mean 1 | Mean 2 | Mean Diff. | SE of diff. | N1 | N2 | q | DF |
|---|---|---|---|---|---|---|---|---|
| Female:Sham_U vs. Female:CBD_U | 4.205 | 2.814 | 1.391 | 0.4588 | 6 | 5 | 4.286 | 36 |
| Female:Sham_U vs. Female:Sham_S | 4.205 | 4.131 | 0.07433 | 0.4375 | 6 | 6 | 0.2403 | 36 |
| Female:Sham_U vs. Female:CBD_S | 4.205 | 4.073 | 0.132 | 0.4375 | 6 | 6 | 0.4267 | 36 |
| Female:Sham_U vs. Male:Sham_U | 4.205 | 2.677 | 1.528 | 0.4588 | 6 | 5 | 4.709 | 36 |
| Female:Sham_U vs. Male:CBD_U | 4.205 | 2.974 | 1.231 | 0.4588 | 6 | 5 | 3.794 | 36 |
| Female:Sham_U vs. Male:Sham_S | 4.205 | 3.029 | 1.176 | 0.4891 | 6 | 4 | 3.4 | 36 |
| Female:Sham_U vs. Male:CBD_S | 4.205 | 3.26 | 0.9454 | 0.4216 | 6 | 7 | 3.171 | 36 |
| Female:CBD_U vs. Female:Sham_S | 2.814 | 4.131 | -1.316 | 0.4588 | 5 | 6 | 4.057 | 36 |
| Female:CBD_U vs. Female:CBD_S | 2.814 | 4.073 | -1.259 | 0.4588 | 5 | 6 | 3.879 | 36 |
| Female:CBD_U vs. Male:Sham_U | 2.814 | 2.677 | 0.1371 | 0.4792 | 5 | 5 | 0.4046 | 36 |
| Female:CBD_U vs. Male:CBD_U | 2.814 | 2.974 | -0.1598 | 0.4792 | 5 | 5 | 0.4716 | 36 |
| Female:CBD_U vs. Male:Sham_S | 2.814 | 3.029 | -0.2148 | 0.5083 | 5 | 4 | 0.5976 | 36 |
| Female:CBD_U vs. Male:CBD_S | 2.814 | 3.26 | -0.4452 | 0.4437 | 5 | 7 | 1.419 | 36 |
| Female:Sham_S vs. Female:CBD_S | 4.131 | 4.073 | 0.05767 | 0.4375 | 6 | 6 | 0.1864 | 36 |
| Female:Sham_S vs. Male:Sham_U | 4.131 | 2.677 | 1.453 | 0.4588 | 6 | 5 | 4.48 | 36 |
| Female:Sham_S vs. Male:CBD_U | 4.131 | 2.974 | 1.156 | 0.4588 | 6 | 5 | 3.564 | 36 |
| Female:Sham_S vs. Male:Sham_S | 4.131 | 3.029 | 1.101 | 0.4891 | 6 | 4 | 3.185 | 36 |
| Female:Sham_S vs. Male:CBD_S | 4.131 | 3.26 | 0.871 | 0.4216 | 6 | 7 | 2.922 | 36 |
| Female:CBD_S vs. Male:Sham_U | 4.073 | 2.677 | 1.396 | 0.4588 | 6 | 5 | 4.302 | 36 |
| Female:CBD_S vs. Male:CBD_U | 4.073 | 2.974 | 1.099 | 0.4588 | 6 | 5 | 3.387 | 36 |
| Female:CBD_S vs. Male:Sham_S | 4.073 | 3.029 | 1.044 | 0.4891 | 6 | 4 | 3.018 | 36 |
| Female:CBD_S vs. Male:CBD_S | 4.073 | 3.26 | 0.8134 | 0.4216 | 6 | 7 | 2.729 | 36 |
| Male:Sham_U vs. Male:CBD_U | 2.677 | 2.974 | -0.2969 | 0.4792 | 5 | 5 | 0.8762 | 36 |
| Male:Sham_U vs. Male:Sham_S | 2.677 | 3.029 | -0.3519 | 0.5083 | 5 | 4 | 0.9791 | 36 |
| Male:Sham_U vs. Male:CBD_S | 2.677 | 3.26 | -0.5823 | 0.4437 | 5 | 7 | 1.856 | 36 |
| Male:CBD_U vs. Male:Sham_S | 2.974 | 3.029 | -0.05498 | 0.5083 | 5 | 4 | 0.153 | 36 |
| Male:CBD_U vs. Male:CBD_S | 2.974 | 3.26 | -0.2854 | 0.4437 | 5 | 7 | 0.9097 | 36 |
| Male:Sham_S vs. Male:CBD_S | 3.029 | 3.26 | -0.2304 | 0.4749 | 4 | 7 | 0.6862 | 36 |

**Supplementary Material 5. Full Statistical Summary of *Adora1* gene expression in the inferior colliculus of GASH/Sal animals across chronic treatment, acoustic seizure stimulation and sex.**

| Three-way ANOVA | Ordinary | | | | |
|---|---|---|---|---|---|
| Alpha | 0.05 | | | | |
| | | | | | |
| Source of Variation | % of total variation | P value | P value summary | Significant? | |
| Sex | 38.34 | <0.0001 | **** | Yes | |
| Treatment | 0.9088 | 0.3316 | ns | No | |
| Condition | 0.1389 | 0.7027 | ns | No | |
| Sex x Treatment | 23.1 | <0.0001 | **** | Yes | |
| Sex x Condition | 1.746 | 0.181 | ns | No | |
| Treatment x Condition | 1.756 | 0.1798 | ns | No | |
| Sex x Treatment x Condition | 0.3699 | 0.534 | ns | No | |
| | | | | | |
| ANOVA table | SS | DF | MS | F (DFn, DFd) | P value |
| Sex | 31.08 | 1 | 31.08 | F (1, 36) = 40.86 | P<0.0001 |
| Treatment | 0.7367 | 1 | 0.7367 | F (1, 36) = 0.9686 | P=0.3316 |
| Condition | 0.1126 | 1 | 0.1126 | F (1, 36) = 0.1481 | P=0.7027 |
| Sex x Treatment | 18.73 | 1 | 18.73 | F (1, 36) = 24.62 | P<0.0001 |
| Sex x Condition | 1.415 | 1 | 1.415 | F (1, 36) = 1.860 | P=0.1810 |
| Treatment x Condition | 1.424 | 1 | 1.424 | F (1, 36) = 1.872 | P=0.1798 |
| Sex x Treatment x Condition | 0.2999 | 1 | 0.2999 | F (1, 36) = 0.3943 | P=0.5340 |
| Residual | 27.38 | 36 | 0.7606 | | |
| | | | | | |
| Compare each cell mean with every other cell mean | | | | | |
| | | | | | |
| Number of families | 1 | | | | |
| Number of comparisons per family | 28 | | | | |
| Alpha | 0.05 | | | | |

| Tukey's multiple comparisons test | Mean Diff. | 95.00% CI of diff. | Significant? | Summary | Adjusted P Value |
|---|---|---|---|---|---|
| Female:Sham_U vs. Female:CBD_U | 0.9976 | -0.7005 to 2.696 | No | ns | 0.5668 |
| Female:Sham_U vs. Female:Sham_S | 1.591 | -0.02771 to 3.210 | No | ns | 0.0569 |
| Female:Sham_U vs. Female:CBD_S | 1.525 | -0.09358 to 3.145 | No | ns | 0.077 |
| Female:Sham_U vs. Male:Sham_U | 3.555 | 1.857 to 5.253 | Yes | **** | <0.0001 |
| Female:Sham_U vs. Male:CBD_U | 3.492 | 1.794 to 5.190 | Yes | **** | <0.0001 |
| Female:Sham_U vs. Male:Sham_S | 2.169 | 0.3584 to 3.979 | Yes | ** | 0.0098 |
| Female:Sham_U vs. Male:CBD_S | 1.711 | 0.1503 to 3.271 | Yes | * | 0.0233 |
| Female:CBD_U vs. Female:Sham_S | 0.5937 | -1.104 to 2.292 | No | ns | 0.947 |
| Female:CBD_U vs. Female:CBD_S | 0.5279 | -1.170 to 2.226 | No | ns | 0.9715 |
| Female:CBD_U vs. Male:Sham_U | 2.558 | 0.7841 to 4.331 | Yes | ** | 0.0011 |
| Female:CBD_U vs. Male:CBD_U | 2.494 | 0.7205 to 4.268 | Yes | ** | 0.0015 |
| Female:CBD_U vs. Male:Sham_S | 1.171 | -0.7102 to 3.052 | No | ns | 0.4949 |
| Female:CBD_U vs. Male:CBD_S | 0.7129 | -0.9291 to 2.355 | No | ns | 0.8531 |
| Female:Sham_S vs. Female:CBD_S | -0.06587 | -1.685 to 1.553 | No | ns | >0.9999 |
| Female:Sham_S vs. Male:Sham_U | 1.964 | 0.2659 to 3.662 | Yes | * | 0.0141 |
| Female:Sham_S vs. Male:CBD_U | 1.9 | 0.2022 to 3.598 | Yes | * | 0.0193 |
| Female:Sham_S vs. Male:Sham_S | 0.5772 | -1.233 to 2.387 | No | ns | 0.9673 |
| Female:Sham_S vs. Male:CBD_S | 0.1192 | -1.441 to 1.679 | No | ns | >0.9999 |
| Female:CBD_S vs. Male:Sham_U | 2.03 | 0.3318 to 3.728 | Yes | * | 0.0101 |
| Female:CBD_S vs. Male:CBD_U | 1.966 | 0.2681 to 3.664 | Yes | * | 0.0139 |
| Female:CBD_S vs. Male:Sham_S | 0.6431 | -1.167 to 2.453 | No | ns | 0.9426 |
| Female:CBD_S vs. Male:CBD_S | 0.185 | -1.375 to 1.745 | No | ns | >0.9999 |
| Male:Sham_U vs. Male:CBD_U | -0.06366 | -1.837 to 1.710 | No | ns | >0.9999 |
| Male:Sham_U vs. Male:Sham_S | -1.387 | -3.268 to 0.4944 | No | ns | 0.2861 |
| Male:Sham_U vs. Male:CBD_S | -1.845 | -3.487 to -0.2028 | Yes | * | 0.0186 |
| Male:CBD_U vs. Male:Sham_S | -1.323 | -3.204 to 0.5581 | No | ns | 0.3416 |
| Male:CBD_U vs. Male:CBD_S | -1.781 | -3.423 to -0.1391 | Yes | * | 0.0256 |
| Male:Sham_S vs. Male:CBD_S | -0.458 | -2.216 to 1.300 | No | ns | 0.9895 |

| Test details | Mean 1 | Mean 2 | Mean Diff. | SE of diff. | N1 | N2 | q | DF |
|---|---|---|---|---|---|---|---|---|
| Female:Sham_U vs. Female:CBD_U | 7.761 | 6.763 | 0.9976 | 0.5281 | 6 | 5 | 2.671 | 36 |
| Female:Sham_U vs. Female:Sham_S | 7.761 | 6.169 | 1.591 | 0.5035 | 6 | 6 | 4.469 | 36 |
| Female:Sham_U vs. Female:CBD_S | 7.761 | 6.235 | 1.525 | 0.5035 | 6 | 6 | 4.284 | 36 |
| Female:Sham_U vs. Male:Sham_U | 7.761 | 4.205 | 3.555 | 0.5281 | 6 | 5 | 9.521 | 36 |
| Female:Sham_U vs. Male:CBD_U | 7.761 | 4.269 | 3.492 | 0.5281 | 6 | 5 | 9.35 | 36 |
| Female:Sham_U vs. Male:Sham_S | 7.761 | 5.592 | 2.169 | 0.563 | 6 | 4 | 5.448 | 36 |
| Female:Sham_U vs. Male:CBD_S | 7.761 | 6.05 | 1.711 | 0.4852 | 6 | 7 | 4.986 | 36 |
| Female:CBD_U vs. Female:Sham_S | 6.763 | 6.169 | 0.5937 | 0.5281 | 5 | 6 | 1.59 | 36 |
| Female:CBD_U vs. Female:CBD_S | 6.763 | 6.235 | 0.5279 | 0.5281 | 5 | 6 | 1.414 | 36 |
| Female:CBD_U vs. Male:Sham_U | 6.763 | 4.205 | 2.558 | 0.5516 | 5 | 5 | 6.558 | 36 |
| Female:CBD_U vs. Male:CBD_U | 6.763 | 4.269 | 2.494 | 0.5516 | 5 | 5 | 6.395 | 36 |
| Female:CBD_U vs. Male:Sham_S | 6.763 | 5.592 | 1.171 | 0.585 | 5 | 4 | 2.831 | 36 |
| Female:CBD_U vs. Male:CBD_S | 6.763 | 6.05 | 0.7129 | 0.5107 | 5 | 7 | 1.974 | 36 |
| Female:Sham_S vs. Female:CBD_S | 6.169 | 6.235 | -0.06587 | 0.5035 | 6 | 6 | 0.185 | 36 |
| Female:Sham_S vs. Male:Sham_U | 6.169 | 4.205 | 1.964 | 0.5281 | 6 | 5 | 5.259 | 36 |
| Female:Sham_S vs. Male:CBD_U | 6.169 | 4.269 | 1.9 | 0.5281 | 6 | 5 | 5.089 | 36 |
| Female:Sham_S vs. Male:Sham_S | 6.169 | 5.592 | 0.5772 | 0.563 | 6 | 4 | 1.45 | 36 |
| Female:Sham_S vs. Male:CBD_S | 6.169 | 6.05 | 0.1192 | 0.4852 | 6 | 7 | 0.3473 | 36 |
| Female:CBD_S vs. Male:Sham_U | 6.235 | 4.205 | 2.03 | 0.5281 | 6 | 5 | 5.436 | 36 |
| Female:CBD_S vs. Male:CBD_U | 6.235 | 4.269 | 1.966 | 0.5281 | 6 | 5 | 5.265 | 36 |
| Female:CBD_S vs. Male:Sham_S | 6.235 | 5.592 | 0.6431 | 0.563 | 6 | 4 | 1.615 | 36 |
| Female:CBD_S vs. Male:CBD_S | 6.235 | 6.05 | 0.185 | 0.4852 | 6 | 7 | 0.5393 | 36 |
| Male:Sham_U vs. Male:CBD_U | 4.205 | 4.269 | -0.06366 | 0.5516 | 5 | 5 | 0.1632 | 36 |
| Male:Sham_U vs. Male:Sham_S | 4.205 | 5.592 | -1.387 | 0.585 | 5 | 4 | 3.352 | 36 |
| Male:Sham_U vs. Male:CBD_S | 4.205 | 6.05 | -1.845 | 0.5107 | 5 | 7 | 5.109 | 36 |
| Male:CBD_U vs. Male:Sham_S | 4.269 | 5.592 | -1.323 | 0.585 | 5 | 4 | 3.198 | 36 |
| Male:CBD_U vs. Male:CBD_S | 4.269 | 6.05 | -1.781 | 0.5107 | 5 | 7 | 4.933 | 36 |
| Male:Sham_S vs. Male:CBD_S | 5.592 | 6.05 | -0.458 | 0.5466 | 4 | 7 | 1.185 | 36 |

**Supplementary Material 5. Full Statistical Summary of *Slc29a1* gene expression in the inferior colliculus of GASH/Sal animals across chronic treatment, acoustic seizure stimulation and sex.**

| Three-way ANOVA | Ordinary | | | | |
|---|---|---|---|---|---|
| Alpha | 0.05 | | | | |
| | | | | | |
| Source of Variation | % of total variation | P value | P value summary | Significant? | |
| Sex | 51.05 | <0.0001 | **** | Yes | |
| Treatment | 0.01494 | 0.8978 | ns | No | |
| Condition | 14.81 | 0.0002 | *** | Yes | |
| Sex x Treatment | 1.882 | 0.1551 | ns | No | |
| Sex x Condition | 6.016 | 0.0135 | * | Yes | |
| Treatment x Condition | 0.02683 | 0.8633 | ns | No | |
| Sex x Treatment x Condition | 0.02196 | 0.8762 | ns | No | |
| | | | | | |
| ANOVA table | SS | DF | MS | F (DFn, DFd) | P value |
| Sex | 23.02 | 1 | 23.02 | F (1, 36) = 57.23 | P<0.0001 |
| Treatment | 0.006736 | 1 | 0.006736 | F (1, 36) = 0.01674 | P=0.8978 |
| Condition | 6.677 | 1 | 6.677 | F (1, 36) = 16.60 | P=0.0002 |
| Sex x Treatment | 0.8485 | 1 | 0.8485 | F (1, 36) = 2.109 | P=0.1551 |
| Sex x Condition | 2.713 | 1 | 2.713 | F (1, 36) = 6.744 | P=0.0135 |
| Treatment x Condition | 0.0121 | 1 | 0.0121 | F (1, 36) = 0.03008 | P=0.8633 |
| Sex x Treatment x Condition | 0.009901 | 1 | 0.009901 | F (1, 36) = 0.02461 | P=0.8762 |
| Residual | 14.48 | 36 | 0.4023 | | |
| | | | | | |
| Compare each cell mean with every other cell mean | | | | | |
| | | | | | |
| Number of families | 1 | | | | |
| Number of comparisons per family | 28 | | | | |
| Alpha | 0.05 | | | | |

| Tukey's multiple comparisons test | Mean Diff. | 95.00% CI of diff. | Significant? | Summary | Adjusted P Value |
|---|---|---|---|---|---|
| Female:Sham_U vs. Female:CBD_U | -0.2894 | -1.524 to 0.9455 | No | ns | 0.9945 |
| Female:Sham_U vs. Female:Sham_S | 0.3032 | -0.8742 to 1.481 | No | ns | 0.9902 |
| Female:Sham_U vs. Female:CBD_S | 0.02022 | -1.157 to 1.198 | No | ns | >0.9999 |
| Female:Sham_U vs. Male:Sham_U | 2.28 | 1.046 to 3.515 | Yes | **** | <0.0001 |
| Female:Sham_U vs. Male:CBD_U | 0.924 | -0.3109 to 2.159 | No | ns | 0.2692 |
| Female:Sham_U vs. Male:Sham_S | 1.96 | 0.6437 to 3.277 | Yes | *** | 0.0007 |
| Female:Sham_U vs. Male:CBD_S | 0.7317 | -0.4029 to 1.866 | No | ns | 0.4501 |
| Female:CBD_U vs. Female:Sham_S | 0.5926 | -0.6423 to 1.828 | No | ns | 0.7793 |
| Female:CBD_U vs. Female:CBD_S | 0.3096 | -0.9253 to 1.545 | No | ns | 0.9917 |
| Female:CBD_U vs. Male:Sham_U | 2.57 | 1.280 to 3.860 | Yes | **** | <0.0001 |
| Female:CBD_U vs. Male:CBD_U | 1.213 | -0.07639 to 2.503 | No | ns | 0.0778 |
| Female:CBD_U vs. Male:Sham_S | 2.25 | 0.8815 to 3.618 | Yes | *** | 0.0002 |
| Female:CBD_U vs. Male:CBD_S | 1.021 | -0.1730 to 2.215 | No | ns | 0.1407 |
| Female:Sham_S vs. Female:CBD_S | -0.283 | -1.460 to 0.8945 | No | ns | 0.9935 |
| Female:Sham_S vs. Male:Sham_U | 1.977 | 0.7423 to 3.212 | Yes | *** | 0.0002 |
| Female:Sham_S vs. Male:CBD_U | 0.6208 | -0.6141 to 1.856 | No | ns | 0.7376 |
| Female:Sham_S vs. Male:Sham_S | 1.657 | 0.3405 to 2.973 | Yes | ** | 0.0058 |
| Female:Sham_S vs. Male:CBD_S | 0.4285 | -0.7061 to 1.563 | No | ns | 0.9223 |
| Female:CBD_S vs. Male:Sham_U | 2.26 | 1.025 to 3.495 | Yes | **** | <0.0001 |
| Female:CBD_S vs. Male:CBD_U | 0.9038 | -0.3311 to 2.139 | No | ns | 0.2944 |
| Female:CBD_S vs. Male:Sham_S | 1.94 | 0.6235 to 3.256 | Yes | *** | 0.0008 |
| Female:CBD_S vs. Male:CBD_S | 0.7115 | -0.4231 to 1.846 | No | ns | 0.4856 |
| Male:Sham_U vs. Male:CBD_U | -1.356 | -2.646 to -0.06657 | Yes | * | 0.0334 |
| Male:Sham_U vs. Male:Sham_S | -0.3203 | -1.688 to 1.048 | No | ns | 0.9945 |
| Male:Sham_U vs. Male:CBD_S | -1.549 | -2.743 to -0.3545 | Yes | ** | 0.0041 |
| Male:CBD_U vs. Male:Sham_S | 1.036 | -0.3320 to 2.404 | No | ns | 0.2559 |
| Male:CBD_U vs. Male:CBD_S | -0.1923 | -1.386 to 1.002 | No | ns | 0.9995 |
| Male:Sham_S vs. Male:CBD_S | -1.228 | -2.507 to 0.04985 | No | ns | 0.0671 |

| Test details | Mean 1 | Mean 2 | Mean Diff. | SE of diff. | N1 | N2 | q | DF |
|---|---|---|---|---|---|---|---|---|
| Female:Sham_U vs. Female:CBD_U | 6.701 | 6.991 | -0.2894 | 0.3841 | 6 | 5 | 1.066 | 36 |
| Female:Sham_U vs. Female:Sham_S | 6.701 | 6.398 | 0.3032 | 0.3662 | 6 | 6 | 1.171 | 36 |
| Female:Sham_U vs. Female:CBD_S | 6.701 | 6.681 | 0.02022 | 0.3662 | 6 | 6 | 0.07811 | 36 |
| Female:Sham_U vs. Male:Sham_U | 6.701 | 4.421 | 2.28 | 0.3841 | 6 | 5 | 8.397 | 36 |
| Female:Sham_U vs. Male:CBD_U | 6.701 | 5.777 | 0.924 | 0.3841 | 6 | 5 | 3.403 | 36 |
| Female:Sham_U vs. Male:Sham_S | 6.701 | 4.741 | 1.96 | 0.4094 | 6 | 4 | 6.771 | 36 |
| Female:Sham_U vs. Male:CBD_S | 6.701 | 5.97 | 0.7317 | 0.3529 | 6 | 7 | 2.933 | 36 |
| Female:CBD_U vs. Female:Sham_S | 6.991 | 6.398 | 0.5926 | 0.3841 | 5 | 6 | 2.182 | 36 |
| Female:CBD_U vs. Female:CBD_S | 6.991 | 6.681 | 0.3096 | 0.3841 | 5 | 6 | 1.14 | 36 |
| Female:CBD_U vs. Male:Sham_U | 6.991 | 4.421 | 2.57 | 0.4011 | 5 | 5 | 9.06 | 36 |
| Female:CBD_U vs. Male:CBD_U | 6.991 | 5.777 | 1.213 | 0.4011 | 5 | 5 | 4.278 | 36 |
| Female:CBD_U vs. Male:Sham_S | 6.991 | 4.741 | 2.25 | 0.4255 | 5 | 4 | 7.477 | 36 |
| Female:CBD_U vs. Male:CBD_S | 6.991 | 5.97 | 1.021 | 0.3714 | 5 | 7 | 3.889 | 36 |
| Female:Sham_S vs. Female:CBD_S | 6.398 | 6.681 | -0.283 | 0.3662 | 6 | 6 | 1.093 | 36 |
| Female:Sham_S vs. Male:Sham_U | 6.398 | 4.421 | 1.977 | 0.3841 | 6 | 5 | 7.281 | 36 |
| Female:Sham_S vs. Male:CBD_U | 6.398 | 5.777 | 0.6208 | 0.3841 | 6 | 5 | 2.286 | 36 |
| Female:Sham_S vs. Male:Sham_S | 6.398 | 4.741 | 1.657 | 0.4094 | 6 | 4 | 5.723 | 36 |
| Female:Sham_S vs. Male:CBD_S | 6.398 | 5.97 | 0.4285 | 0.3529 | 6 | 7 | 1.717 | 36 |
| Female:CBD_S vs. Male:Sham_U | 6.681 | 4.421 | 2.26 | 0.3841 | 6 | 5 | 8.323 | 36 |
| Female:CBD_S vs. Male:CBD_U | 6.681 | 5.777 | 0.9038 | 0.3841 | 6 | 5 | 3.328 | 36 |
| Female:CBD_S vs. Male:Sham_S | 6.681 | 4.741 | 1.94 | 0.4094 | 6 | 4 | 6.701 | 36 |
| Female:CBD_S vs. Male:CBD_S | 6.681 | 5.97 | 0.7115 | 0.3529 | 6 | 7 | 2.852 | 36 |
| Male:Sham_U vs. Male:CBD_U | 4.421 | 5.777 | -1.356 | 0.4011 | 5 | 5 | 4.782 | 36 |
| Male:Sham_U vs. Male:Sham_S | 4.421 | 4.741 | -0.3203 | 0.4255 | 5 | 4 | 1.065 | 36 |
| Male:Sham_U vs. Male:CBD_S | 4.421 | 5.97 | -1.549 | 0.3714 | 5 | 7 | 5.897 | 36 |
| Male:CBD_U vs. Male:Sham_S | 5.777 | 4.741 | 1.036 | 0.4255 | 5 | 4 | 3.444 | 36 |
| Male:CBD_U vs. Male:CBD_S | 5.777 | 5.97 | -0.1923 | 0.3714 | 5 | 7 | 0.7323 | 36 |
| Male:Sham_S vs. Male:CBD_S | 4.741 | 5.97 | -1.228 | 0.3975 | 4 | 7 | 4.37 | 36 |

## Supplementary Material 5. Full Statistical Summary of *Cnr1* gene expression in the inferior colliculus of GASH/Sal animals across chronic treatment, acoustic seizure stimulation and sex.

| **Three-way ANOVA** | Ordinary | | | | |
|---|---|---|---|---|---|
| Alpha | 0.05 | | | | |
| | | | | | |
| Source of Variation | % of total variation | P value | P value summary | Significant? | |
| Sex | 43.69 | <0.0001 | **** | Yes | |
| Treatment | 2.199 | 0.0668 | ns | No | |
| Condition | 1.069 | 0.1955 | ns | No | |
| Sex x Treatment | 29.33 | <0.0001 | **** | Yes | |
| Sex x Condition | 2.065 | 0.0753 | ns | No | |
| Treatment x Condition | 0.8107 | 0.2582 | ns | No | |
| Sex x Treatment x Condition | 0.3707 | 0.4422 | ns | No | |
| | | | | | |
| ANOVA table | SS | DF | MS | F (DFn, DFd) | P value |
| Sex | 40.01 | 1 | 40.01 | F (1, 34) = 71.26 | P<0.0001 |
| Treatment | 2.014 | 1 | 2.014 | F (1, 34) = 3.587 | P=0.0668 |
| Condition | 0.9789 | 1 | 0.9789 | F (1, 34) = 1.743 | P=0.1955 |
| Sex x Treatment | 26.86 | 1 | 26.86 | F (1, 34) = 47.83 | P<0.0001 |
| Sex x Condition | 1.891 | 1 | 1.891 | F (1, 34) = 3.367 | P=0.0753 |
| Treatment x Condition | 0.7425 | 1 | 0.7425 | F (1, 34) = 1.322 | P=0.2582 |
| Sex x Treatment x Condition | 0.3395 | 1 | 0.3395 | F (1, 34) = 0.6046 | P=0.4422 |
| Residual | 19.09 | 34 | 0.5615 | | |
| | | | | | |
| Number of comparisons per family | 28 | | | | |
| Alpha | 0.05 | | | | |

| **Tukey's multiple comparisons test** | Mean Diff. | 95.00% CI of diff. | Significant? | Summary | Adjusted P Value |
|---|---|---|---|---|---|
| Female:Sham_U vs. Female:CBD_U | -0.3295 | -1.794 to 1.135 | No | ns | 0.9955 |
| Female:Sham_U vs. Female:Sham_S | 0.7217 | -0.7424 to 2.186 | No | ns | 0.7524 |
| Female:Sham_U vs. Female:CBD_S | 1.291 | -0.1046 to 2.687 | No | ns | 0.0867 |
| Female:Sham_U vs. Male:Sham_U | 3.828 | 2.364 to 5.293 | Yes | **** | <0.0001 |
| Female:Sham_U vs. Male:CBD_U | 3.006 | 1.542 to 4.470 | Yes | **** | <0.0001 |
| Female:Sham_U vs. Male:Sham_S | 1.687 | 0.1262 to 3.248 | Yes | * | 0.0266 |
| Female:Sham_U vs. Male:CBD_S | 1.038 | -0.3582 to 2.434 | No | ns | 0.2742 |
| Female:CBD_U vs. Female:Sham_S | 1.051 | -0.4780 to 2.580 | No | ns | 0.3667 |
| Female:CBD_U vs. Female:CBD_S | 1.621 | 0.1567 to 3.085 | Yes | * | 0.0215 |
| Female:CBD_U vs. Male:Sham_U | 4.158 | 2.629 to 5.687 | Yes | **** | <0.0001 |
| Female:CBD_U vs. Male:CBD_U | 3.335 | 1.806 to 4.864 | Yes | **** | <0.0001 |
| Female:CBD_U vs. Male:Sham_S | 2.016 | 0.3944 to 3.638 | Yes | ** | 0.0068 |
| Female:CBD_U vs. Male:CBD_S | 1.367 | -0.09679 to 2.831 | No | ns | 0.0814 |
| Female:Sham_S vs. Female:CBD_S | 0.5696 | -0.8945 to 2.034 | No | ns | 0.9087 |
| Female:Sham_S vs. Male:Sham_U | 3.107 | 1.578 to 4.636 | Yes | **** | <0.0001 |
| Female:Sham_S vs. Male:CBD_U | 2.284 | 0.7547 to 3.813 | Yes | *** | 0.0007 |
| Female:Sham_S vs. Male:Sham_S | 0.9651 | -0.6568 to 2.587 | No | ns | 0.5474 |
| Female:Sham_S vs. Male:CBD_S | 0.3161 | -1.148 to 1.780 | No | ns | 0.9965 |
| Female:CBD_S vs. Male:Sham_U | 2.537 | 1.073 to 4.001 | Yes | **** | <0.0001 |
| Female:CBD_S vs. Male:CBD_U | 1.714 | 0.2502 to 3.178 | Yes | * | 0.0126 |
| Female:CBD_S vs. Male:Sham_S | 0.3956 | -1.165 to 1.956 | No | ns | 0.9909 |
| Female:CBD_S vs. Male:CBD_S | -0.2535 | -1.649 to 1.142 | No | ns | 0.9988 |
| Male:Sham_U vs. Male:CBD_U | -0.8228 | -2.352 to 0.7064 | No | ns | 0.6647 |
| Male:Sham_U vs. Male:Sham_S | -2.142 | -3.764 to -0.5196 | Yes | ** | 0.0034 |
| Male:Sham_U vs. Male:CBD_S | -2.791 | -4.255 to -1.327 | Yes | **** | <0.0001 |
| Male:CBD_U vs. Male:Sham_S | -1.319 | -2.941 to 0.3032 | No | ns | 0.1826 |
| Male:CBD_U vs. Male:CBD_S | -1.968 | -3.432 to -0.5038 | Yes | ** | 0.0028 |
| Male:Sham_S vs. Male:CBD_S | -0.6491 | -2.210 to 0.9116 | No | ns | 0.8761 |

| Test details | Mean 1 | Mean 2 | Mean Diff. | SE of diff. | N1 | N2 | q | DF |
|---|---|---|---|---|---|---|---|---|
| Female:Sham_U vs. Female:CBD_U | 8.022 | 8.352 | -0.3295 | 0.4538 | 6 | 5 | 1.027 | 34 |
| Female:Sham_U vs. Female:Sham_S | 8.022 | 7.3 | 0.7217 | 0.4538 | 6 | 5 | 2.249 | 34 |
| Female:Sham_U vs. Female:CBD_S | 8.022 | 6.731 | 1.291 | 0.4326 | 6 | 6 | 4.221 | 34 |
| Female:Sham_U vs. Male:Sham_U | 8.022 | 4.194 | 3.828 | 0.4538 | 6 | 5 | 11.93 | 34 |
| Female:Sham_U vs. Male:CBD_U | 8.022 | 5.016 | 3.006 | 0.4538 | 6 | 5 | 9.368 | 34 |
| Female:Sham_U vs. Male:Sham_S | 8.022 | 6.335 | 1.687 | 0.4837 | 6 | 4 | 4.932 | 34 |
| Female:Sham_U vs. Male:CBD_S | 8.022 | 6.984 | 1.038 | 0.4326 | 6 | 6 | 3.392 | 34 |
| Female:CBD_U vs. Female:Sham_S | 8.352 | 7.3 | 1.051 | 0.4739 | 5 | 5 | 3.137 | 34 |
| Female:CBD_U vs. Female:CBD_S | 8.352 | 6.731 | 1.621 | 0.4538 | 5 | 6 | 5.052 | 34 |
| Female:CBD_U vs. Male:Sham_U | 8.352 | 4.194 | 4.158 | 0.4739 | 5 | 5 | 12.41 | 34 |
| Female:CBD_U vs. Male:CBD_U | 8.352 | 5.016 | 3.335 | 0.4739 | 5 | 5 | 9.952 | 34 |
| Female:CBD_U vs. Male:Sham_S | 8.352 | 6.335 | 2.016 | 0.5027 | 5 | 4 | 5.673 | 34 |
| Female:CBD_U vs. Male:CBD_S | 8.352 | 6.984 | 1.367 | 0.4538 | 5 | 6 | 4.262 | 34 |
| Female:Sham_S vs. Female:CBD_S | 7.3 | 6.731 | 0.5696 | 0.4538 | 5 | 6 | 1.775 | 34 |
| Female:Sham_S vs. Male:Sham_U | 7.3 | 4.194 | 3.107 | 0.4739 | 5 | 5 | 9.27 | 34 |
| Female:Sham_S vs. Male:CBD_U | 7.3 | 5.016 | 2.284 | 0.4739 | 5 | 5 | 6.815 | 34 |
| Female:Sham_S vs. Male:Sham_S | 7.3 | 6.335 | 0.9651 | 0.5027 | 5 | 4 | 2.715 | 34 |
| Female:Sham_S vs. Male:CBD_S | 7.3 | 6.984 | 0.3161 | 0.4538 | 5 | 6 | 0.9851 | 34 |
| Female:CBD_S vs. Male:Sham_U | 6.731 | 4.194 | 2.537 | 0.4538 | 6 | 5 | 7.907 | 34 |
| Female:CBD_S vs. Male:CBD_U | 6.731 | 5.016 | 1.714 | 0.4538 | 6 | 5 | 5.343 | 34 |
| Female:CBD_S vs. Male:Sham_S | 6.731 | 6.335 | 0.3956 | 0.4837 | 6 | 4 | 1.157 | 34 |
| Female:CBD_S vs. Male:CBD_S | 6.731 | 6.984 | -0.2535 | 0.4326 | 6 | 6 | 0.8287 | 34 |
| Male:Sham_U vs. Male:CBD_U | 4.194 | 5.016 | -0.8228 | 0.4739 | 5 | 5 | 2.455 | 34 |
| Male:Sham_U vs. Male:Sham_S | 4.194 | 6.335 | -2.142 | 0.5027 | 5 | 4 | 6.025 | 34 |
| Male:Sham_U vs. Male:CBD_S | 4.194 | 6.984 | -2.791 | 0.4538 | 5 | 6 | 8.698 | 34 |
| Male:CBD_U vs. Male:Sham_S | 5.016 | 6.335 | -1.319 | 0.5027 | 5 | 4 | 3.71 | 34 |
| Male:CBD_U vs. Male:CBD_S | 5.016 | 6.984 | -1.968 | 0.4538 | 5 | 6 | 6.133 | 34 |
| Male:Sham_S vs. Male:CBD_S | 6.335 | 6.984 | -0.6491 | 0.4837 | 4 | 6 | 1.898 | 34 |
