# Supplementary material for: Anticonvulsant effects, safety, and sex-dependent molecular signatures of cannabidiol treatment in a preclinical model of audiogenic epilepsy
Source: Front Pharmacol. 2026 Aug 7;17:1745696. doi: 10.3389/fphar.2026.1745696 (PMC13493383; doi:10.3389/fphar.2026.1745696)
Supplement: Supplementary file 5 [file DataSheet4.zip › Supplementary Material 4. Heatmap visualizer Z-score..html]

qPCR Expression Visualizer


qPCR Visualizer

Scaled ΔCt (sign-corrected · + = higher expression)

No data loaded

☀ Light mode

Data Input

Required columns: `Gene` `Group` `Condition` `Sex` `Scaled_dCt`  
Multiple rows per group = multiple samples. Group means are computed automatically.  
Sign convention: values are sign-corrected z-scores — **positive = higher expression**, **negative = lower expression**.

▶ Generate visualizations
Load dataset (6 genes · 262 samples)

Heatmap Options

Color scheme

Brown – White – Blue (paper)
Red – White – Blue
Orange – White – Teal
Warm Red – White – Dark Blue

Show

Samples + Group means
Samples only
Group means only

Samples + Mean per Sex (F / M)
Samples + Mean per Group (Sham / CBD)
Samples + Mean per Condition (U / S)
Samples + Grand mean (all groups)

Cell size

Small (36px)
Medium (48px)
Large (62px)

Show z-values

Off
On

Axis orientation

Genes = rows, Groups = columns
Genes = columns, Groups = rows

Group order — preset

Custom (drag below)
Control first (Sham → CBD)
Treatment first (CBD → Sham)
Unstimulated → Stimulated
Stimulated → Unstimulated
Female → Male
Male → Female
Alphabetical

Group order — drag to reorder

Bar Chart Options

Gene

Display

Samples + Group mean
Samples only
Group mean only

Color bars by

Sex
Group
Condition

Group order — preset

Custom (drag below)
Control first (Sham → CBD)
Treatment first (CBD → Sham)
Unstimulated → Stimulated
Stimulated → Unstimulated
Female → Male
Male → Female
Alphabetical

Group order — drag to reorder

Heatmap
Bar Chart
Summary Stats

⬛

Paste data and click **Generate visualizations**

Scaled ΔCt Heatmap — Sample cases & group means

Low (−)  
lower expression

High (+)  
higher expression
Hover for details

Female

Male

Group mean

📊

Paste data and click **Generate visualizations**

Scaled ΔCt — Sample cases & group means

📋

Paste data and click **Generate visualizations**

Overview

Group Means

| Gene | Group | Condition | Sex | n | Mean Scaled ΔCt | SD |
| --- | --- | --- | --- | --- | --- | --- |

All Sample Cases

| Gene | Group | Condition | Sex | Scaled ΔCt |
| --- | --- | --- | --- | --- |
